# Palladium-Catalyzed Synthesis and Isolation of Functionalized Allylboronic Acids: Selective, Direct Allylboration of Ketones**

**DOI:** 10.1002/anie.201207951

**Published:** 2012-11-19

**Authors:** Mihai Raducan, Rauful Alam, Kálmán J Szabó

**Affiliations:** Department of Organic Chemistry, Stockholm UniversityStockholm (Sweden) E-mail: kalman@organ.su.se Homepage: http://www.organ.su.se/ks

**Keywords:** allylic compounds, boron, homogeneous catalysis, ketones, palladium

Allyl boronates are very important reagents in advanced organic synthesis for the allylation of carbonyl compounds^1^ and in cross-coupling^2^ reactions. A practically unrivalled property of allylboronates is their highly regio- and stereoselective addition to carbonyl compounds to afford homoallylic alcohols.1a–d, 3 This high selectivity is mainly based on two factors: 1) Allylboronates are configurationally stable, and unlike many allyl metal compounds^4^ (such as allyl-Grignard reagents, allyl lithium compounds or allylboranes) do not undergo metallotropic rearrangement and *E*/*Z* isomerization at ambient conditions. 2) The six-membered ring transition state (TS) of the allylboration of carbonyl compounds is highly conformationally constrained, which leads to a strong differentiation in the stereoselection process.3a, 5 Method development for broadening the number of accessible functionalized allyl boronates is one of the most important challenges in organoboron chemistry.

In allylboration reactions with allylboronic esters, allyl pinacolborate (allyl-Bpin) reagents are most frequently applied as the allyl source,1a–d as allyl-Bpins can be easily handled and the functionalized derivatives can be obtained by metal-catalyzed borylation of the allyl precursors with bis(pinacolato)diboron (B_2_pin_2_) as the boronate source.^6^ Problems in synthetic applications arise from the relatively low reactivity of allyl-Bpin compounds towards most carbonyl compounds. Although aldehydes easily react with allyl-Bpin without catalysts, ketones do not. There are very few attempts at the direct allylation of ketones by functionalized allyl-Bpins or their ester analogues reported in the literature. The reactions that have been successful require extreme conditions (for example, 8000 bar and 3 days), which leads to an unselective process;^7^ therefore, a selective allylboration requires catalytic conditions.^8^ Some time ago we reported the application of diboronic acid **1**^9^ (now commercially available) as a boronate source1e, 10 for the Pd-catalyzed borylation of allylic alcohols (**3**) to obtain allylboronic acids (**4**; Scheme 1).6c,d However, owing to their instability, allylboronic acids could not be isolated and studied in a pure form. Therefore, the synthetic potential of pure allylboronic acids remained unexploited.

**Scheme 1:**
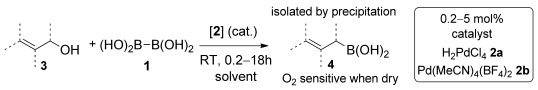
Synthesis and isolation of allylboronic acids.

We have now substantially modified the catalyst (**2**) and the reaction conditions of the process, which allowed the isolation of these extremely useful reagents (see Schemes 1 and 2, and Table 1). The outstanding synthetic utility of allylboronic acids is demonstrated by their direct (uncatalyzed) reactions with ketones under mild conditions, which proceeds with remarkably high regio- and stereoselectivity (see Scheme 3 and Table 2). As we have previously noted,6c,d allylboronic acids are fairly stable under ambient conditions, as long as they are dissolved in coordinating solvents. When these solvents are completely removed, rapid decomposition occurs. Careful experiments under inert conditions have shown that boroxine formation from **4** (which can be observed by ^1^H NMR) leads to extreme oxygen sensitivity (Scheme 2). Formation of boroxines from organoboronic acids is well known.1b, 11 However, in the case of arylboronic acids the corresponding boroxine is usually still air stable. We could not find any previous studies on boroxines formed from allylboronic acids. We have found that all of the studied allylboronic acids **4 a**–**l** become very oxygen sensitive after drying, but they can be kept in a glove box for a couple of weeks without extensive decomposition. The thermal stability of the allylboronic acids is very high. For example, heating **4 e** in dry degassed THF at 70 °C for 18 h does not lead to borotropic rearrangement. After careful optimization, we were able to find reaction conditions which are suitable for the synthesis of a wide range of allylic boronic acids with aromatic (**4 a**–**e**) and aliphatic (**4 f**–**l**) substituents, including both acyclic (**4 a**–**g** and **4 j**–**k**) and cyclic (**4 h**–**i, 4 l**) products. The allylboronic acids are formed with remarkably high regio- and stereoselectivity. We get perfect stereoselectivity, with the exception of **4 d**, which was formed as a 5:1 (*E*/*Z*) mixture. Notably, both geraniol **3 j** and nerol **3 k** underwent reaction without *E*/*Z* isomerization (Table 1, entries 10 and 11), with the double bond geometry remaining unchanged under the borylation conditions.

**Scheme 2:**
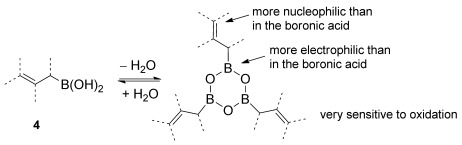
Boroxine formation upon drying.

**Table 1:** Synthesis and isolation of allylboronic acids.^[a]^

Entry	Substrate	Cat.^[b]^ (mol %)	Solvent^[c]^ (molarity)	*t* [h]	Product	Yield^[d]^ [%]
1	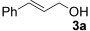	**2 a** (0.5)	MeOH (1.0)	18	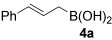	61^[e]^
2	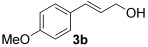	**2 a** (0.5)	MeOH (1.0)	0.2	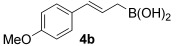	80
3	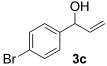	**2 a** (0.2)	MeOH (1.0)	2	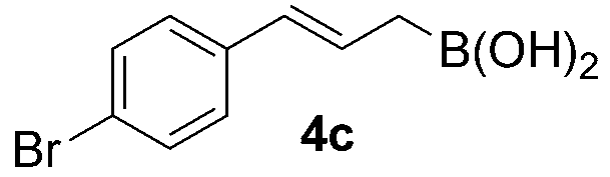	71
4	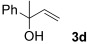	**2 a** (0.2)	DMSO/H_2_O 3:2 (1.0)	14	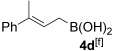	55
5	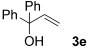	**2 a** (2.0)	DMSO/H_2_O 4:1 (1.0)	13	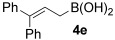	71
6	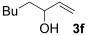	**2 a** (0.2)	MeOH (1.0)	1^[g]^	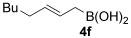	51
7	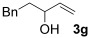	**2 a** (0.3)	MeOH (1.0)	1^[g]^	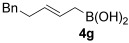	50
8	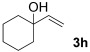	**2 a** (0.5)	DMSO/H_2_O 3:1 (1.0)	18	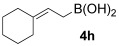	68
9	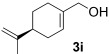	**2 b** (5.0)	DMSO/H_2_O 9:1 (0.4)	0.2	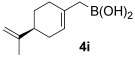	67
10	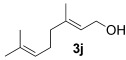	**2 a** (5.0)	DMSO/H_2_O 4:1 (0.5)	18	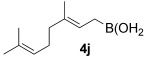	77^[h]^
11	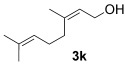	**2 a** (5.0)	DMSO/H_2_O 4:1 (0.5)	18	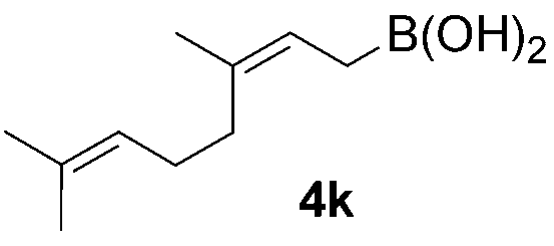	79^[h]^
12	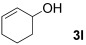	**2 b** (5.0)	DMSO/H_2_O 9:1 (1.0)	1	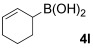	25

[a] Unless otherwise stated, Pd-catalyst **2 a** or **2 b** (0.2–5 mol %) and diboronic acid **1** (2.4 mmol) were added to allylic alcohol **3** (2 mmol) in the given solvent. After filtration, **4** was precipitated with degassed brine. [b] Catalyst loading (mol %) is given in parentheses. [c] The molar concentration of **3** in the given solvent is in parenthesis. [d] Yield of isolated product. [e] 65 % yield was obtained when the reaction was performed on a gram scale using 6 mmol of **3 a**. [f] 5:1 *E*/*Z* ratio. [g] The reactions were performed at 0 °C. [h] The product was isolated by extraction. The yield was determined by ^1^H NMR spectroscopy using naphthalene as an internal standard.

We used two different catalysts, **2 a** and **2 b**. Catalyst **2 a**^12^ was simply prepared by dissolving PdCl_2_ in aq. HCl, whereas **2 b** is commercially available and was previously applied^13^ in the synthesis of allylsilanes and allyl-Bpin derivatives. Catalyst **2 a** was less active, but more stable than **2 b**. Catalysts with phosphine ligands and Pd_2_(dba)_3_ (dba=dibenzylideneacetone) were ineffective under the applied conditions. Reactions in neat MeOH (Table 1, entries 1–3, 5–7) usually proceeded rapidly with low catalyst loadings (0.2–0.5 mol %). However, for sterically crowded substrates the borylation process was relatively slow, resulting in the decomposition of **1**, and the reduction of the allylic alcohols. In these cases, we replaced MeOH with a DMSO/H_2_O mixture, which always led to a slower reaction, but suppressed these undesired side reactions. The addition of DMSO was also useful for stabilizing the forming Pd^0^ species, and thus reducing the rate of deactivation of the Pd-catalyst. For some relatively unreactive substrates we used a combination of catalyst **2 b** and DMSO/H_2_O (entries 9 and 12) to optimize the borylation rate and reduce the formation of by-products. The concentration of the substrate was also an important factor. In some reactions the products underwent protodeborylation when the concentration was too high (see the molarity data in Table 1). Furthermore, precipitation of the products was initiated by the addition of brine, and thus the yield of isolated product was also dependent on the ratio of the organic solvent to water during the purification stage. Geranyl **4 j** and neryl **4 k** boronic acids could not be forced to precipitate, and therefore these compounds were separated by extraction into chloroform. Boronic acid **4 l** had similar solubility in both of the applied organic solvents (MeOH and DMSO) and in water. In this case (entry 12) the yield of the pure isolated product was poor, even though the borylation reaction was very selective. The reaction is easily scalable; the synthesis of cinnamylboronic acid **4 a** was repeated on a gram (6 mmol) scale without a significant change in the yield of isolated product (entry 1).

To demonstrate the synthetic utility of the functionalized boronic acids, we performed the allylation of ketones **5 a**–**c** (Table 2). These reactions were conducted either in dry THF or chloroform without any additives. As mentioned above, allyl-Bpin or other allylboronic esters are inefficient for the direct allylation of ketones. However, allylboronic acids **4 a** and **4 j**–**k** reacted with amazingly high stereoselectivity. The geranyl **4 j** and neryl **4 k** boronic acids very cleanly afforded the epimeric products **6 f** and **6 g** with the acetophenone derivative **5 c** (entries 6–7). Most of the reactions were conducted at room temperature, only the allylation of sterically crowded ketone **5 d** required elevated temperature (entry 4). As expected, the pinacolester of **4 a** (cinnamyl-Bpin) does not react at all with ketones under the above reaction conditions. Some of the above reactions have been performed using allylboronic esters^14^ or allyl stannanes^15^ in the presence of catalysts, allylaluminum,^16^ or titanocene^17^ compounds. However, the reported selectivities were the same as, or in several cases even lower than, with allylboronic acids. For example, the reactions of **5 b**–**d** with **4 a** (entries 2–4) resulted in a single diasteromer, whereas in analogous reactions using other allylating methods, the formation of small amounts of the other diastereomers was also reported. It can be concluded that the legendary high selectivity of the direct allylation of aldehydes with allyl-Bpin derivatives is also inherited by the direct allylboration of ketones by allylboronic acids. The mechanism of the allylboration of carbonyl compounds with allyl-Bpin and allylboronic acids most probably takes place through similar stereochemistry, as the stereochemistry in **6 b**–**d** and **6 f**–**g** is in line with formation via a Type I TS (Scheme 3).

**Scheme 3:**
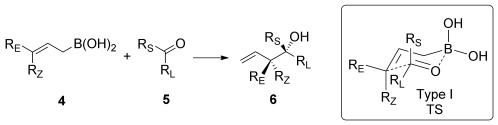
Allylboration of ketones.

**Table 2:** Reaction of allylboronic acids with ketones.^[a]^

Entry	Boronic acid	Ketone	Solvent	*t* [h]	Product	Yield [%]^[b]^
1	**4 a**	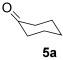	THF	1	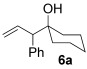	86
2	**4 a**	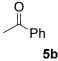	THF	24	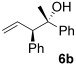	89
3	**4 a**	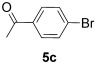	THF	14	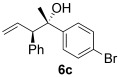	91
4	**4 a**	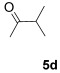	THF^[c]^	22	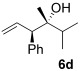	90
5	**4 j**	**5 a**	CHCl_3_^[d]^	18	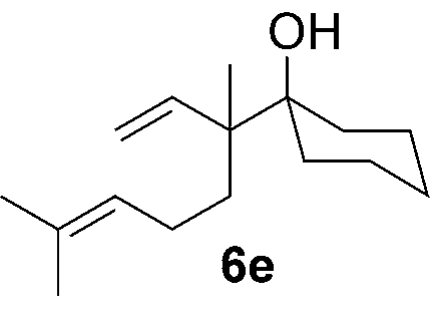	96
6	**4 j**	**5 c**	CHCl_3_^[d]^	18	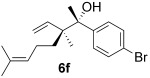	94^[e]^
7	**4 k**	**5 c**	CHCl_3_^[d]^	18	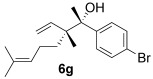	76^[f]^

[a] Unless otherwise stated, allyl boronic acid **4** (0.24–0.8 mmol) was reacted with ketone **5** (0.2–0.4 mmol) at RT in THF or CHCl_3_ for the allotted reaction time, affording a single diastereomer. [b] Yield of isolated product. [c] The reaction was performed at 60 °C. [d] Performed in the presence of 4 Å molecular sieves. [e] d.r.=98:2. [f] d.r.=99:1.

The much higher reactivity of allylboronic acids toward ketones (vs. allyl-Bpin derivatives) can be explained by the sterically less demanding nature of the B(OH)_2_ group relative to the Bpin group. Considering the close C=O–B contact in the TS5b (which is probably important for the very high stereoselectivity) the steric demand of the groups attached to the boron atom is probably essential for the success of the allylation reaction. We have noticed that in the presence of water or MeOH the allylboration of ketones with **4** is strongly inhibited. That was the reason6d for the failure of previous attempts to react allylboronic acids with ketones in a one-pot sequence with the borylation reaction (which requires the presence of either water or MeOH). Solvents may coordinate to the empty p_π_ orbital of B(OH)_2_ and thus compete with the oxygen of the carbonyl group in the TS (Scheme 3). An alternative explanation for the remarkably high reactivity of **4 a**–**k** with ketones could be that boroxine is formed prior to the allylation (Scheme 2), and the boroxine is more reactive than the allylboronic acid itself. In a boroxine the B/O ratio is 1:1, whereas in B(OH)_2_ this ratio is 1:2. Accordingly, the empty p_π_ orbital of the boron atom in a B(OH)_2_ group receives more electron density from the adjacent oxygen atoms than the corresponding boron atom in a boroxine. As a consequence, the Lewis acidity of a boroxine is higher than that of the corresponding allylboronic acid or allyl-Bpin derivative.

In summary, we have described a synthetically useful method for the preparation and isolation of functionalized allylboronic acids. In this Pd-catalyzed method, allylic alcohols are used as substrates and commercially available diboronic acid as B(OH)_2_ source. The products were usually isolated by precipitation under inert conditions. For the first time we have demonstrated that functionalized allylboronic acids react directly with ketones under mild conditions with an amazingly high regio- and stereoselectivity. Our method will hopefully open new synthetic routes in advanced organic synthesis and natural product synthesis,^18^ where highly selective allylation reactions without interference from strong Lewis acids are of paramount importance.

## Experimental Section

In a typical reaction, Pd-catalyst **2 a** or **2 b** (0.2–5 mol %) and diboronic acid **1** (2.4 mmol) were added to an allylic alcohol (2.0 mmol) dissolved in the appropriate solvent and stirred at room temperature for the allotted reaction time (see Table 1). After filtration of the reaction mixture, degassed brine was added under Ar; after stirring for 18 h, the precipitated solid was filtered off. The resulting boronic acid was washed with degassed water, dried, and stored under Ar.

## Supplementary material

Detailed facts of importance to specialist readers are published as ”Supporting Information”. Such documents are peer-reviewed, but not copy-edited or typeset. They are made available as submitted by the authors.
