# Supplementary material for: Palladium-Catalyzed Synthesis and Isolation of Functionalized Allylboronic Acids: Selective, Direct Allylboration of Ketones
Source: Angew Chem Int Ed Engl. 2012 Nov 19;51(52):13050–3. doi: 10.1002/anie.201207951 (PMC3556694; doi:10.1002/anie.201207951)

Angewandte Chemie
Eine Zeitschrift der Gesellschaft Deutscher Chemiker

Supporting Information
© Wiley-VCH 2012
69451 Weinheim, Germany

Palladium-Catalyzed Synthesis and Isolation of Functionalized Allylboronic Acids: Selective, Direct Allylboration of Ketones**

*Mihai Raducan, Rauful Alam, and Kálmán J. Szabó**

anie_201207951_sm_miscellaneous_information.pdf

Contents:

General Information	2
Experimental Procedure and Spectral Data	2-12
References	13
^{1}H and ^{13}C NMR spectra	14-69

General Information

Diboronic acid **1** was purified by washing with dioxane as shown below. NMR solvents used for the characterization of new compounds ($CDCl_3$, DMSO-d_6) were stored over molecular sieves (4Å) in an Ar filled glovebox. Molecular sieves 4Å (pellets) were activated by several microwave heating/vacuum/Ar cycles then stored in an Ar filled glovebox. All other chemicals and solvents were obtained from commercial sources and used as received. ^{1}H NMR, ^{13}C NMR and ^{11}B NMR spectra were recorded using 400 MHz or 500 MHz spectrometers. Chemical shifts are reported using the residual solvent peak as internal standard.[1] High resolution mass data (HRMS) were obtained using ESI technique. For column chromatography, silica gel (35-70 microns) was used.

Experimental Procedures and Spectral Data

Purification of diboronic acid 1. Crude diboronic acid **(1)** (10 g, <90% purity) was suspended into dioxane (40 mL) and the mixture was stirred under ambient conditions for 2 hours. The product was filtered off, washed with 10 mL water and thoroughly air-dried yielding 7.1-8.4 g of pure (98% - 100%) $B_2(OH)_4$ (**1**). The content of H_3BO_3 (<2%) and excess water (<1%) was determined by ^{1}H NMR of DMSO-d_6 solutions, taking into account the self- condensation equilibrium shown below.[2] ^{1}H NMR (400 MHz, DMSO-d_6): δ 8.84 (s, 4H, $B_6(OH)_4$), 8.61 (s, 4H, $B_4(OH)_4$), 7.58 (s, 4H, $B_2(OH)_4$), 6.50 (s, 3H, $B(OH)_3$), 3.32 (s, 2H, H_2O); ^{11}B NMR (128 MHz, DMSO-d_6): δ 31.9 (br s).

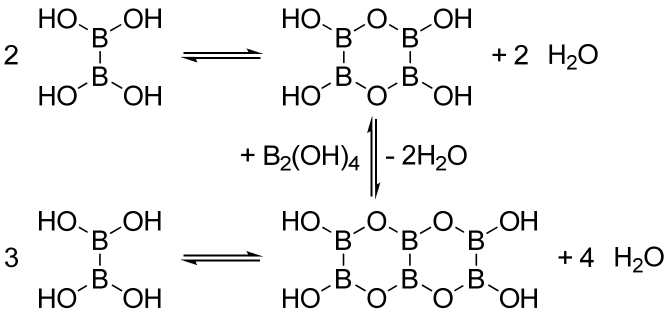

Preparation of H_2PdCl_4 (2a). $PdCl_2$ (54 mg, 0.30 mmol) was weighed in a GC vial and then aqueous HCl 0.9 M (1 mL) was added. The vial was capped and the

mixture was stirred at r.t. overnight. The resulting aqueous H_2PdCl_4 0.3 M solution was stored under ambient conditions and used as such.

General procedure for the synthesis of allyl boronic acids 4a-i and 4l (Table 1). To a solution of the allylic alcohol **3** (2.0 mmol) in the solvent mixture shown in Table 1, the palladium catalyst (**2a** or **2b**, 0.2-5 mol%) and diboronic acid (**1**, 1.2 equivalents) were successively added and the mixture was stirred vigorously. From time to time, aliquots from the reaction mixture were dissolved into $CDCl_3$ or DMSO-d_6 and analized by ^{1}H NMR. After the allotted times in Table 1 the conversion was 95-100%. Then (after 1-2 hours) the mixture was filtered through a HPLC Teflon filter (0.2 µm) into an Ar filled Schlenk tube containing a magnetic stir bar. The precipitant (degassed aqueous NaCl 16% solution or degassed H_2O) was added (3-4 times the initial reaction volume) and the mixture was stirred overnight. The solid was separated by filtration under Ar (see the picture of the filtration equipment below). The boronic acid was then washed with degassed H_2O (one reaction volume) and carefully dried by several vacuum/Ar plug cycles (i.e. by sudden opening of the Ar line). The washing/drying cycles were done 2-7 times. All but the last washing was also used to transfer the remaining precipitate from the Schlenk tube. Finally the product was briefly dried under vacuum and then stored in an Ar filled glove box. The water content (if any) was determined from the ^{1}H NMR spectrum of the product dissolved in dry DMSO-d_6. For the determination of the H_3BO_3 content (if any), a drop of water was added to the DMSO-d_6 solution. The ^{1}H NMR, ^{11}B NMR and ^{13}C NMR shift values reported below for the allylboronic acids correspond to NMR spectra recorded in wet DMSO-d_6 solutions. When the ^{1}H-NMR spectra is recorded in dry DMSO-d_6 (see below) the signals for both the allylboronic acid and the corresponding boroxine are observed (compare for example the spectra recorded for **4a** in dry and wet DMSO-d_6 on pages 16 and 17, respectively).

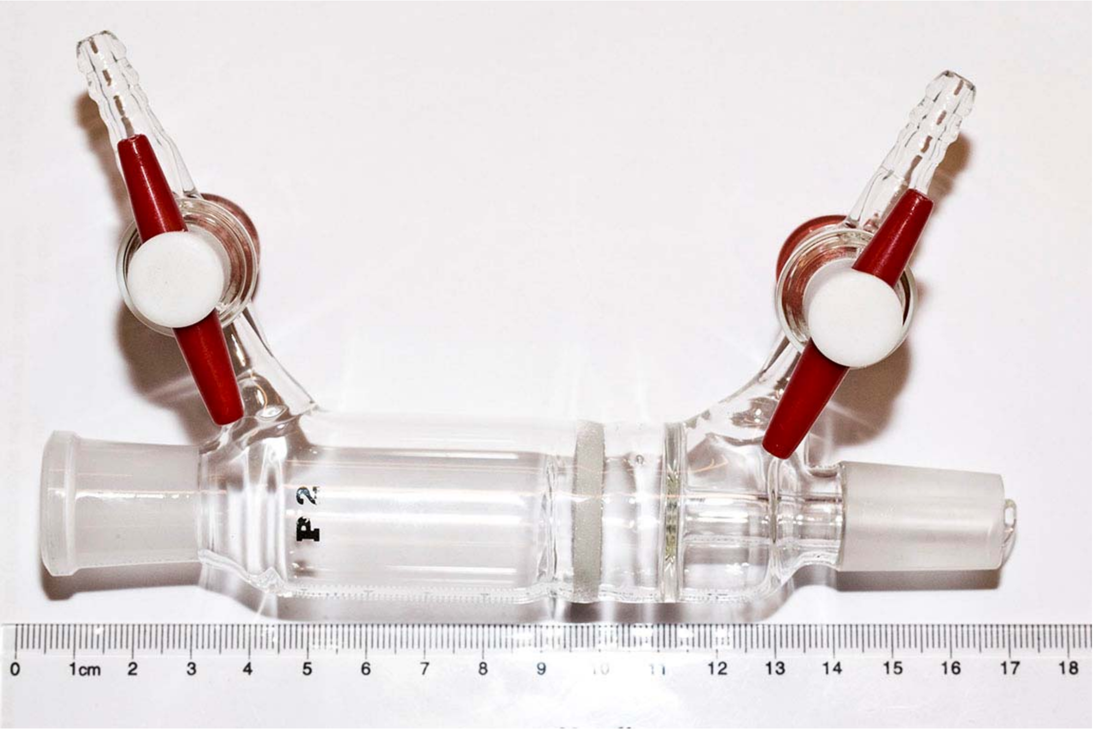

Equipment used for the inert (Ar) filtration of allylboronic acids **4a-i** and **4l**.

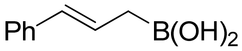

Cinnamylboronic acid (4a) was prepared according to the general procedure from **3a** (2.0 mmol), **2a** (0.3 M, 33 µL) and **1** (2.4 mmol) in MeOH (2 mL) using aqueous NaCl 16% as the precipitant (6 mL) and washing with H_2O (2x2 mL). The product was obtained as a white solid (198 mg, 61% yield, 99% purity), containing a trace amount of H_3BO_3 (1%). ^{1}H NMR (400 MHz, DMSO-d_6): δ 7.69 (s, 2H), 7.32-7.24 (m, 4H), 7.16-7.12 (m, 1H), 6.37 (dt, J = 15.6, 7.8 Hz, 1H), 6.23 (d, J = 15.8 Hz, 1H), 1.70 (dd, J = 7.7, 1.1 Hz, 2H); ^{13}C NMR (101 MHz, DMSO-d_6): δ 138.1 (C), 129.3 (CH), 128.5 (CH), 128.2 (CH), 126.3 (CH), 125.4 (CH), 21.8 (br s, CH_2); ^{11}B NMR (128 MHz, DMSO-d_6): δ 31.1 (br s).

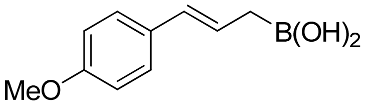

(E)-3-(4-Methoxyphenyl)allylboronic acid (4b) was prepared according to the general procedure from **3b** (2.0 mmol), **2a** (0.3 M, 33 µL) and **1** (2.4 mmol) in MeOH (2 mL) using aqueous NaCl 16% as the precipitant (8 mL) and washing with H_2O (7x2 mL). The product was obtained as a white solid (321 mg, 80% yield, 96% purity), containing a trace amount of H_2O (4%). ^{1}H NMR (400 MHz, DMSO-d_6): δ 7.64 (s, 2H), 7.25-7.22 (m, 2H), 6.86-6.83 (m, 2H), 6.24-6.13 (m, 2H), 3.72 (s, 3H), 1.65 (d, J = 6.2 Hz, 2H); ^{13}C NMR (101 MHz, DMSO-d_6): δ 157.9 (C), 130.9 (C), 127.7 (CH), 126.7 (CH),

126.5 (CH), 113.9 (CH), 55.1 (CH_3), 21.6 (br s, CH_2); ^{11}B NMR (128 MHz, DMSO-d_6): δ 30.8 (br s).

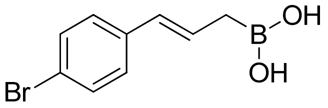

(E)-(3-(4-Bromophenyl)allyl)boronic acid (4c) was prepared according to the general procedure from **3c** (2.0 mmol), **2a** (0.3 M, 13 µL) and **1** (2.4 mmol) in MeOH (2 mL) using aqueous NaCl 16% as the precipitant (8 mL) and washing with H_2O (3x2 mL). The product was obtained as a light yellow solid (350 mg, 71% yield, >99% purity). ^{1}H NMR (500 MHz, DMSO-d_6): δ 7.74 (s, 2H), 7.49 (d, J = 8.4, 1H), 7.32 (d, J = 8.4, 1H), 6.49-6.42 (m, 1H), 6.25 (d, J = 15.7 Hz, 1H), 1.73 (d, J = 7.7 Hz, 2H); ^{13}C NMR (126 MHz, DMSO-d_6): δ 137.4 (Ar C), 131.3 (Ar CH), 130.7 (CH), 127.4 (Ar CH), 127.0 (CH), 118.9 (Ar C), 21.9 (br s, CH_2); ^{11}B NMR (128 MHz, DMSO-d_6): δ 31.4 (br s).

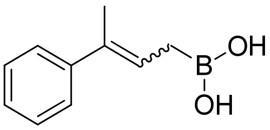

(3-Phenylbut-2-en-1-yl)boronic acid (4d) was prepared according to the general procedure from **3d** (2 mmol), **2a** (0.3 M, 13 µL) and **1** (2.4 mmol) in DMSO/H_2O (1.2 mL/0.8 mL) using aqueous NaCl 16% as the precipitant (8 mL) and washing with H_2O (3x2 mL), then the solid with H_2O (2x2 mL). The product was obtained as a white solid (196 mg, 55% yield, 97% purity) containing a trace amount of boric acid (3%). ^{1}H NMR for *E* isomer (400 MHz, $CDCl_3$): δ 7.61 (s, 2H), 7.37-7.15 (m, 5H), 5.98 (dt, J = 8.2, 1.2 Hz, 1H), 1.94 (s, 3H), 1.66 (d, J = 8.2 Hz, 2H); ^{13}C NMR for *E* isomer (101 MHz, $CDCl_3$): δ 143.7 (C), 131.8 (C), 128.2 (CH), 126.1 (C), 126.0 (CH), 125.2 (CH), 18.0 (br s, CH_2), 15.3 (CH_3); ^{1}H NMR for *Z* isomer_Selected Peaks (400 MHz, $CDCl_3$): δ 7.52 (s, 2H), 5.61 (t, J = 8.2 Hz, 1H), 1.96 (s, 3H), 1.44 (d, J = 7.5 Hz, 2H); ^{13}C NMR for *Z* isomer (101 MHz, $CDCl_3$): δ 141.8, 132.8, 128.1, 128.0, 126.3, 124.9, 25.4; ^{11}B NMR (128 MHz, DMSO-d_6): δ 30.9 (br s).

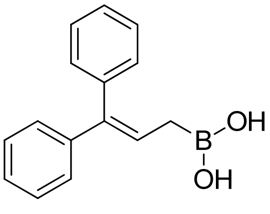

(3,3-Diphenylallyl)boronic acid (4e) was prepared according to the general procedure from **3e** (2 mmol), **2a** (0.3 M, 128 µL) and **1** (2.4 mmol) in DMSO/H_2O (1.6 mL/0.4 mL mL) using aqueous NaCl 16% as the precipitant (8 mL) and washing with H_2O (3x2 mL), then the solid with H_2O (2x2 mL). The product was obtained as a white solid (340 mg, 71% yield, >99% purity). ^{1}H NMR (400 MHz,

DMSO-d_6): δ 7.65 (s, 2H), 7.41-7.12 (m, 10H), 6.30 (t, J = 8.4, 1H), 1.58 (d, J = 8.4, 2H); ^{13}C NMR (126 MHz, DMSO-d_6): δ 143.4 (C), 140.5 (C), 139.1 (C), 130.3 (Ar CH), 128.8 (Ar CH), 128.6 (Ar CH), 128.5 (Ar CH), 127.2 (CH), 127.1 (Ar CH), 126.8 (Ar CH), 19.5 (br s, CH_2); ^{11}B NMR (128 MHz, DMSO-d_6): δ 31.3 (br s).

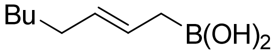

(E)-Oct-2-enylboronic acid (4f) was prepared according to the general procedure from **3f** (2.0 mmol), **2a** (0.3 M, 13 µL) and **1** (2.4 mmol) in MeOH (2 mL) using H_2O as the precipitant (8 mL) and washing with H_2O (2 mL). The product was obtained as a white solid (186 mg, 51% yield, 85% purity), containing a trace amount of H_3BO_3 (1%) and H_2O (14%). On prolonged drying (>15 minutes) the product turned into an oil that could not be recovered from the frit. ^{1}H NMR (400 MHz, DMSO-d_6): δ 7.48 (s, 2H), 5.44 (dtt, J = 15.0, 7.5, 1.3 Hz, 1H), 5.21 (dtt, J = 15.0, 6.8, 1.4 Hz, 1H), 1.90 (q, J = 6.7 Hz, 2H), 1.44-1.42 (m, 2H), 1.32-1.17 (m, 6H), 0.85 (t, J = 7.0 Hz, 3H); ^{13}C NMR (101 MHz, DMSO-d_6): δ 128.4 (CH), 127.5 (CH), 32.2 (CH_2), 30.9 (CH_2), 29.0 (CH_2), 22.0 (CH_2), 20.9 (br s, CH_2), 14.0 (CH_3); ^{11}B NMR (128 MHz, DMSO-d_6): δ 31.0 (br s).

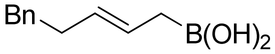

(E)-5-Phenylpent-2-enylboronic acid (4g) was prepared according to the general procedure from **3g** (2.0 mmol), **2a** (0.3 M, 20 µL) and **1** (2.4 mmol) in MeOH (2 mL) using H_2O the precipitant (8 mL) and washing with H_2O (4x2 mL). The product was obtained as a grey solid (192 mg, 50% yield, 99% purity), containing a trace amount of H_3BO_3 (1%). ^{1}H NMR (400 MHz, DMSO-d_6): δ 7.50 (s, 2H), 7.28-7.24 (m, 2H), 7.19-7.13 (m, 3H), 5.50 (dtt, J = 15.1, 7.6, 1.3 Hz, 1H), 5.27 (dtt, J = 15.0, 6.7, 1.5 Hz, 1H), 2.61-2.57 (m, 2H), 2.24-2.18 (m, 2H), 1.44-1.42 (m, 2H); ^{13}C NMR (101 MHz, DMSO-d_6): δ 142.0 (C), 128.4 (CH), 128.3 (CH), 128.1 (CH), 127.7 (CH), 125.7 (CH), 35.7 (CH_2), 34.3 (CH_2), 20.9 (br s, CH_2); ^{11}B NMR (128 MHz, DMSO-d_6): δ 31.2 (br s).

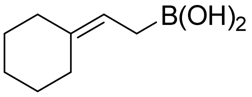

2-Cyclohexylideneethylboronic acid (4h) was prepared according to the general procedure from **3h** (2.0 mmol), **2a** (0.3 M, 33 µL) and **1** (2.4 mmol) in DMSO/H_2O (1.5 mL/0.5 mL) using aqueous NaCl 16% as the precipitant (8 mL) and washing with H_2O (5x2 mL). The product was obtained as a grey solid (211 mg, 68% yield, >99% purity), containing a trace amount of H_3BO_3 (<1%). ^{1}H NMR (400 MHz, DMSO-d_6): δ 7.43 (s, 2H), 5.15 (t, J

= 7.9 Hz, 1H), 2.06-1.97 (m, 4H), 1.50-1.37 (m, 8H); ^{13}C NMR (101 MHz, DMSO-d_6): δ 136.3 (C), 118.2 (CH), 36.8 (CH_2), 28.3 (CH_2), 28.0 (CH_2), 27.2 (CH_2), 26.6 (CH_2), 15.6 (br s, CH_2); ^{11}B NMR (128 MHz, DMSO-d_6): δ 30.9 (br s).

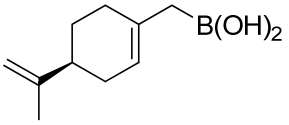

(S)-(4-(Prop-1-en-2-yl)cyclohex-1-enyl)methylboronic acid (4i) was prepared according to the general procedure from **3i** (2.0 mmol), **2b** (0.1 mmol) and **1** (2.4 mmol) in DMSO/H_2O (4.5 mL/0.5 mL) using aqueous NaCl 16% as the precipitant (20 mL) and washing with H_2O (3x5 mL). The product was obtained as an off white fluffy solid (241 mg, 67% yield, >99% purity). ^{1}H NMR (400 MHz, DMSO-d_6): δ 7.43 (s, 2H), 5.24-5.23 (m, 1H), 4.68 (s, 2H), 2.05-1.80 (m, 5H), 1.72-1.69 (m, overlapped, 1H), 1.69 (s, overlapped, 3H), 1.45-1.31 (m, overlapped, 1H), 1.41 (s, overlapped, 2H); ^{13}C NMR (101 MHz, DMSO-d_6): δ 149.7 (C), 135.6 (C), 118.4 (CH), 108.6 (CH_2), 40.6 (CH), 30.49 (CH_2), 30.45 (CH_2), 27.7 (CH_2), 26.2 (br s, CH_2), 20.7 (CH_3); ^{11}B NMR (128 MHz, DMSO-d_6): δ 31.1 (br s).

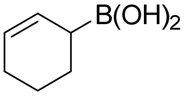

Cyclohex-2-enylboronic acid (4l) was prepared according to the general procedure from **3l** (2.0 mmol), **2b** (0.1 mmol) and **1** (2.4 mmol) in DMSO/H_2O (1.8 mL/0.2 mL) using aqueous NaCl 16% as the precipitant (8 mL) and washing with H_2O (2x2 mL). The product was obtained as a white fluffy solid (64 mg, 25% yield, 97% purity), containing a trace amount of H_3BO_3 (1%), H_2O (1%) and DMSO (1%). ^{1}H NMR (400 MHz, DMSO-d_6): δ 7.42 (s, 2H), 5.74-5.71 (m, 1H), 5.52-5.47 (m, 1H), 1.90 (br s, 2H), 1.67-1.53 (m, 4H), 1.50-1.39 (m, 1H); ^{13}C NMR (101 MHz, DMSO-d_6): δ 130.0 (CH), 124.0 (CH), 25.2 (br s, CH), 24.7 (CH_2), 24.1 (CH_2), 22.3 (CH_2); ^{11}B NMR (128 MHz, DMSO-d_6): δ 31.2 (br s).

General procedure for the synthesis of allyl boronic acids 4j-k (Table 1). To a solution of the allylic alcohol in the solvent mixture shown in Table 1, the palladium catalyst (**2a**, 5 mol%) and diboronic acid (**1**, 1.2 equivalents) were successively added and the mixture was stirred vigorously. From time to time aliquots from the reaction mixture were dissolved into $CDCl_3$ or DMSO-d_6 and analized by ^{1}H NMR. After the allotted times in Table 1 the conversion was 95-100%. Then (after 1-2 hours) the mixture was filtered through a HPLC Teflon filter (0.2 µm) into an Ar filled Schlenk tube containing a magnetic stir bar. Subsequently, degassed $CHCl_3$

(two times the reaction volume) was added, then the mixture was vigorously stirred with degassed NaCl 16% aq. solution (two times the reaction volume) and the aqueous layer was carefully removed. The washing was done five times. Finally a precisely weighed amount of naphtalene (10-20 mg) was added (as internal standard) and a 0.02 mL aliquot was taken into $CDCl_3$ (0.5 mL) for the determination of the yield. The boronic acid solution was used immediately for allylation of ketones.

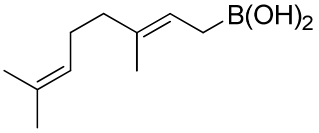

(E)-3,7-Dimethylocta-2,6-dienylboronic acid (4j) was prepared according to the general procedure from **3j** (1.0 mmol), **2a** (170 µL) and **1** (1.2 mmol) in DMSO/H_2O (1.6 mL/0.4 mL) using $CHCl_3$ (4 mL) and washing with aqueous NaCl 16% (5x4 mL); yield: 78%. If non-degassed solvents were used the yield of **4j** was decreased from 78% to 55%. Accordingly, when in a separate experiment **4j** was prepared according to the general procedure from **3j** (2.0 mmol), **2a** (330 µL) and **1** (2.4 mmol) in DMSO/H_2O (3.2 mL/0.8 mL) using *non-degassed* $CHCl_3$ (8 mL) and washing with *non-degassed* aqueous NaCl 16% (5x8 mL) *under air*; yield: 55%. ^{1}H NMR (500 MHz, $CHCl_3$/$CDCl_3$): δ 5.26 (triple hexuplet, J = 8.0, 1.3 Hz, 1H), 5.06 (triple heptuplet, J = 6.8, 1.4 Hz, 1H), 4.48 (s, B(OH)$_2$), 2.15-2.11 (m, 2H), 2.09-2.06 (m, 2H), 1.70 (m, 3H), 1.67 (d, J = 8.3 Hz, 2H), 1.61 (m, 3H), 1.56 (m, 3H); ^{13}C NMR (126 MHz, $CHCl_3$/$CDCl_3$): δ 136.6 (C), 132.5 (C), 124.4 (CH), 120.6 (CH), 39.7 (CH_2), 26.5 (CH_2), 25.9 (CH_3), 17.8 (CH_3), 15.7 (CH_3); the signal for CH$_2$B(OH)$_2$ could not be observed.

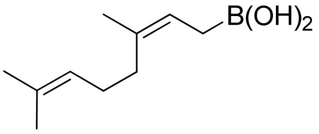

(Z)-3,7-Dimethylocta-2,6-dienylboronic acid (4k) was prepared according to the general procedure from **3k** (1.0 mmol), **2a** (170 µL) and **1** (1.2 mmol) in DMSO/H_2O (1.6 mL/0.4 mL) using $CHCl_3$ (4 mL) and washing with aqueous NaCl 16% (5x4 mL); yield: 79%. ^{1}H NMR (500 MHz, $CHCl_3$/$CDCl_3$): δ 5.27-5.24 (m, 1H), 5.10 (triple heptuplet, J = 6.9, 1.4 Hz, 1H), 4.39 (s, B(OH)$_2$), 2.10-2.05 (m, 2H), 2.04-2.01 (m, 2H), 1.73 (q, J = 1.2 Hz, 3H), 1.69-1.68 (m, overlapped, 3H), 1.67 (d, overlapped, J = 8.4 Hz, 2H), 1.61 (m, 3H); ^{13}C NMR (126 MHz, $CHCl_3$/$CDCl_3$): δ 136.8 (C), 132.4 (C), 124.1 (CH), 120.4 (CH), 31.7 (CH_2), 26.3 (CH_2), 25.9 (CH_3), 23.5 (CH_3), 17.8 (CH_3); the signal for CH$_2$B(OH)$_2$ could not be observed.

General procedure for the synthesis of homoallylic alcohols (6a-d) using isolated boronic acids (Table 2). Allylboronic acid **4a** was dissolved in THF under Ar and then the corresponding ketone was added. The reaction mixture was stirred at the given temperatures, for the reaction times mentioned in Table 2. After the completion of the reaction water was added and then this mixture was extracted with TBME and the organic phase was dried over $MgSO_4$. The solvent was evaporated and the product was purified by flash chromatography.

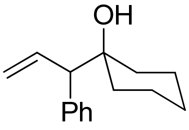

1-(1-Phenylallyl)cyclohexanol (6a) was prepared according to the general procedure from **4a** (0.2 mmol) and **5a** (0.22 mmol) in THF (0.4 mL) followed by flash chromatography (DCM). The product was obtained as colorless oil (37 mg, 86% yield). The NMR data are in agreement with literature values.[3] ^{1}H NMR (400 MHz, $CDCl_3$): δ 7.33-7.21 (m, 5H), 6.31(dt, J = 17.0, 9.9 Hz, 1H), 5.16 (dd, J = 10.2, 1.8 Hz, 1H), 5.14-5.09 (m, 1H), 3.25 (d, J = 9.6, 1H), 1.68-1.13 (m, 11H); ^{13}C NMR (101 MHz, $CDCl_3$): δ 141.1, 137.8, 129.4, 128.4, 126.7, 117.5, 72.8, 61.2, 36.0, 35.7, 25.8, 22.0; HRMS-ESI m/z: Calcd. For $C_{15}H_{20}ONa$ $[M+Na]^+$ 239.1406. Found 239.1397.

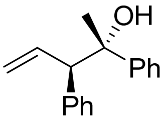

(2*S,3*R**)-2,3-diphenylpent-4-en-2-ol (6b)** was prepared according to the general procedure from **4a** (0.24 mmol) and **5b** (0.2 mmol) in THF (0.4 mL) followed by flash chromatography (pentane/Et_2O = 100:10). The product was obtained as colorless oil (45 mg, 89% yield). The NMR data are in agreement with literature values.[4,5] ^{1}H NMR (400 MHz, $CDCl_3$): δ 7.37-7.22 (m, 8H), 7.16-7.13 (m, 2H), 6.14 (ddd, J = 18.0, 9.4, 7.7 Hz, 1H), 5.07 (ddd, J = 10.3, 1.7, 0.8 Hz, 1H), 4.95 (ddd, J = 17.1, 1.7, 1.2 Hz, 1H), 3.65 (d, J = 8.6 Hz, 1H), 1.99 (br s, 1H, OH), 1.46 (s, 3H); ^{13}C NMR (101 MHz, $CDCl_3$): δ 146.6, 140.3, 137.5, 129.8, 128.2, 127.9, 127.0, 126.7, 125.7, 118.2, 76.4, 62.0, 28.7; HRMS-ESI m/z: Calcd for $C_{17}H_{18}ONa$ $[M+Na]^+$ 261.1250. Found 261.1247.

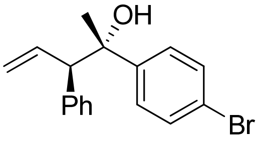

(2*S,3*R**)-2-(4-Bromophenyl)-3-phenylpent-4-en-2-ol (6c)** was prepared according to the general procedure from **4a** (.024 mmol) and **5c** (0.2 mmol) in THF (0.4 mL) followed by flash chromatography (pentane/Et_2O = 100:10). The product was obtained as colorless oil (58 mg, 91% yield). The NMR data are in agreement with literature values.[4] ^{1}H

NMR (500 MHz, $CDCl_3$): δ 7.43-7.40 (m, 2H), 7.30-7.19 (m, 5H), 7.13-7.11 (m, 2H), 6.11 (ddd, J = 18.1, 9.3, 7.8 Hz, 1H), 5.07 (ddd, J = 10.3, 1.7, 0.8 Hz, 1H), 4.96 (ddd, J = 17.1, 1.7, 1.1 Hz, 1H), 3.57 (d, J = 8.8 Hz, 1H), 1.98 (br s, 1H, OH), 1.43 (s, 3H); ^{13}C NMR (101 MHz, $CDCl_3$): δ 145.6, 139.9, 137.1, 131.0, 129.7, 128.4, 127.7, 127.1, 120.8, 118.6, 76.2, 62.0, 28.5; HRMS-ESI m/z: Calcd for $C_{17}H_{17}BrONa$ $[M+Na]^+$ 339.0355. Found 339.0348.

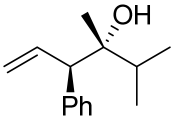

(3*S,4*S**)-2,3-Dimethyl-4-phenylhex-5-en-3-ol (6d)** was prepared according to the general procedure from **4a** (0.24 mmol) and **5d** (0.20 mmol) in THF (0.4 mL) followed by flash chromatography (pentane/Et_2O = 100:10). The product was obtained as colorless oil (37 mg, 90% yield). The NMR data are in agreement with literature values.[6] ^{1}H NMR (400 MHz, $CDCl_3$): δ 7.32-7.20 (m, 5H), 6.35 (dt, J = 17.1, 9.9 Hz, 1H), 5.15 (dd, J = 10.2, 1.8 Hz, 1H), 5.10 (dd, J = 17.1, 1.4 Hz, 1H), 3.43 (d, J = 9.6 Hz, 1H), 1.96 (hetp, J =8.6 Hz, 1H), 1.46 (br s, 1H, OH), 0.98 (d, J = 6.8 Hz, 3H), 0.92 (d, J = 6.8 Hz, 3H), 0.88 (s, 3H); ^{13}C NMR (101 MHz, $CDCl_3$): δ 142.0, 138.0, 129.5, 128.4, 126.6, 117.0, 76.2, 57.8, 34.2, 20.3, 17.7, 17.0; HRMS-ESI m/z: Calcd for $C_{14}H_{20}ONa$ $[M+Na]^+$ 227,1406. Found 227.1396.

General procedure for the synthesis of homoallylic alcohols (6e-g) using $CHCl_3$ solutions of boronic acids 4j-k (Table 2). Over a solution of the boronic acid in $CHCl_3$ (prepared as shown above), the ketone and MS 4Å (approx. 25% v/v) were sequentially added. Solids (including MS 4Å) were added using an Ar countercurrent. The mixture was stirred at r.t. for 18 hours, when complete conversion of the ketone was observed by ^{1}H NMR. The solution was separated from the molecular sieves which were washed with Et_2O three times. The combined organic solutions were concentrated over Celite and the product was purified by flash chromatography.

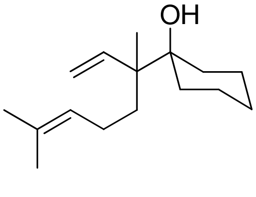

1-(3,7-Dimethylocta-1,6-dien-3-yl)cyclohexanol (6e) was prepared according to the general procedure from **5a** (0.55 mmol), **4j** (1.1 mmol)/$CHCl_3$ (approx. 8 mL) and MS 4Å (approx. 2 mL) followed by flash chromatography (pentane/Et_2O = 100:6). The product was obtained as a clear oil (125 mg, 96%

yield). The NMR data are in agreement with literature values.[7] ^{1}H NMR (400 MHz, $CDCl_3$): δ 5.86 (dd, J = 17.7, 10.9 Hz, 1H), 5.21 (dd, J = 10.9, 1.6 Hz, 1H), 5.10 (triple heptuplet, J = 7.0, 1.4 Hz, 1H), 5.02 (dd, J = 17.7, 6.7 Hz, 1H), 1.90-1.76 (m, 2H), 1.67 (m, overlapped, 3H), 1.67-1.33 (m, overlapped, 12 H), 1.58 (m, overlapped, 3H), 1.12-1.01 (m, overlapped, 1H), 1.01 (m, overlapped, 3H); ^{13}C NMR (101 MHz, $CDCl_3$): δ 144.0 (CH), 131.3 (C), 125.3 (CH), 115.5 (CH_2), 75.0 (C-OH), 47.8 (C), 34.9 (CH_2), 31.9 (CH_2), 31.2 (CH_2), 26.0 (CH_2), 25.8 (CH_3), 23.6 (CH_2), 22.2 (CH_2), 22.0 (CH_2), 17.7 (CH_3), 16.3 (CH_3).

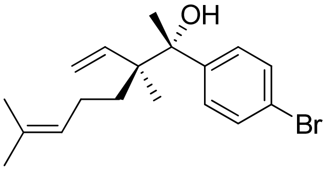

(2S*,3S*)-2-(4-Bromophenyl)-3,7-dimethyl-3-vinyloct-6-en-2-ol (6f) was prepared according to the general procedure from **5c** (0.39 mmol), **4j** (0.77 mmol)/$CHCl_3$ (approx. 4 mL) and MS 4Å (approx. 1 mL) followed by flash chromatography (pentane/Et_2O = 100:4 to 100:10). The product was obtained as a clear oil (124 mg, 94% yield, d.r. = 98:2). ^{1}H NMR (400 MHz, $CDCl_3$): δ 7.43-7.40 (m, 2H), 7.32-7.28 (m, 2H), 5.85 (dd, J = 17.6, 10.9 Hz, 1H), 5.28 (dd, J = 10.9, 1.4 Hz, 1H), 5.08 (dd, J = 17.6, 1.5 Hz, 1H), 5.02 (triple heptuplet, J = 7.1, 1.4 Hz, 1H), 1.94 (br s, 1H), 1.80-1.67 (m, 2H), 1.65 (m, 3H), 1.55 (s, 3H), 1.53 (m, 3H), 1.45-1.33 (m, 2H), 0.93 (s, 3H); ^{13}C NMR (101 MHz, $CDCl_3$): δ 144.2 (C), 143.3 (CH), 131.4 (C), 130.3 (CH), 129.4 (CH), 124.9 (CH), 120.8 (C), 116.7 (CH_2), 77.7 (C-OH), 48.0 (C), 35.7 (CH_2), 25.8 (CH_3), 25.7 (CH_3), 23.5 (CH_2), 17.8 (CH_3), 16.7 (CH_3); HRMS-ESI m/z: Calcd for $C_{18}H_{25}BrNaO^+$ $[M+Na]^+$ 359.0981. Found, 359.0966.

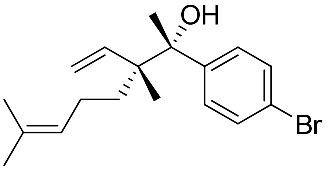

(2S*,3R*)-2-(4-Bromophenyl)-3,7-dimethyl-3-vinyloct-6-en-2-ol (6g) was prepared according to the general procedure from **5c** (0.40 mmol), **4k** (0.79 mmol)/$CHCl_3$ (approx. 4 mL) and MS 4Å (approx. 1 mL) followed by flash chromatography (pentane/Et_2O = 100:10). The chromatography had to be done 4 times in order to remove an impurity eluting close to the product. The product was obtained as a clear oil (103 mg, 76% yield, d.r. = 99:1). ^{1}H NMR (400 MHz, $CDCl_3$): δ 7.43-7.40 (m, 2H), 7.27-7.24 (m, 2H), 5.72 (dd, J = 17.7, 10.9 Hz, 1H), 5.25 (dd, J = 10.9, 1.4 Hz, 1H), 5.02 (dd, overlapped, J

= 17.7, 1.4 Hz, 1H), 5.01 (triple heptuplet, overlapped, *J* = 6.9, 1.4 Hz, 1H), 1.97 (br s, 1H), 1.80-1.67 (m, 2H), 1.65 (m, 3H), 1.54 (s, 3H), 1.52 (s, 3H), 1.50-1.43 (m, 1H), 1.30-1.22 (m, 1H), 1.00 (s, 3H); ^{13}C NMR (101 MHz, $CDCl_3$): δ 144.8 (C), 143.0 (CH), 131.4 (C), 130.3 (CH), 129.3 (CH), 125.0 (CH), 120.7 (C), 116.3 (CH_2), 77.9 (C-OH), 47.9 (C), 35.0 (CH_2), 25.8 (CH_3), 25.3 (CH_3), 23.4 (CH_2), 17.8 (CH_3), 17.2 (CH_3).

References:

(1) Fulmer, G. R.; Miller, A. J. M.; Sherden, N. H.; Gottlieb, H. E.; Nudelman, A.; Stoltz, B. M.; Bercaw, J. E.; Goldberg, K. I. *Organometallics* **2010**, *29*, 2176.

(2) Baber, R. A.; Norman, N. C.; Orpen, A. G.; Rossi, J. *New J. Chem.* **2003**, *27*, 773.

(3) Selander, N.; Kipke, A.; Sebelius, S.; Szabó, K. J. *J. Am. Chem. Soc.* **2007**, *129*, 13723.

(4) Yasuda, M.; Hirata, K.; Nishino, M.; Yamamoto, A.; Baba, A. *J. Am. Chem. Soc.* **2002**, *124*, 13442.

(5) Fandrick, K. R.; Fandrick, D. R.; Gao, J. J.; Reeves, J. T.; Tan, Z.; Li, W.; Song, J. J.; Lu, B.; Yee, N. K.; Senanayake, C. H. *Org. Lett.* **2010**, *12*, 3748.

(6) Ren, H.; Dunet, G.; Mayer, P.; Knochel, P. *J. Am. Chem. Soc.* **2007**, *129*, 5376.

(7) Araki, S.; Hatano, M.; Ito, H.; Butsugan, Y. *J Organomet. Chem.* **1987**, *333*, 329.

^{1}H NMR of **1** (99.5%) in DMSO-d_6 (0.5% H_3BO_3; 0.0% excess H_2O). The H_2O peak at 3.3 ppm arises from the equilibrium[2] given in page 2.

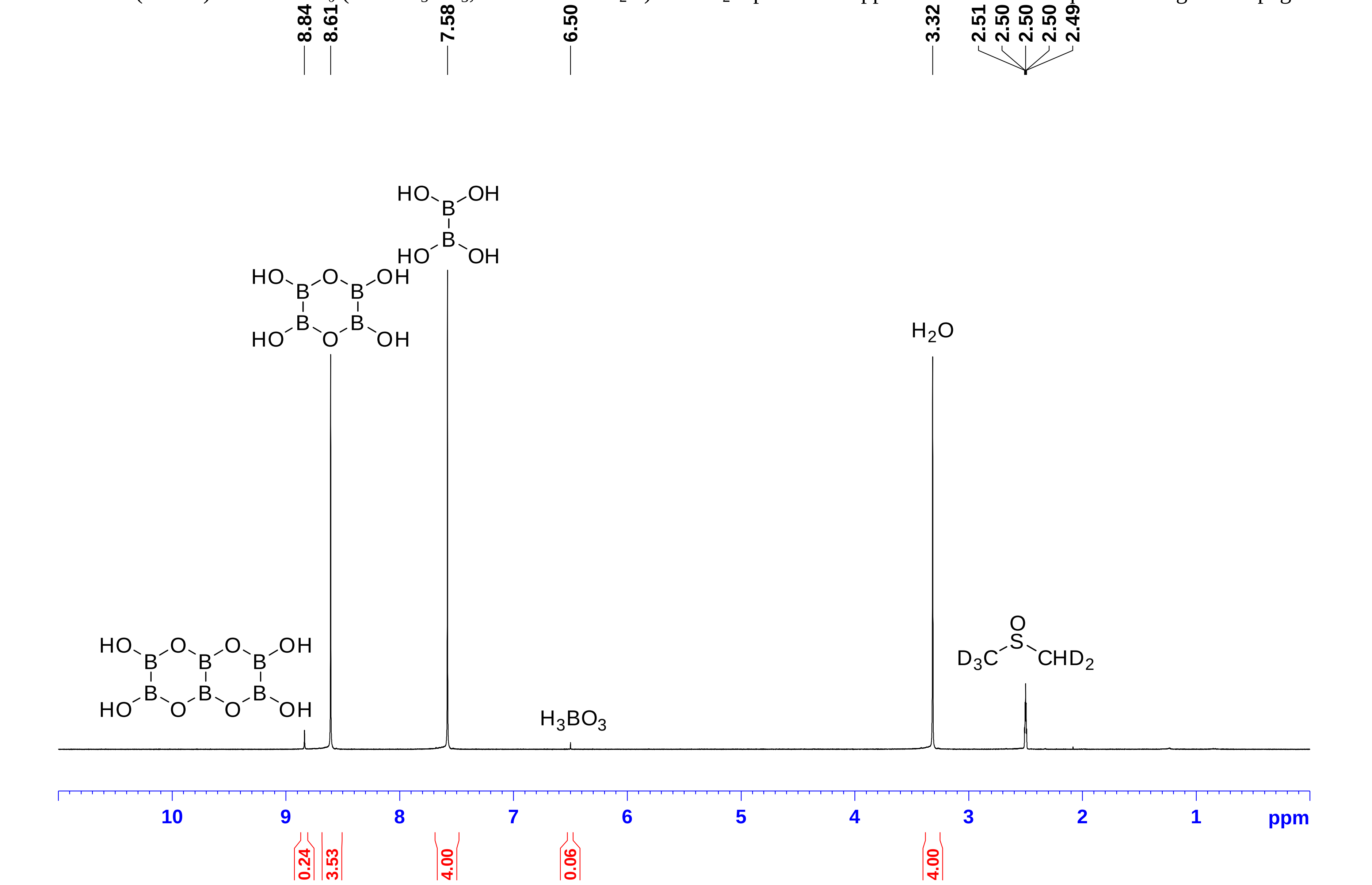

^{1}H NMR of **1** (99.5%) in DMSO-d_6 (blue), **1** (99.5%) in wet DMSO-d_6 (red), **1** (commercial grade) in DMSO-d_6 (green), **1** (commercial grade) in wet DMSO-d_6 (violet)

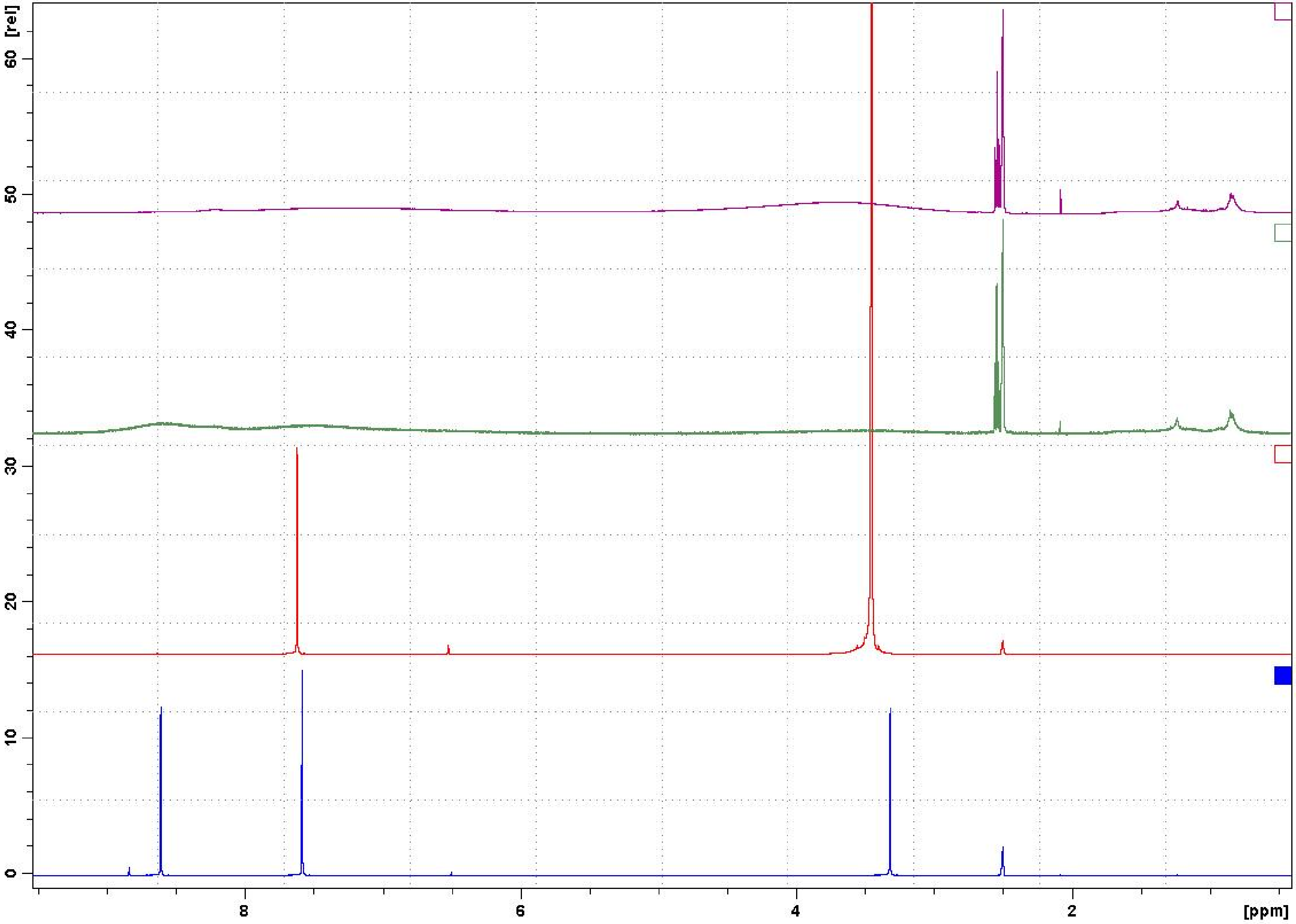

^{1}H NMR of **4a** in DMSO-d_6

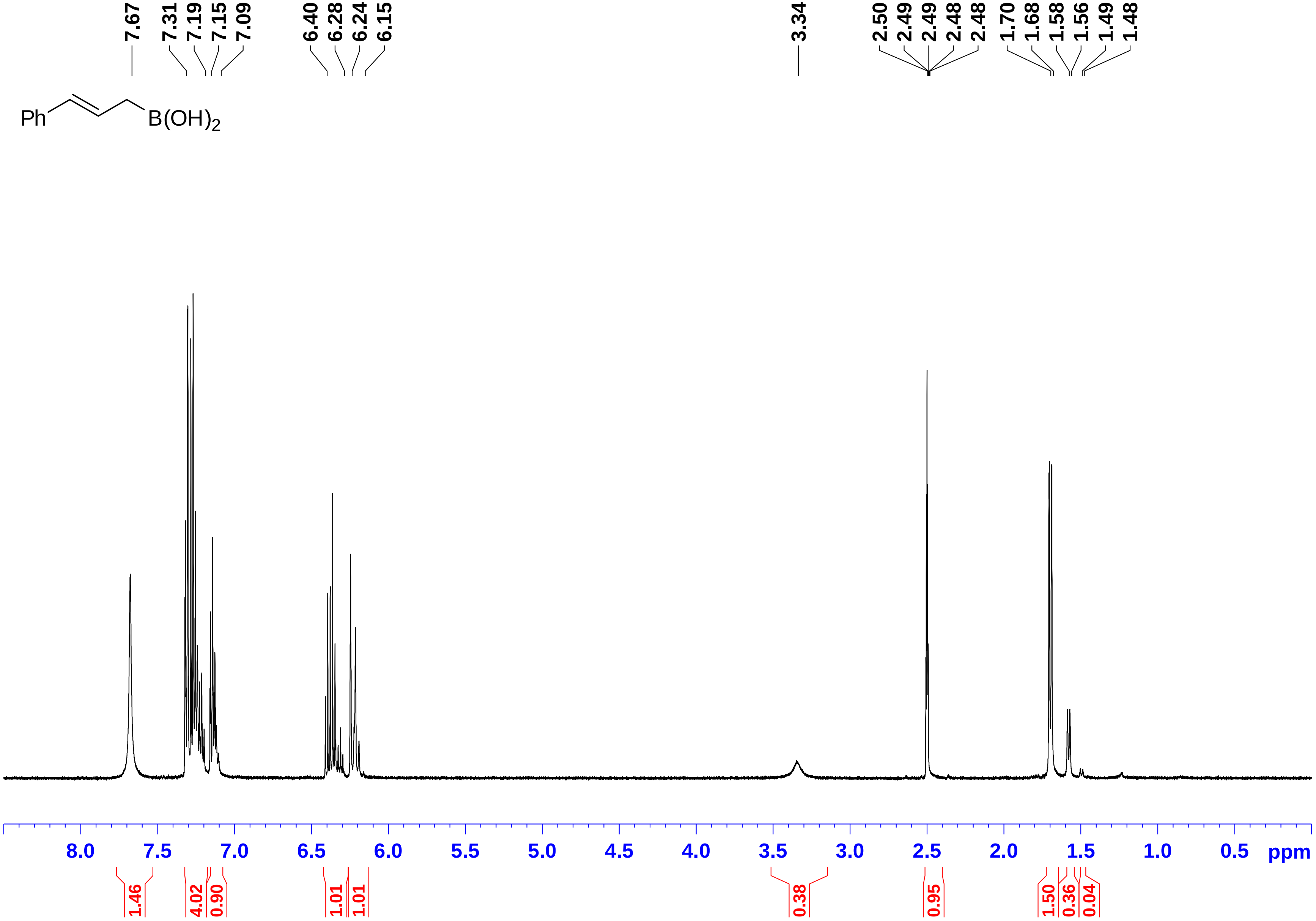

^{1}H NMR of **4a** in wet DMSO-d_6

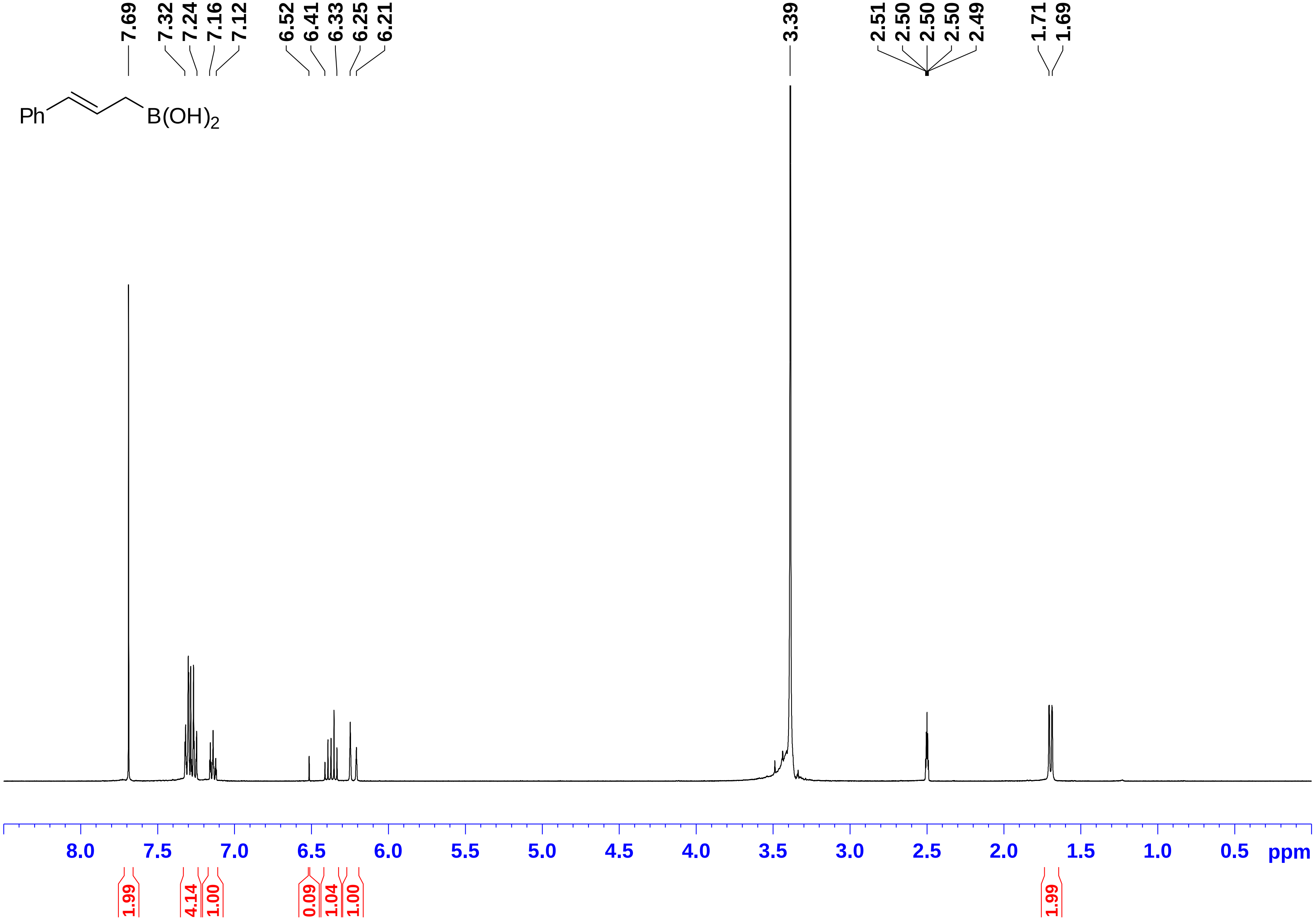

^{13}C NMR of **4a** in wet DMSO-d_6

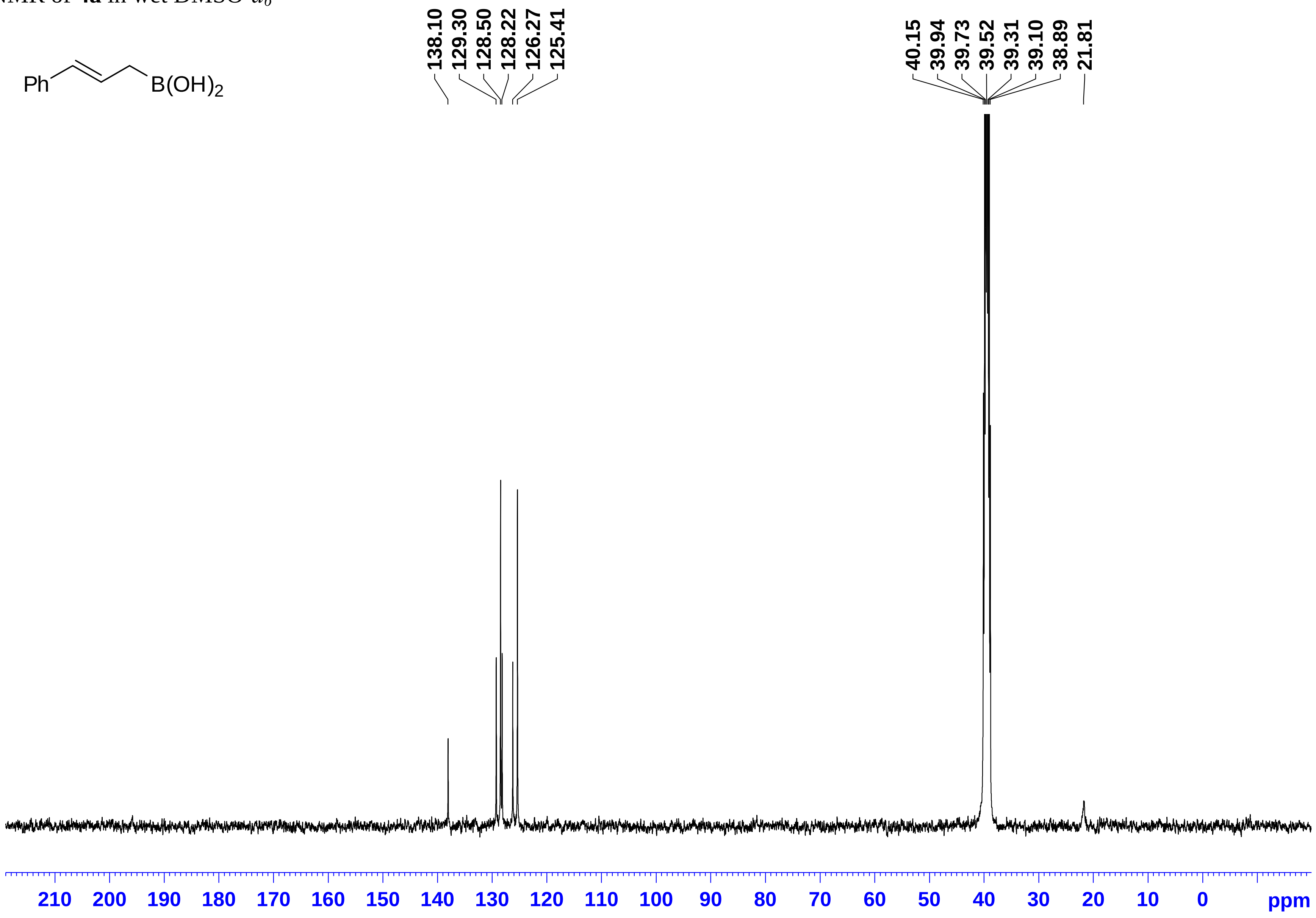

^{1}H NMR of **4b** in DMSO-d_6

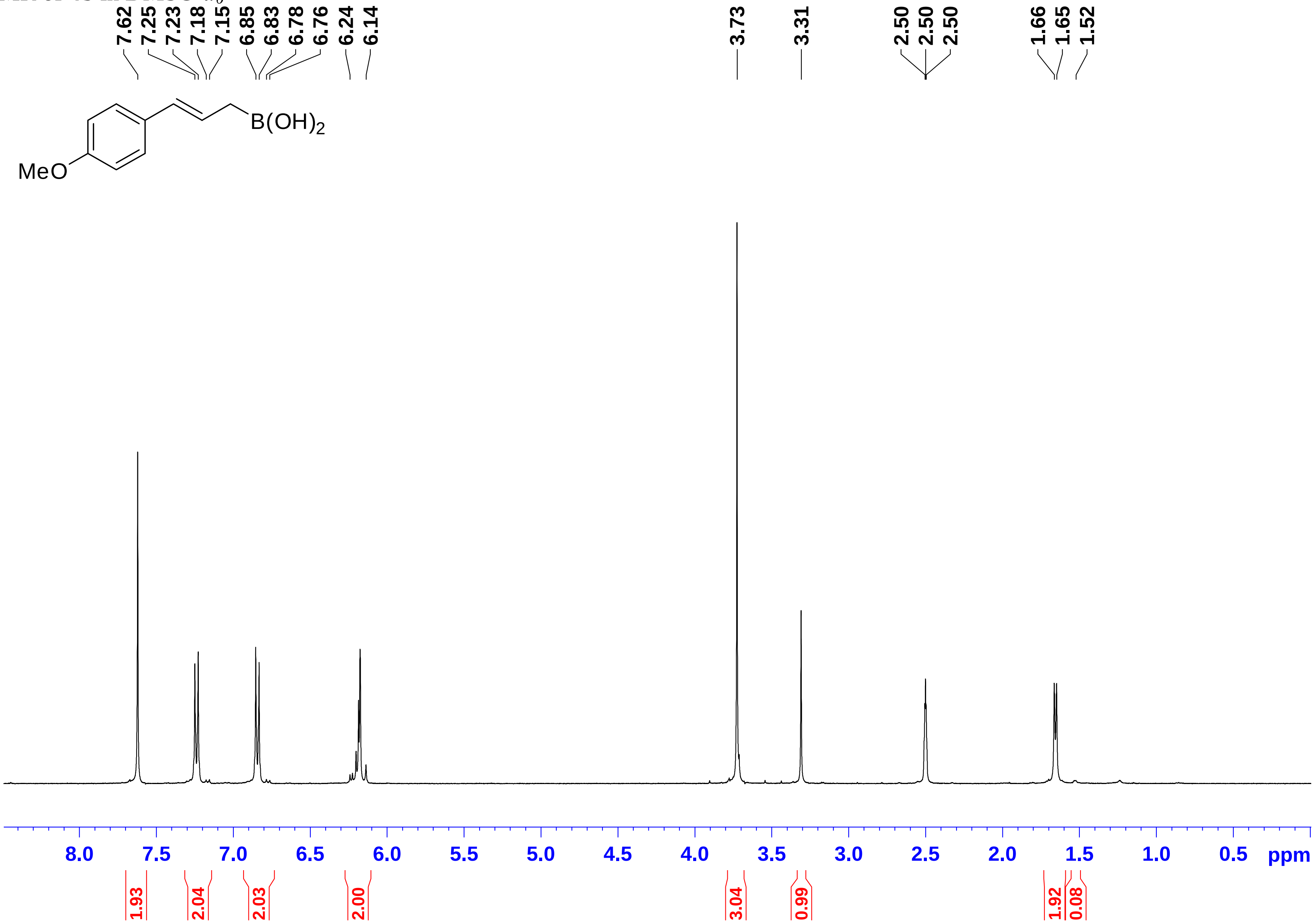

^{1}H NMR of **4b** in wet DMSO-d_6

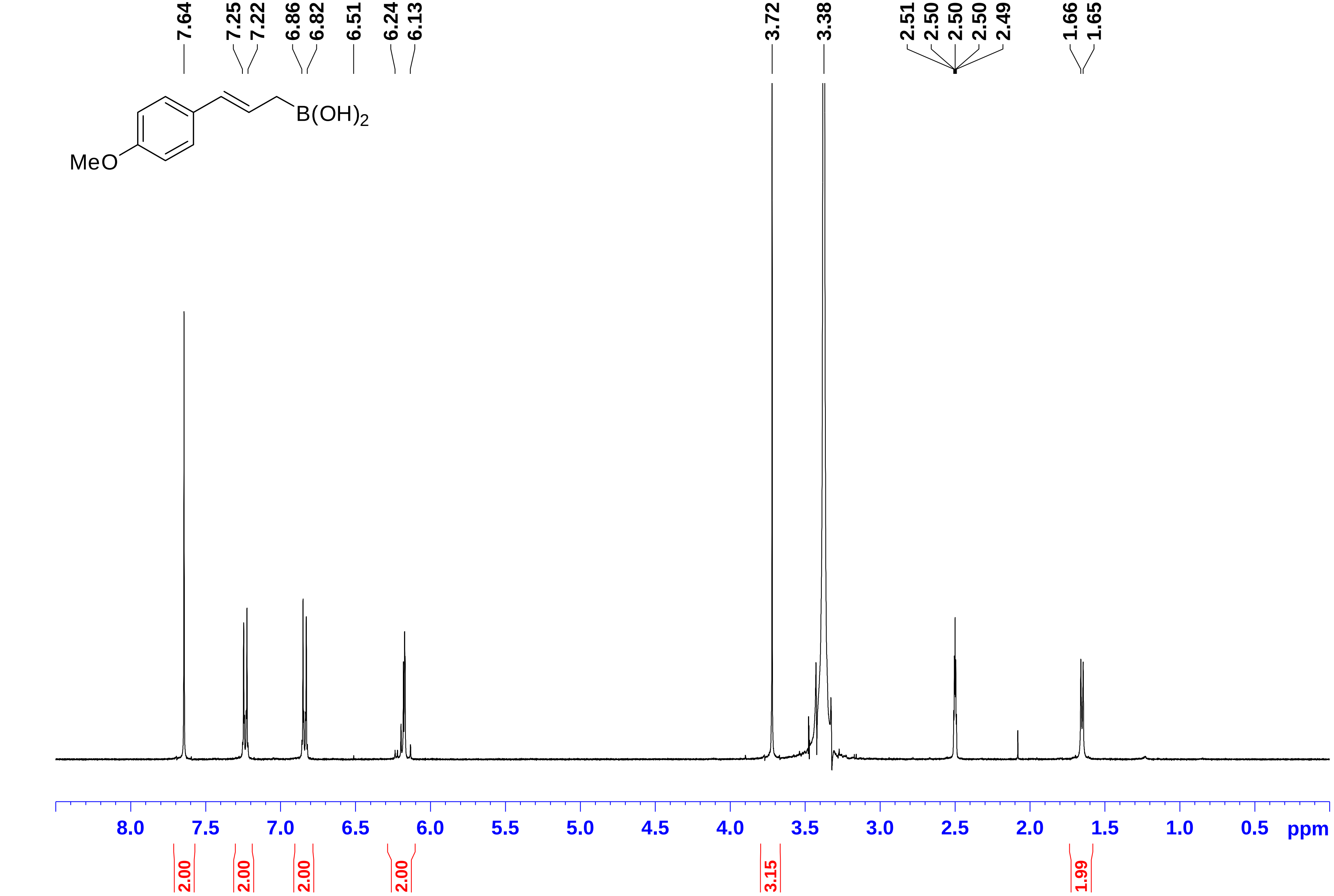

^{13}C NMR of **4b** in wet DMSO-d_6

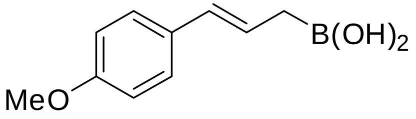

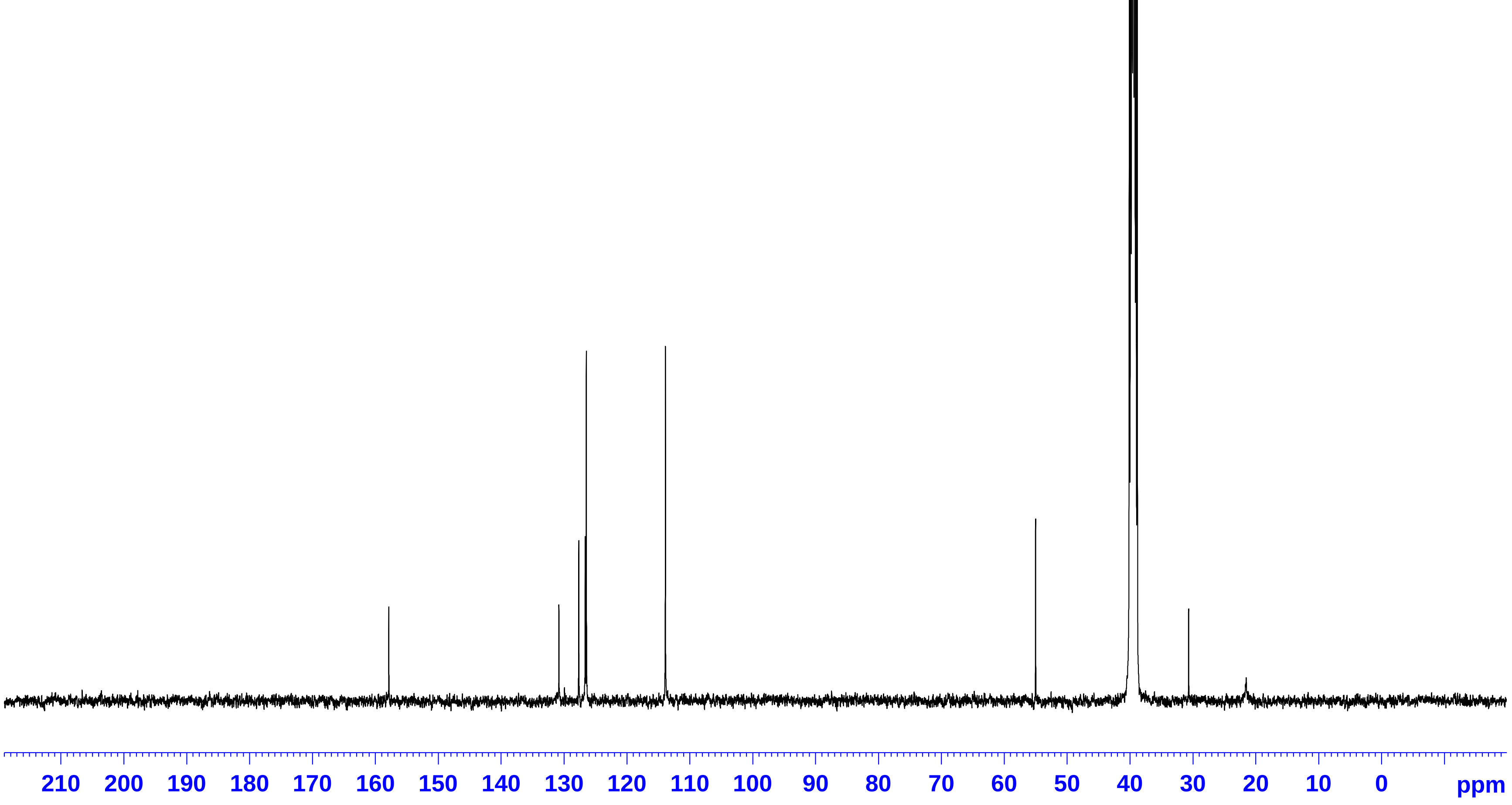

^{1}H NMR of **4c** in DMSO-d_6

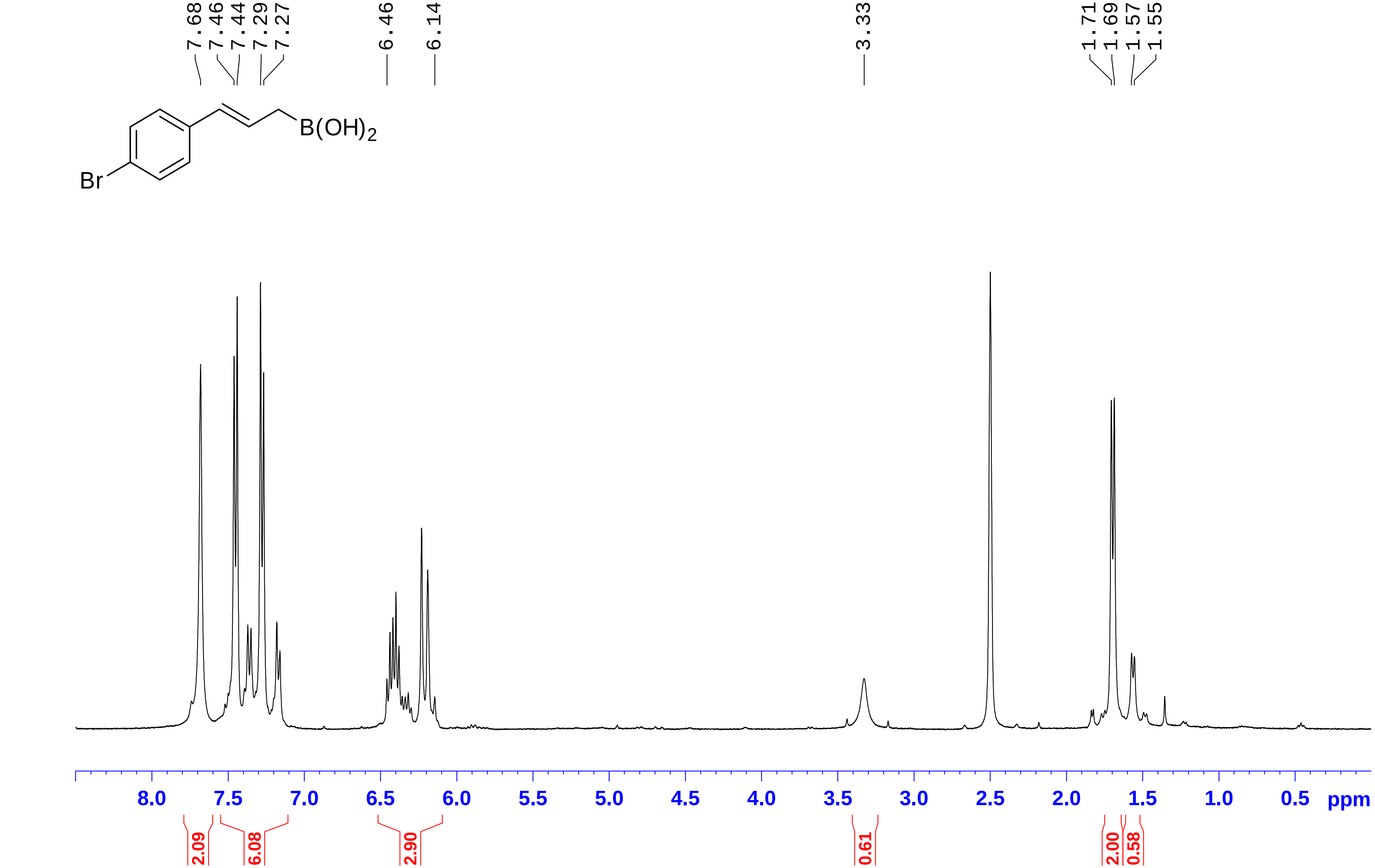

^{1}H NMR of **4c** in wet DMSO-d_6

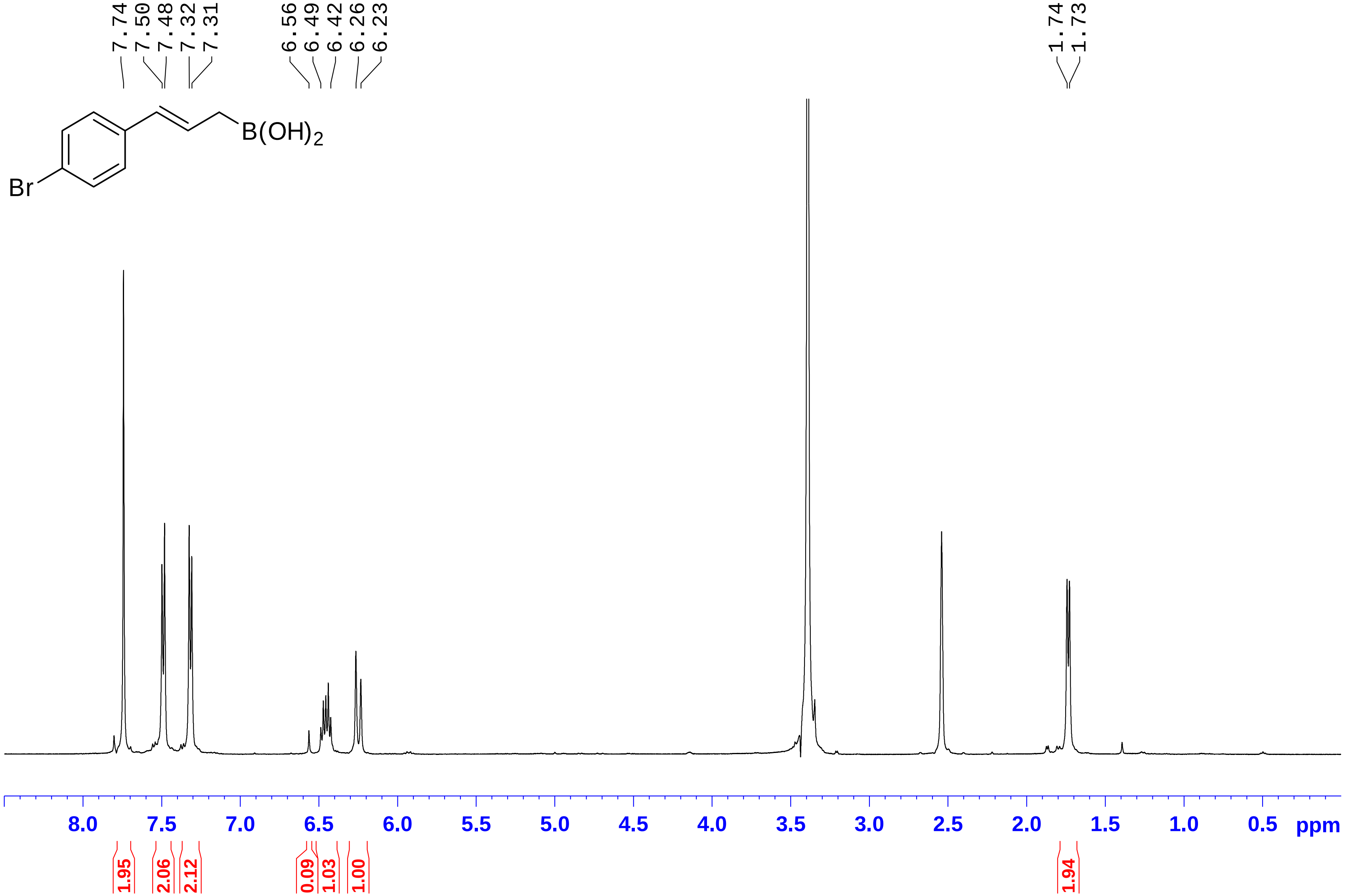

^{13}C NMR of **4c** in wet DMSO-d_6

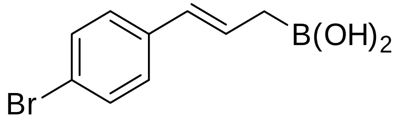

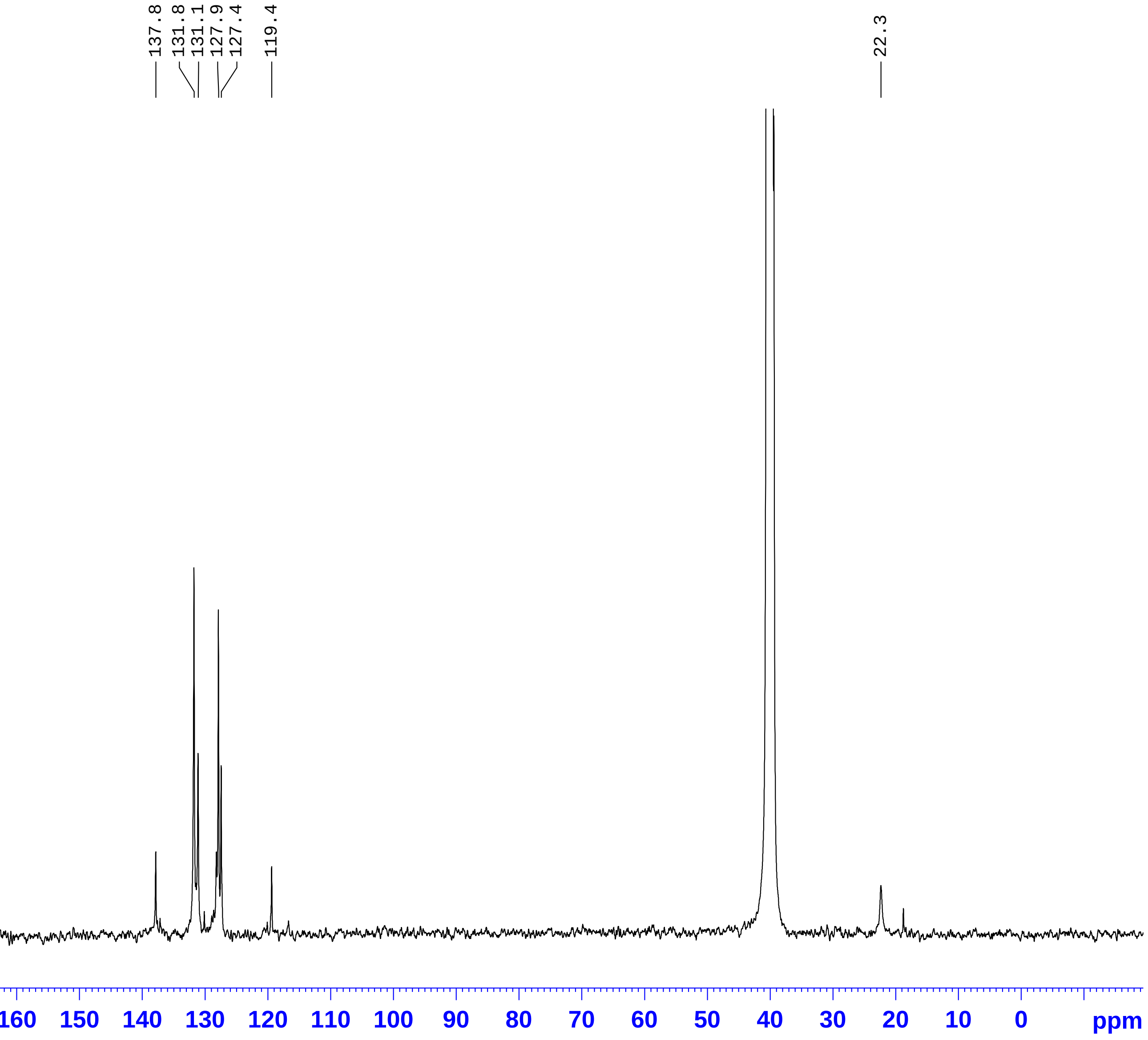

^{1}H NMR of **4d** in DMSO-d_6

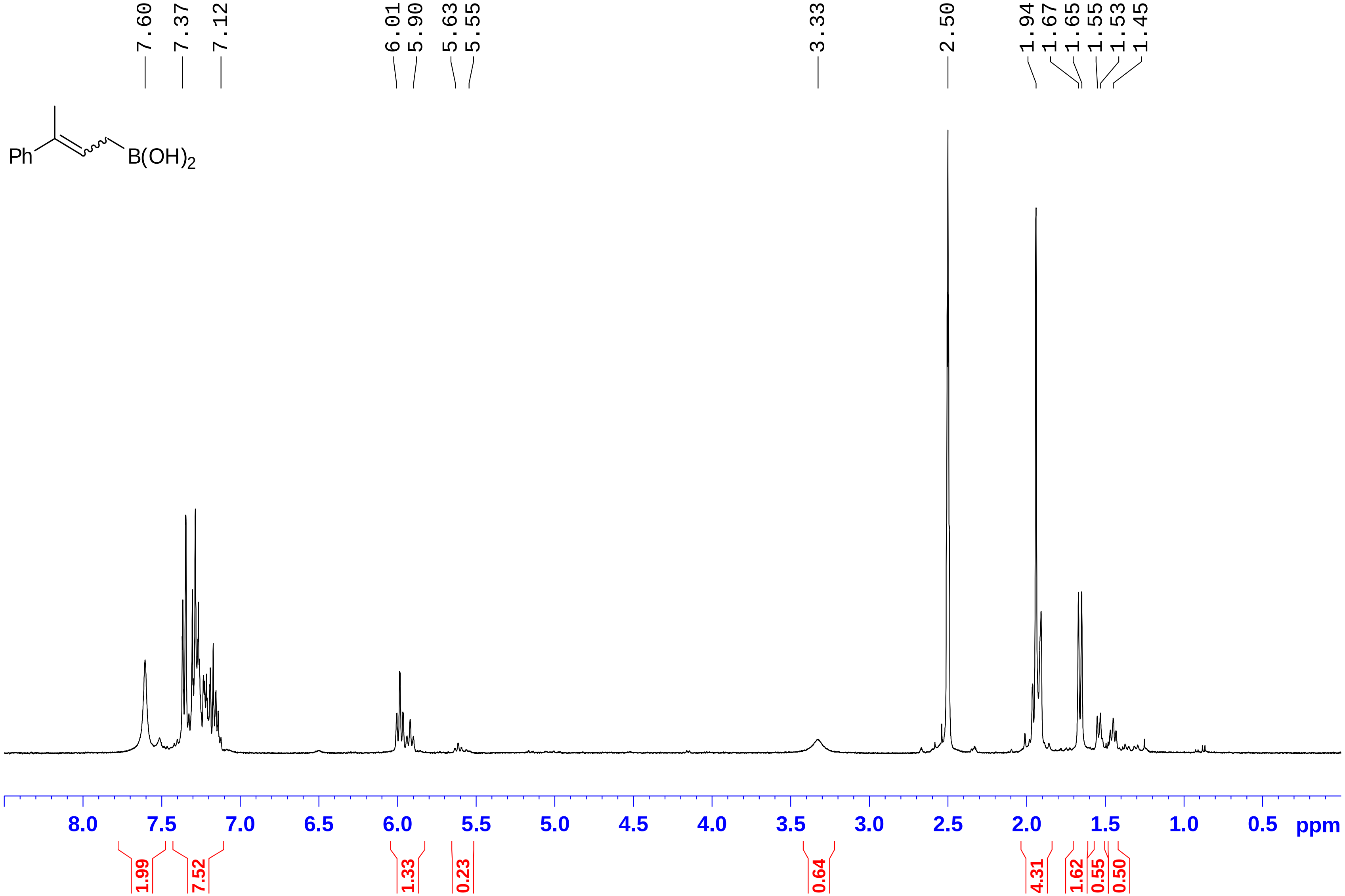

^{1}H NMR of **4d** in wet DMSO-d_6

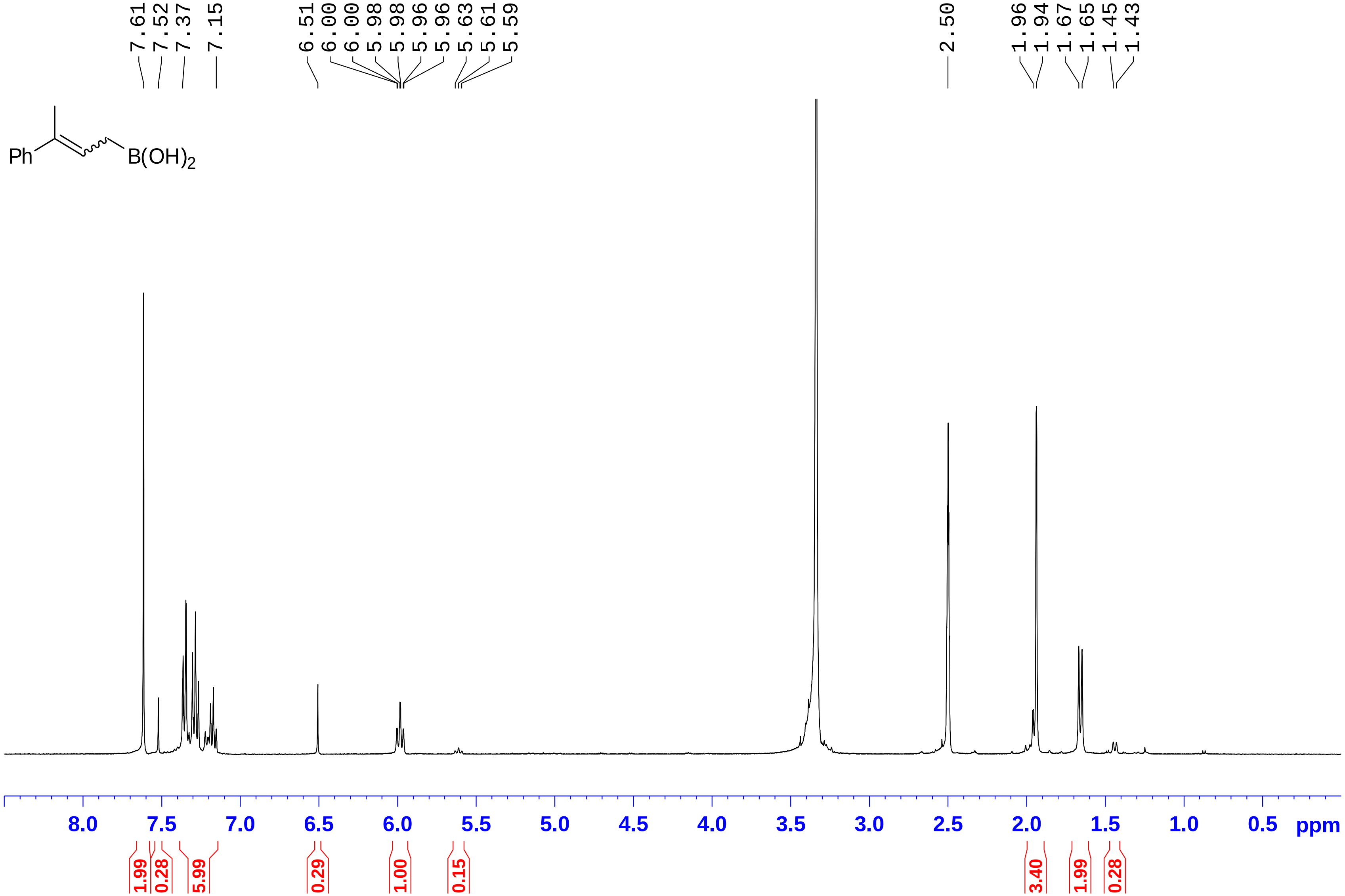

^{13}C NMR of **4d** in wet DMSO-d_6

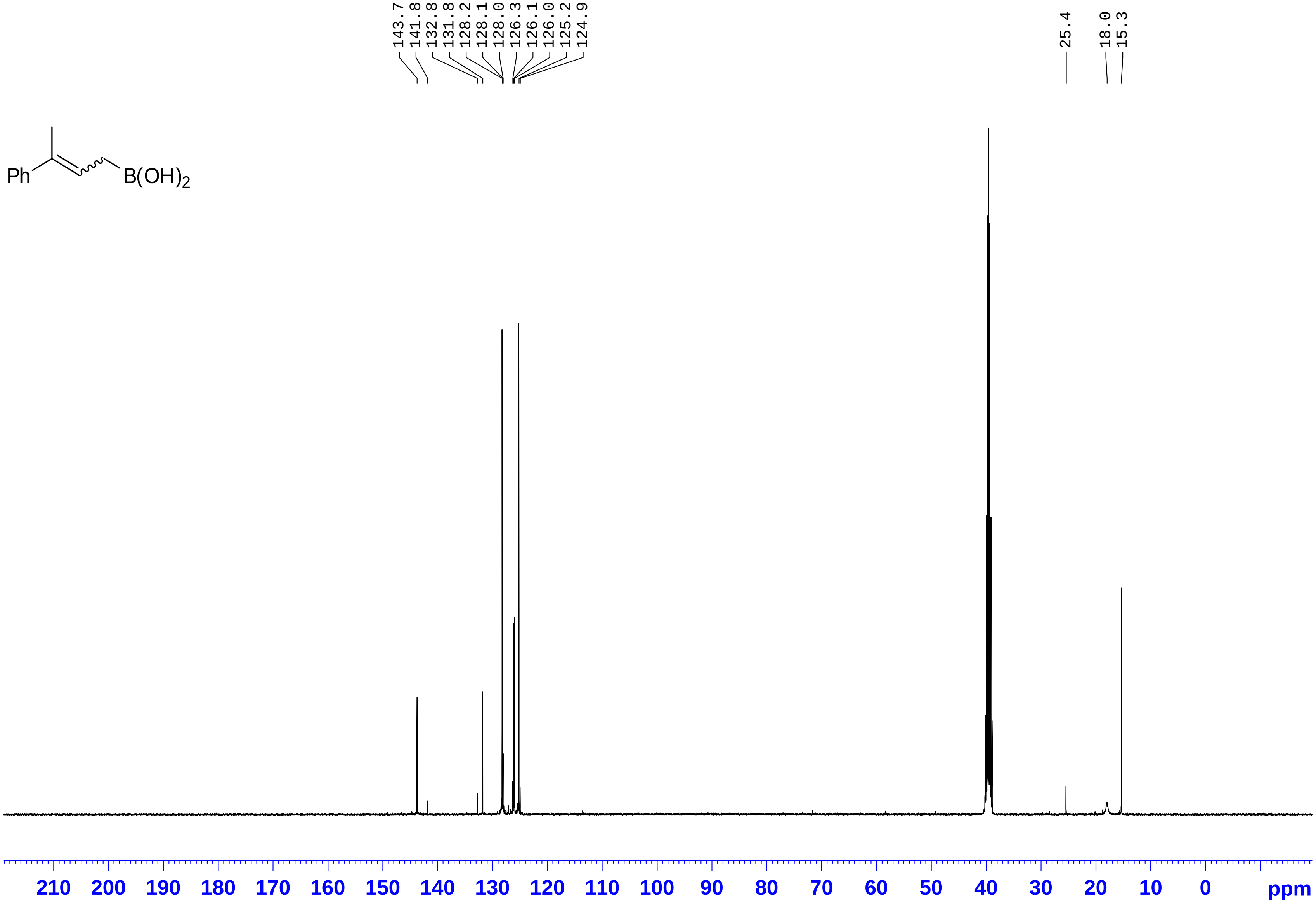

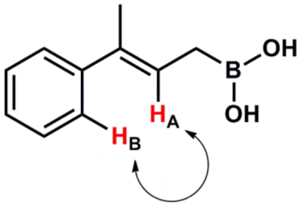

NOESY

4d_Determination of *E* selectivity

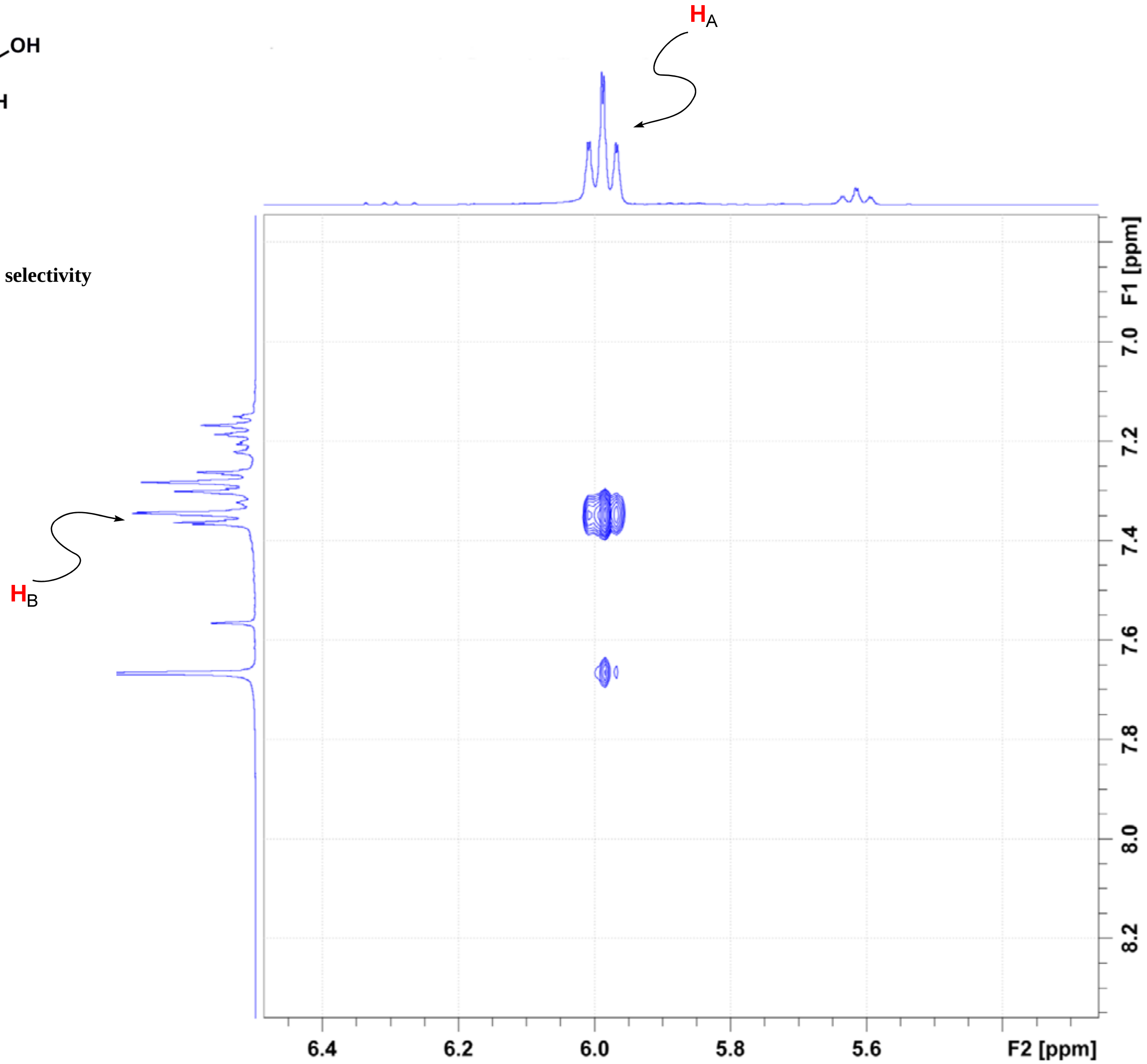

^{1}H NMR of **4e** in DMSO-d_6

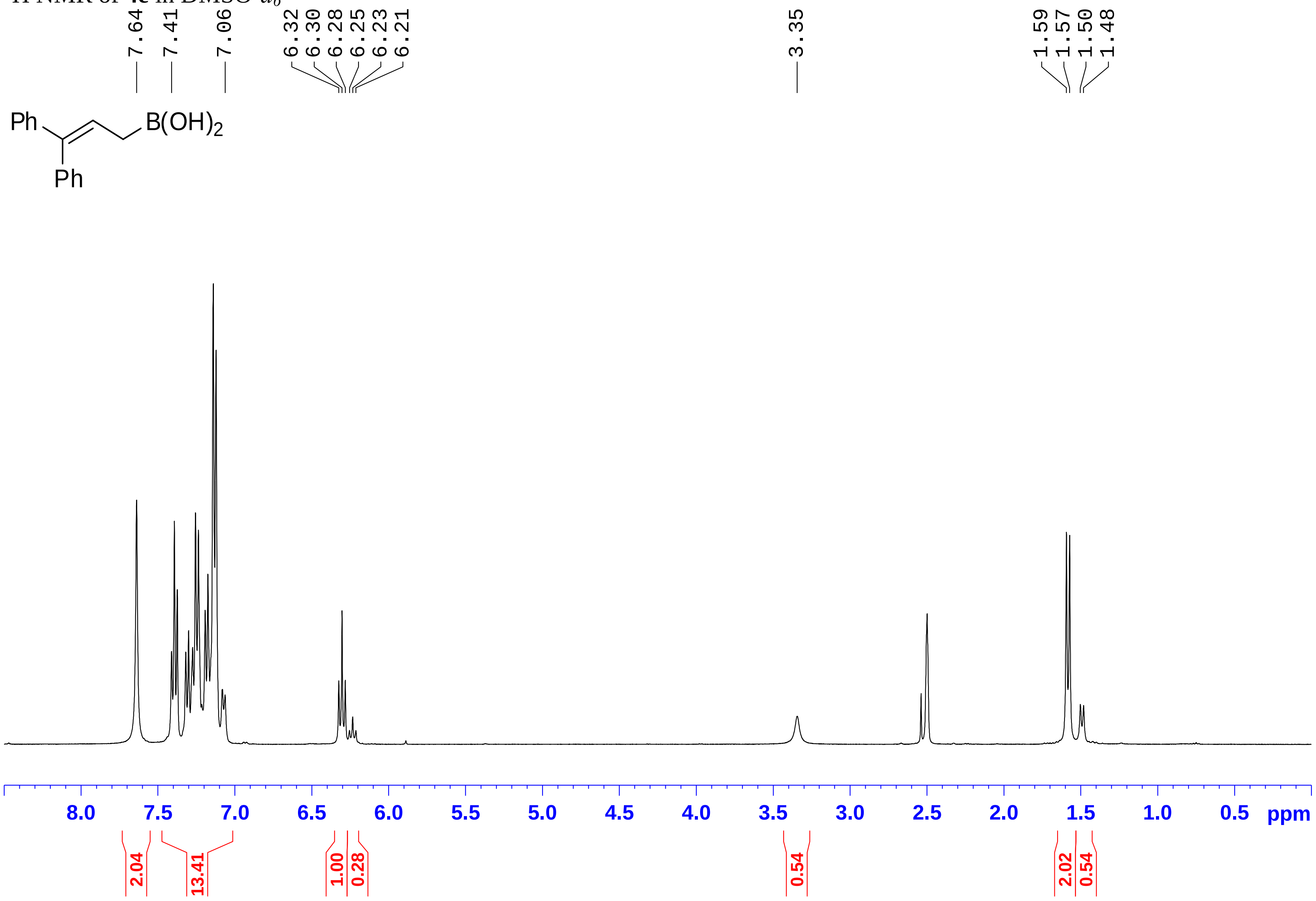

^{1}H NMR of **4e** in wet DMSO-d_6

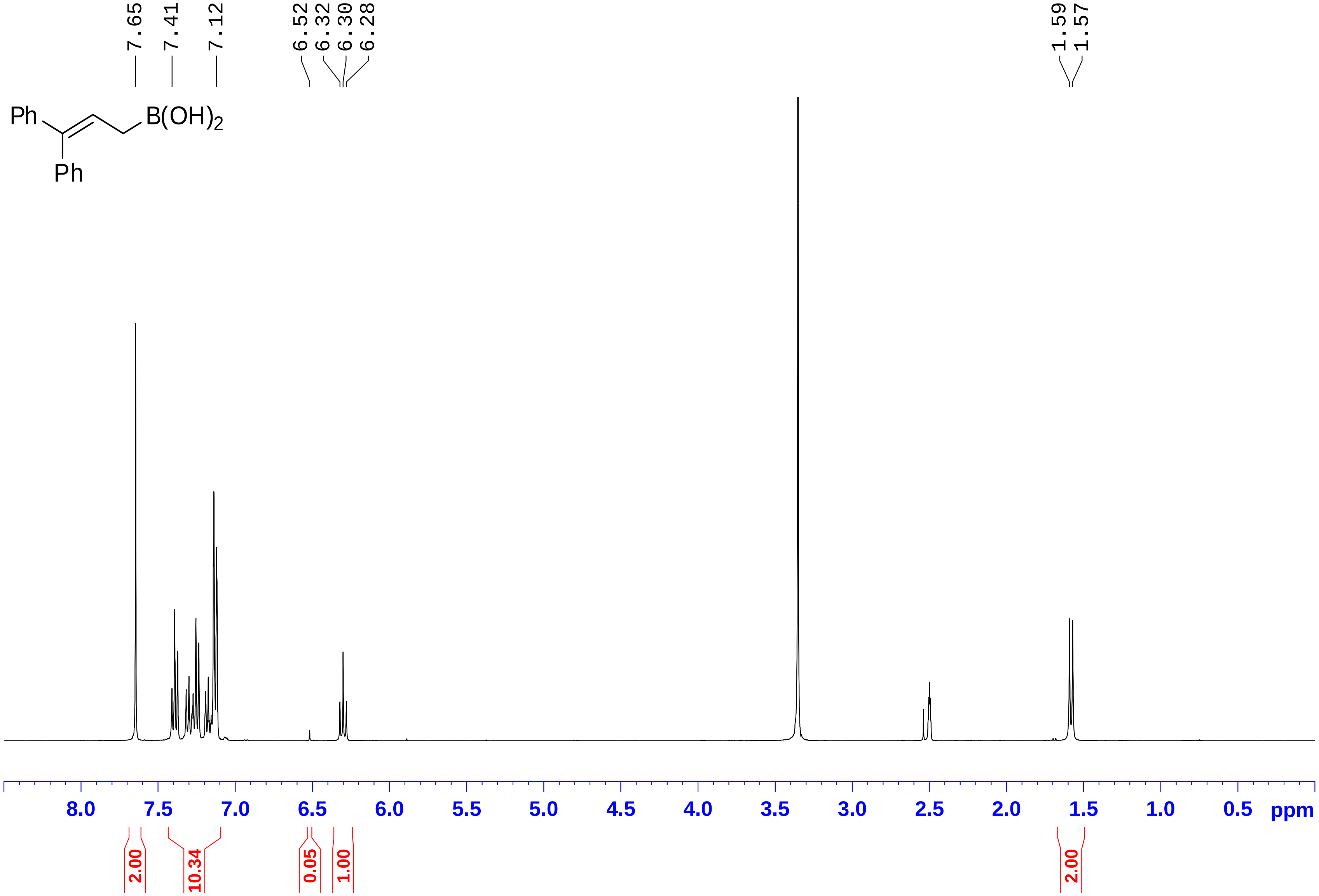

^{13}C NMR of **4e** in wet DMSO-d_6

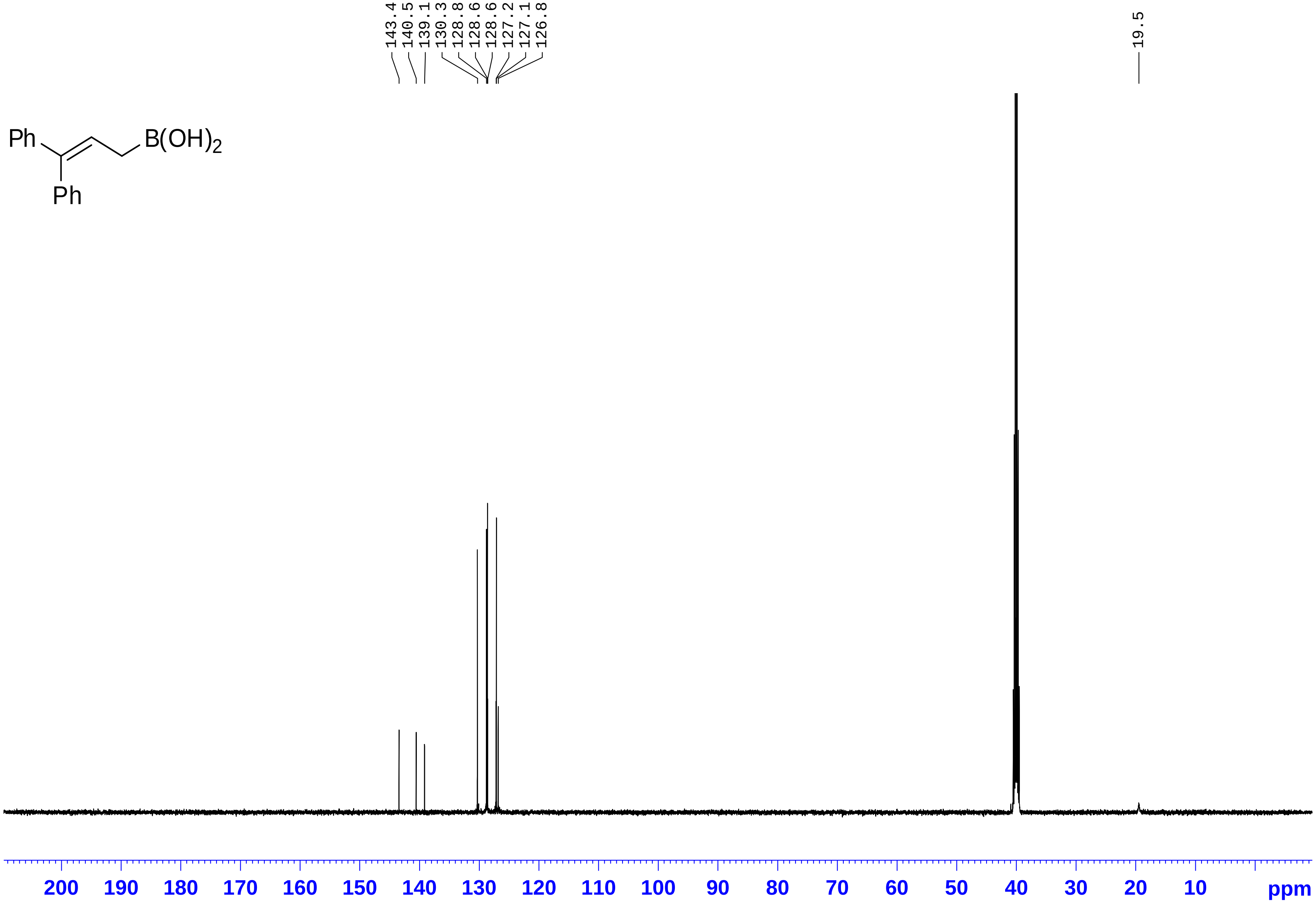

^{1}H NMR of **4f** in DMSO-d_6

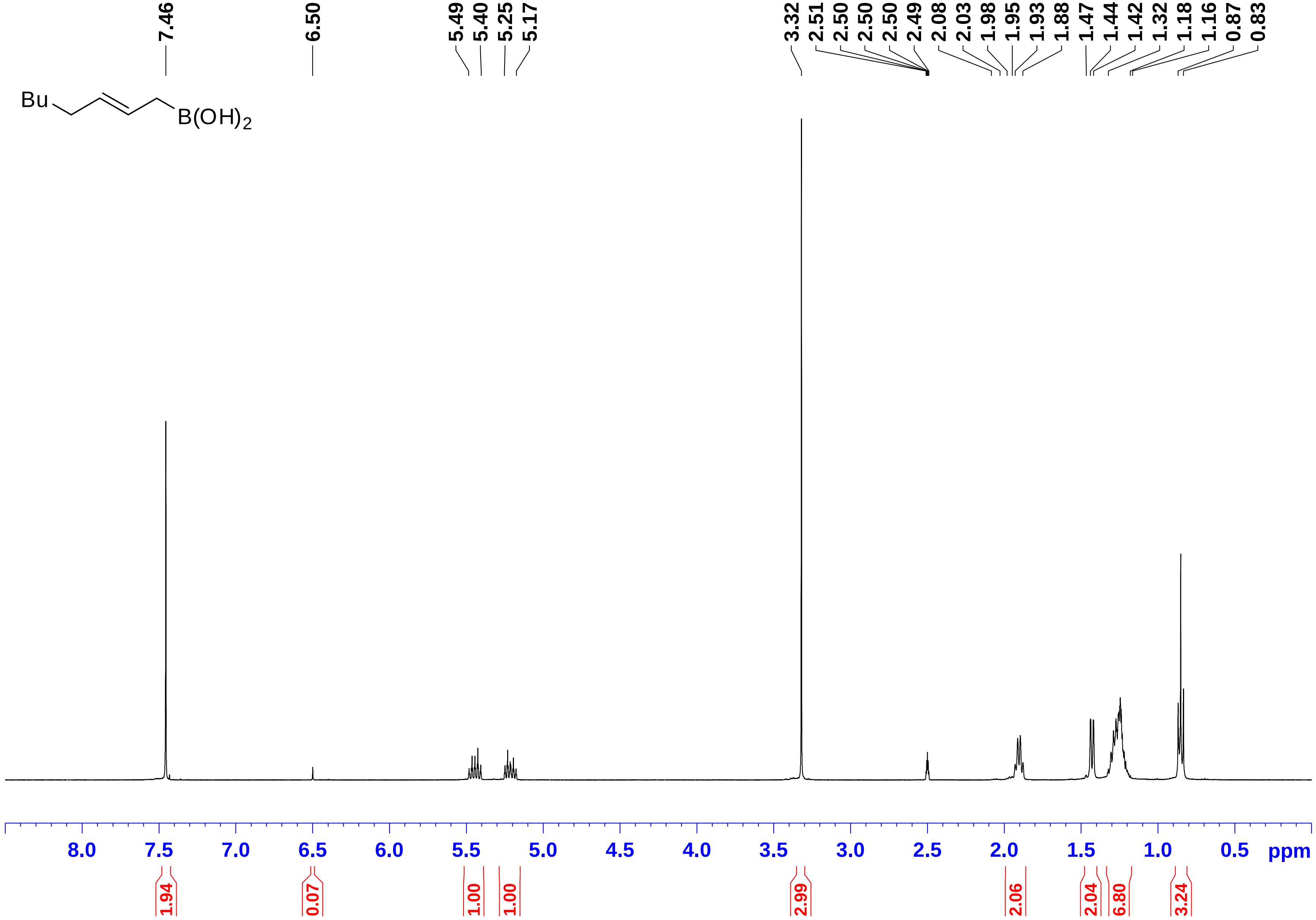

^{1}H NMR of **4f** in wet DMSO-d_6

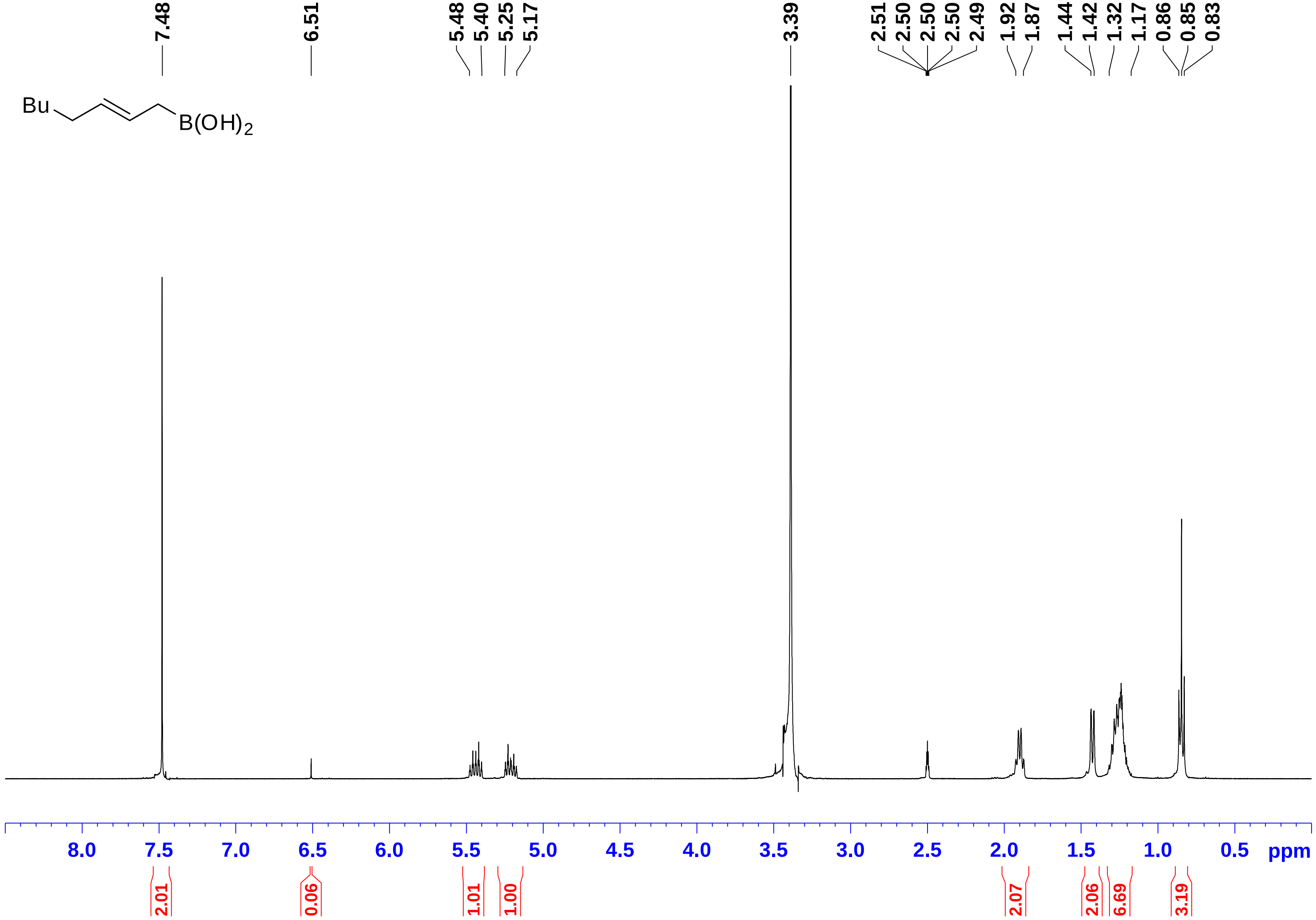

^{13}C NMR of **4f** in wet DMSO-d_6

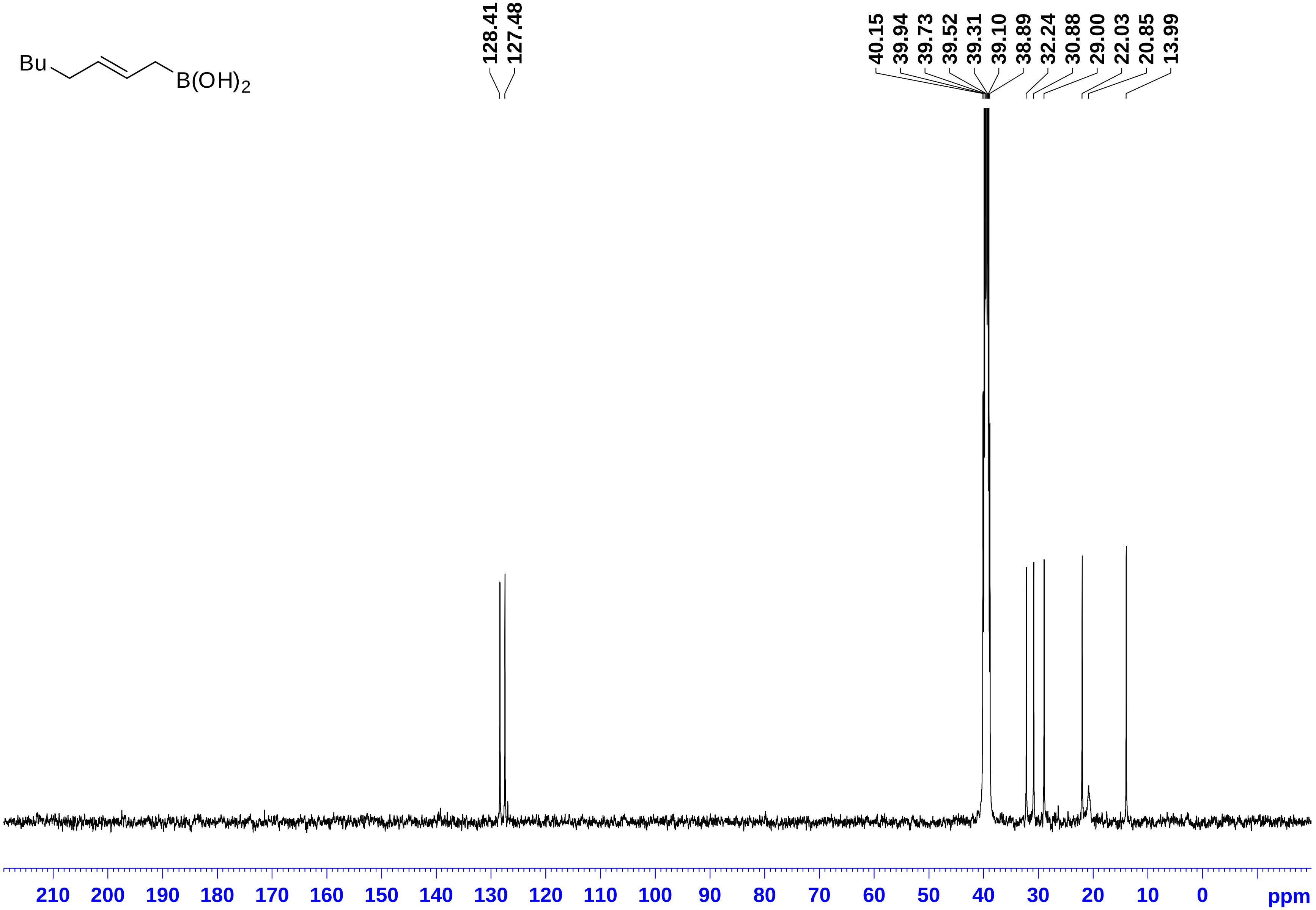

^{1}H NMR of **4g** in DMSO-d_6

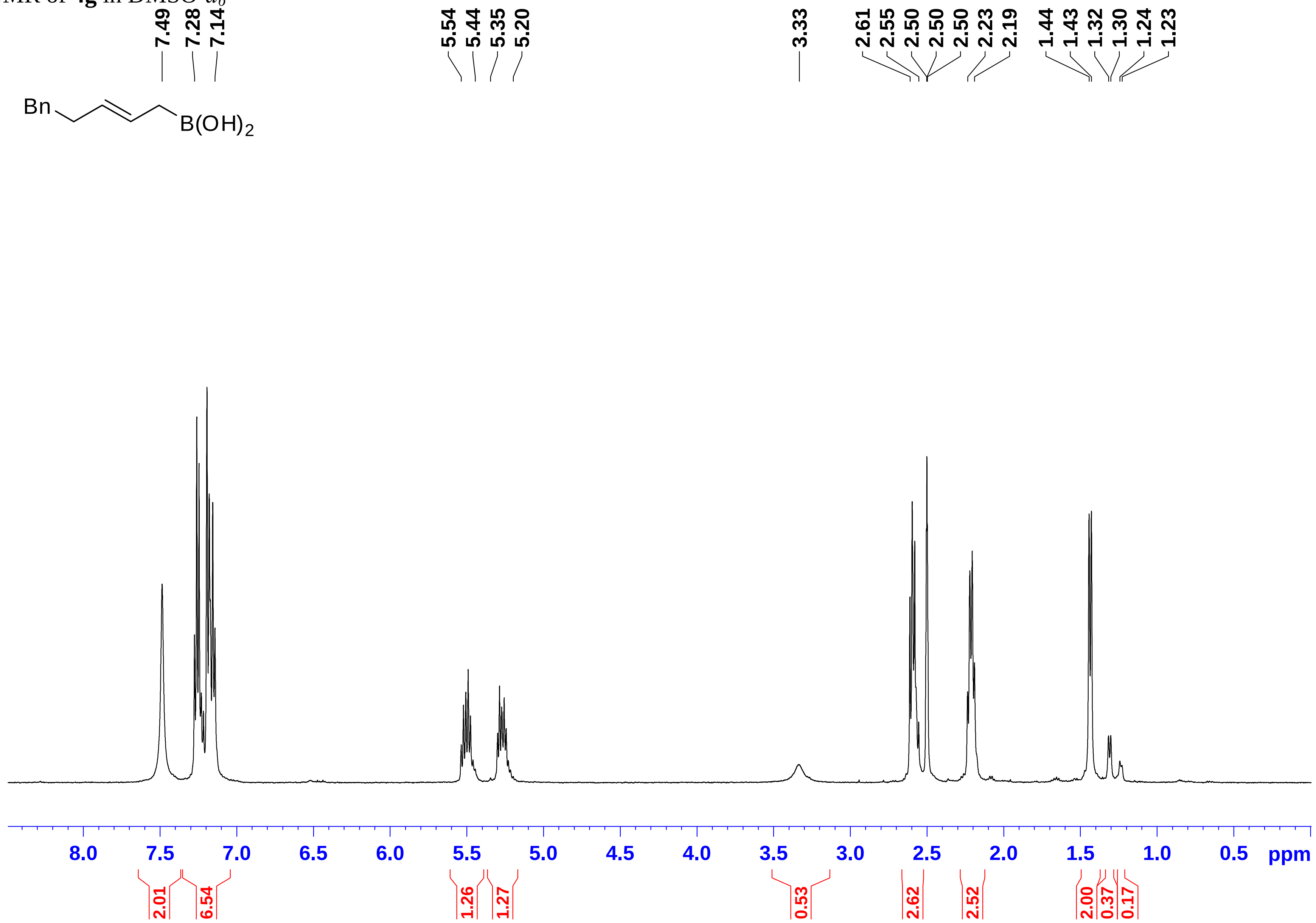

^{1}H NMR of **4g** in wet DMSO-d_6

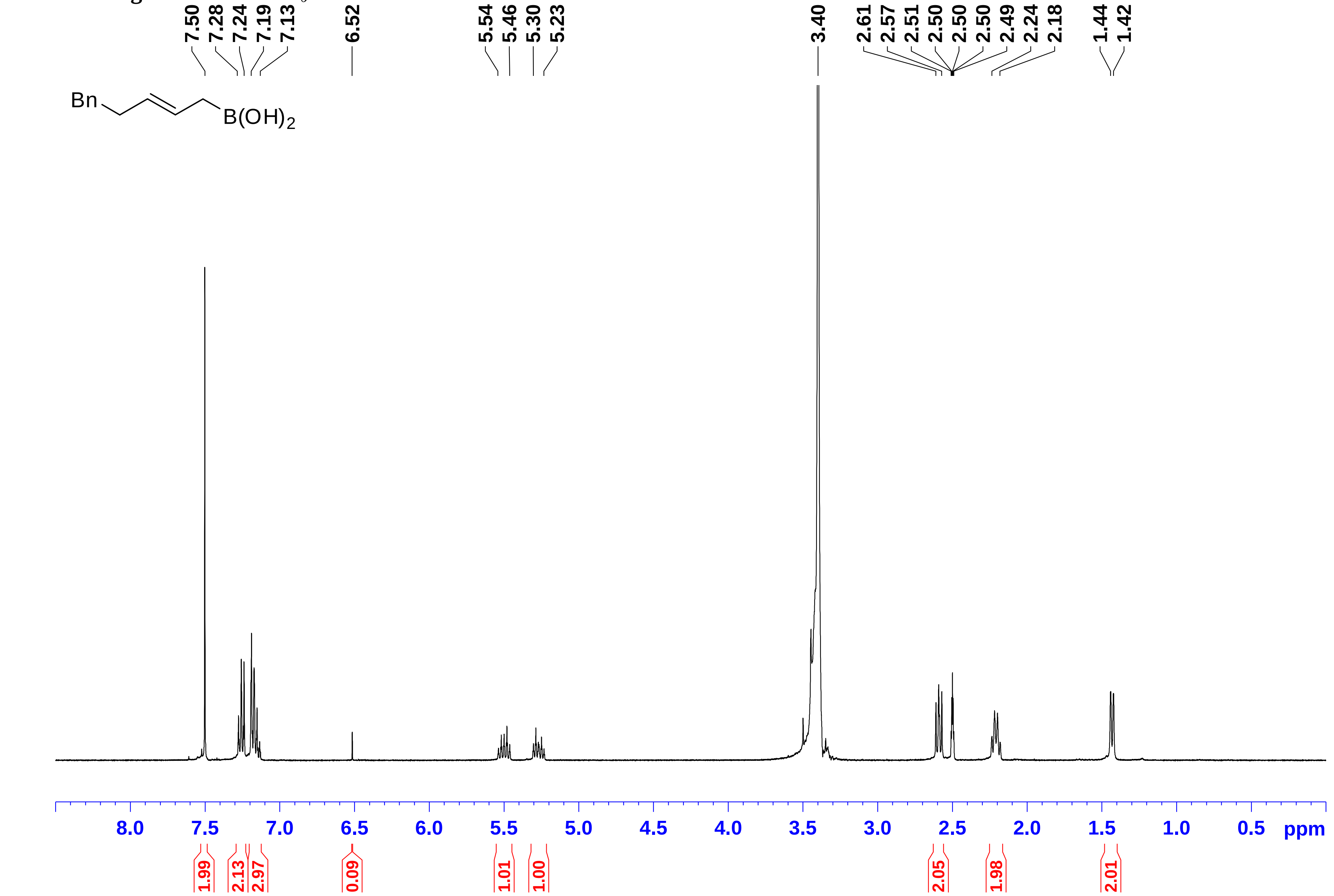

^{13}C NMR of **4g** in wet DMSO-d_6

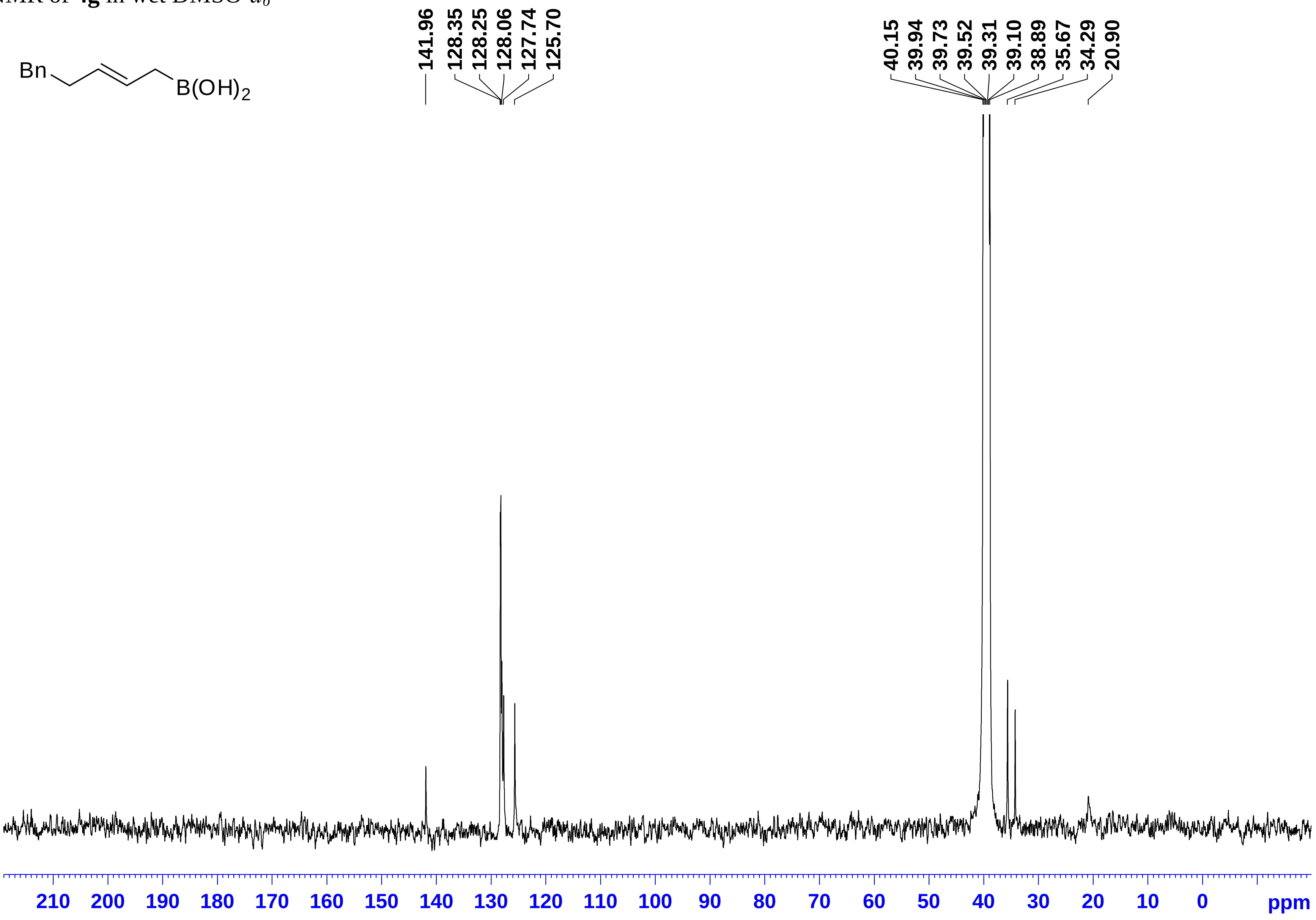

^{1}H NMR of **4h** in DMSO-d_6

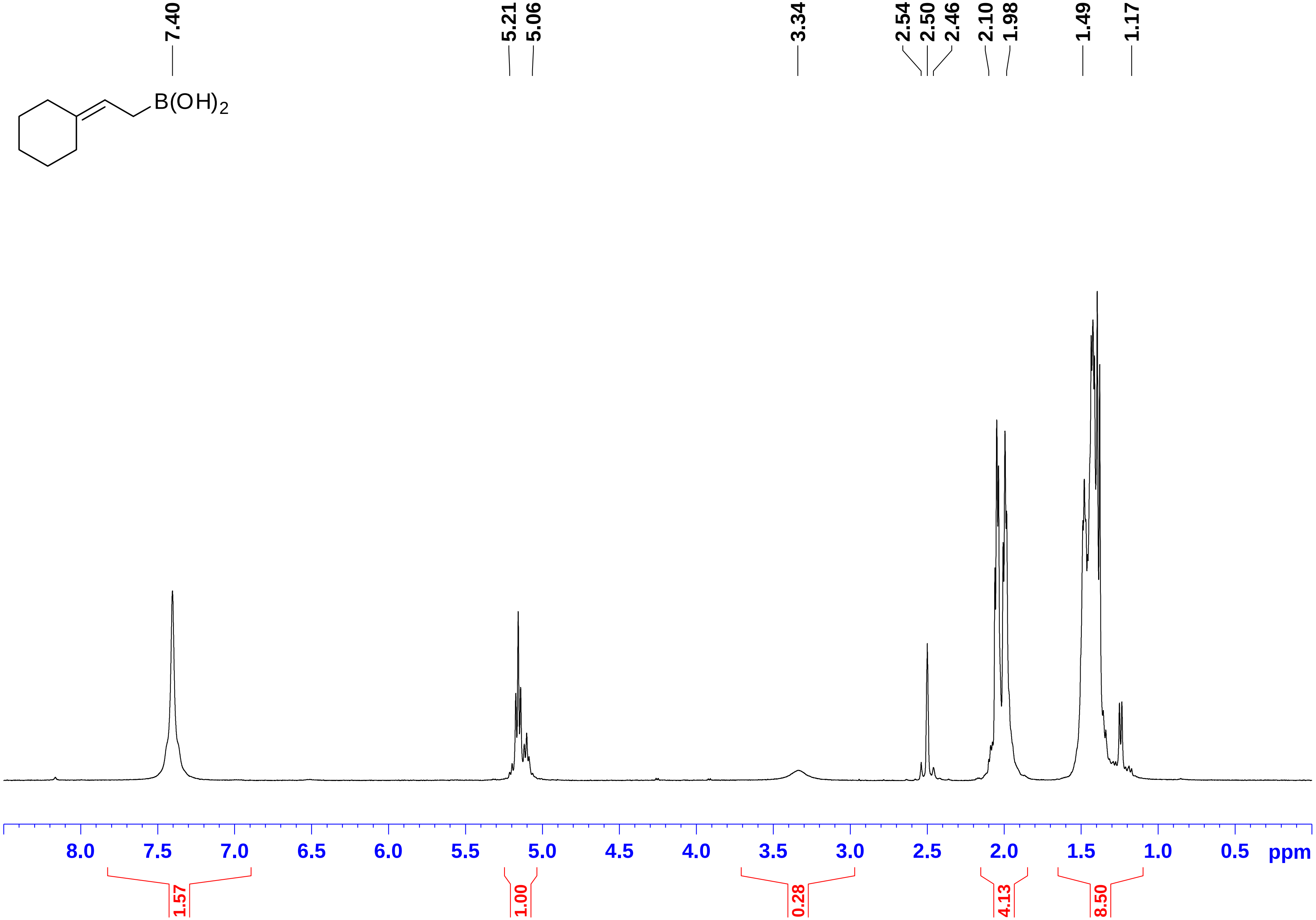

^{1}H NMR of **4h** in wet DMSO-d_6

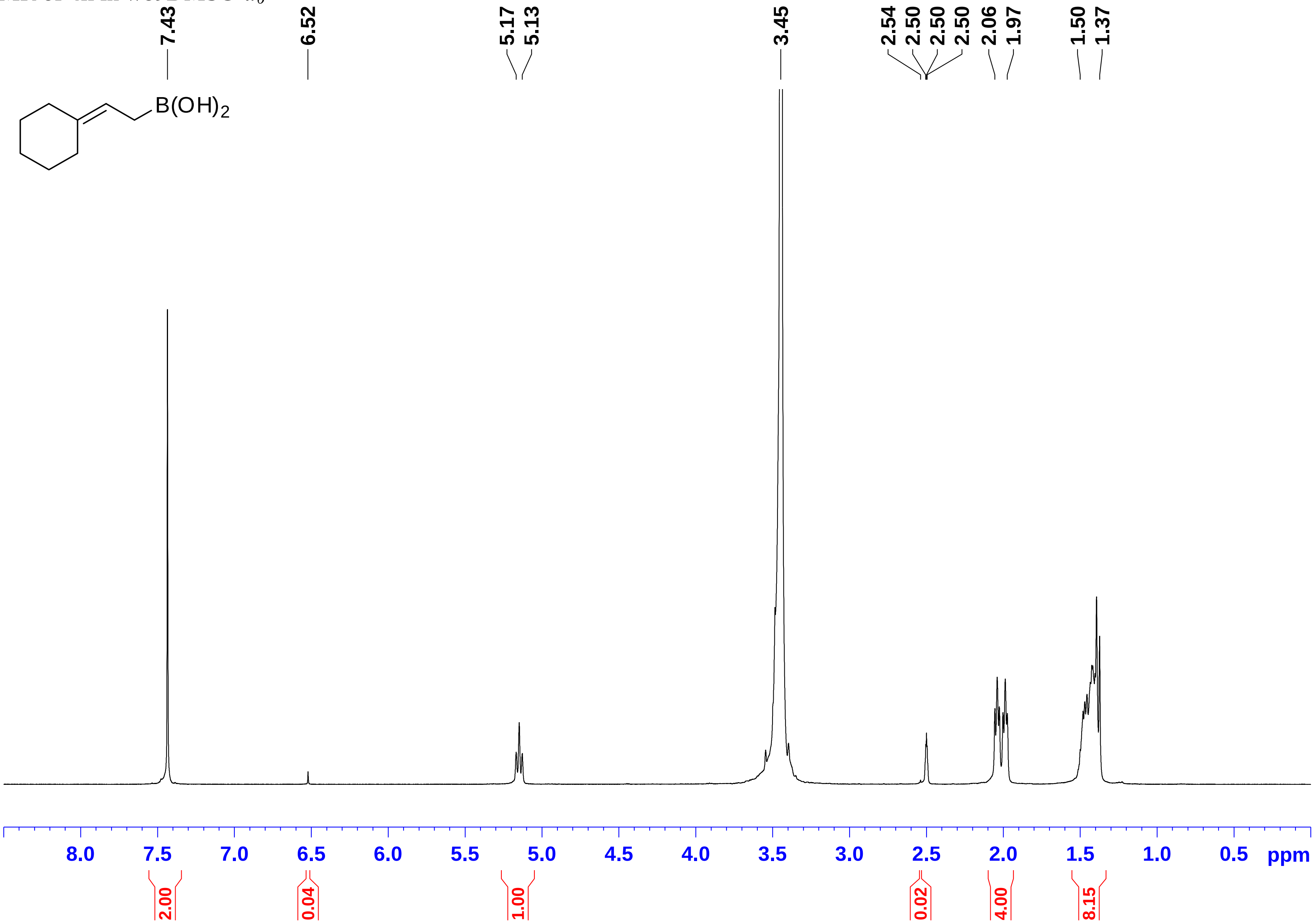

^{13}C NMR of **4h** in wet DMSO-d_6

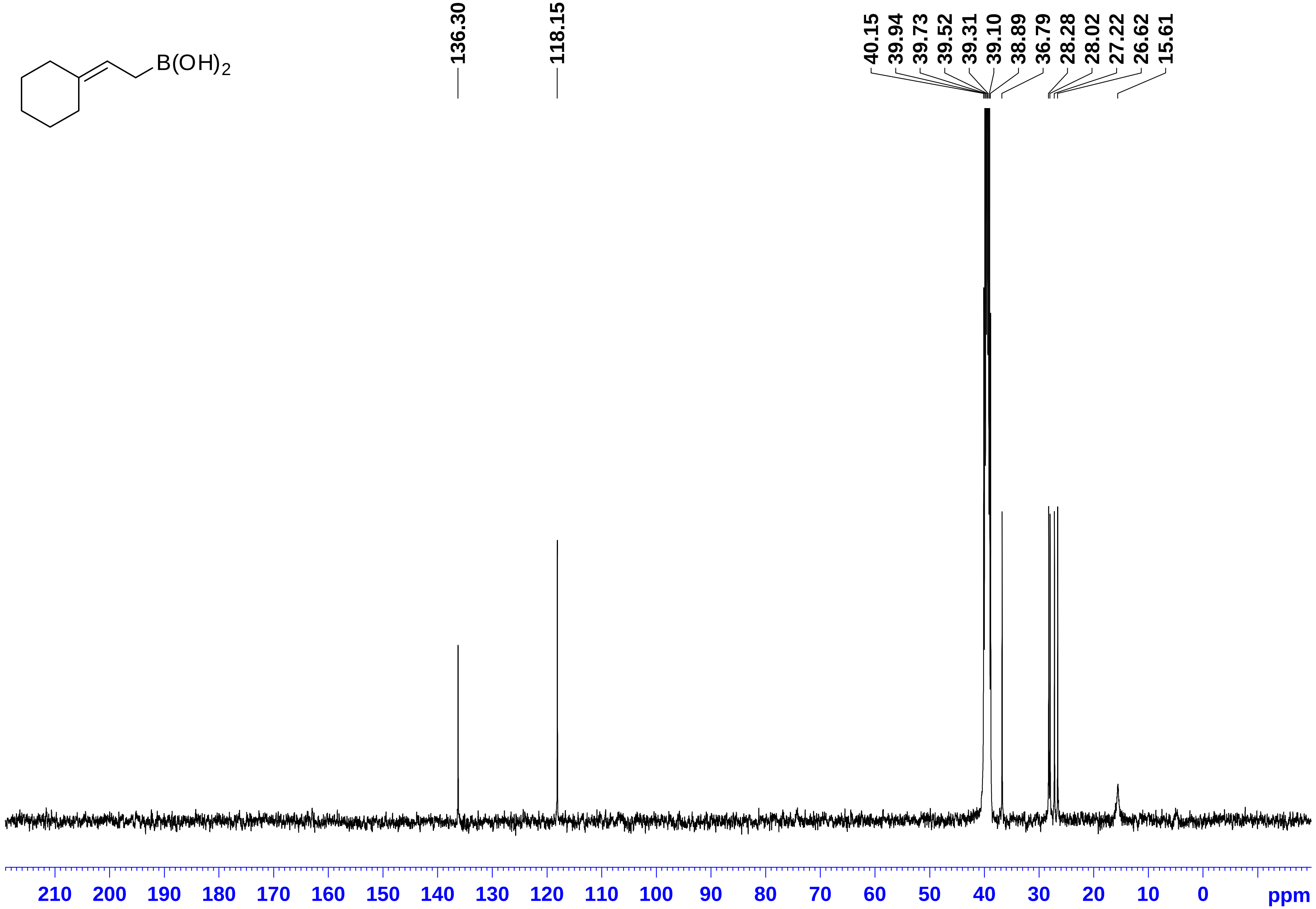

^{1}H NMR of **4i** in DMSO-d_6

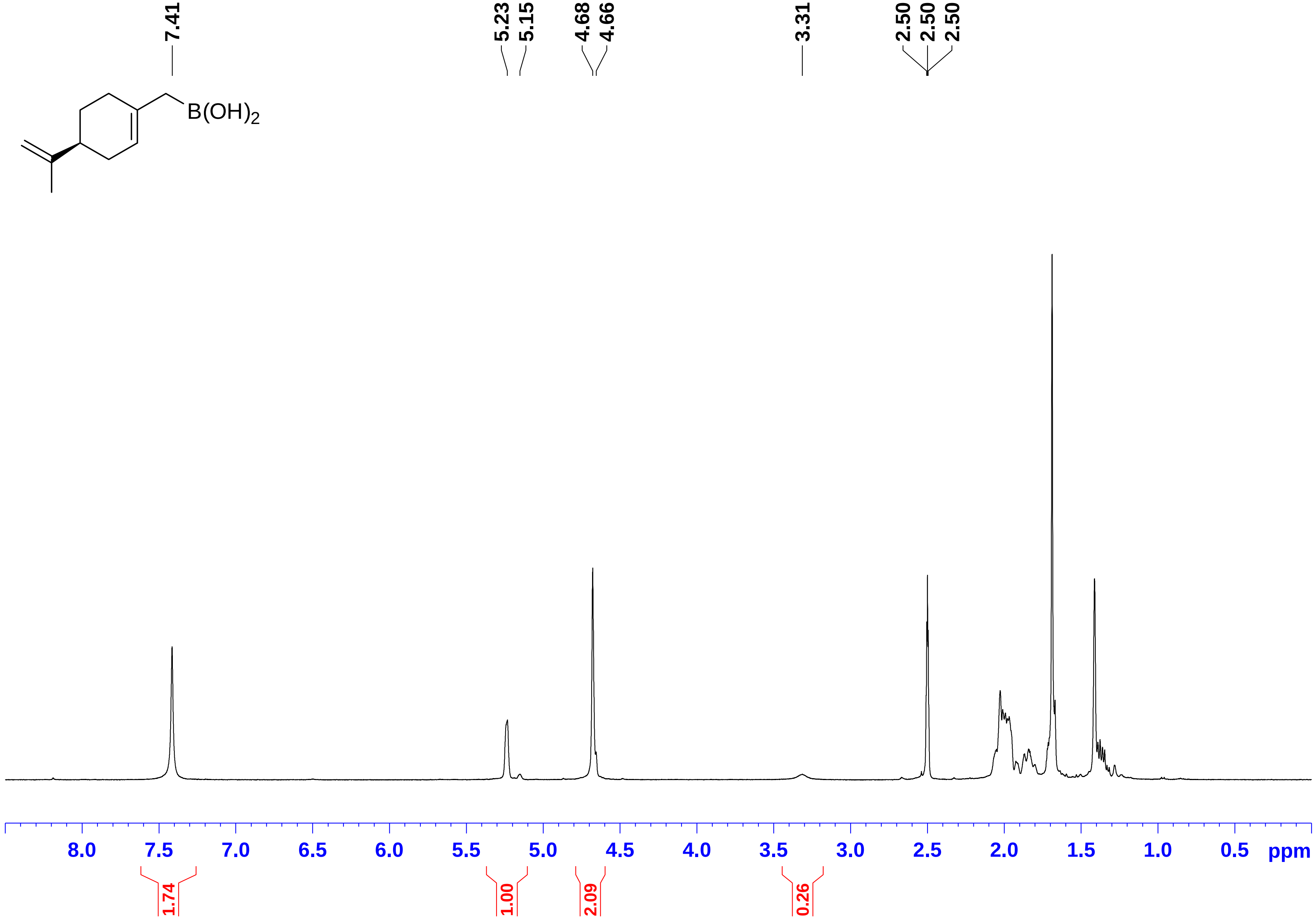

^{1}H NMR of **4i** in wet DMSO-d_6

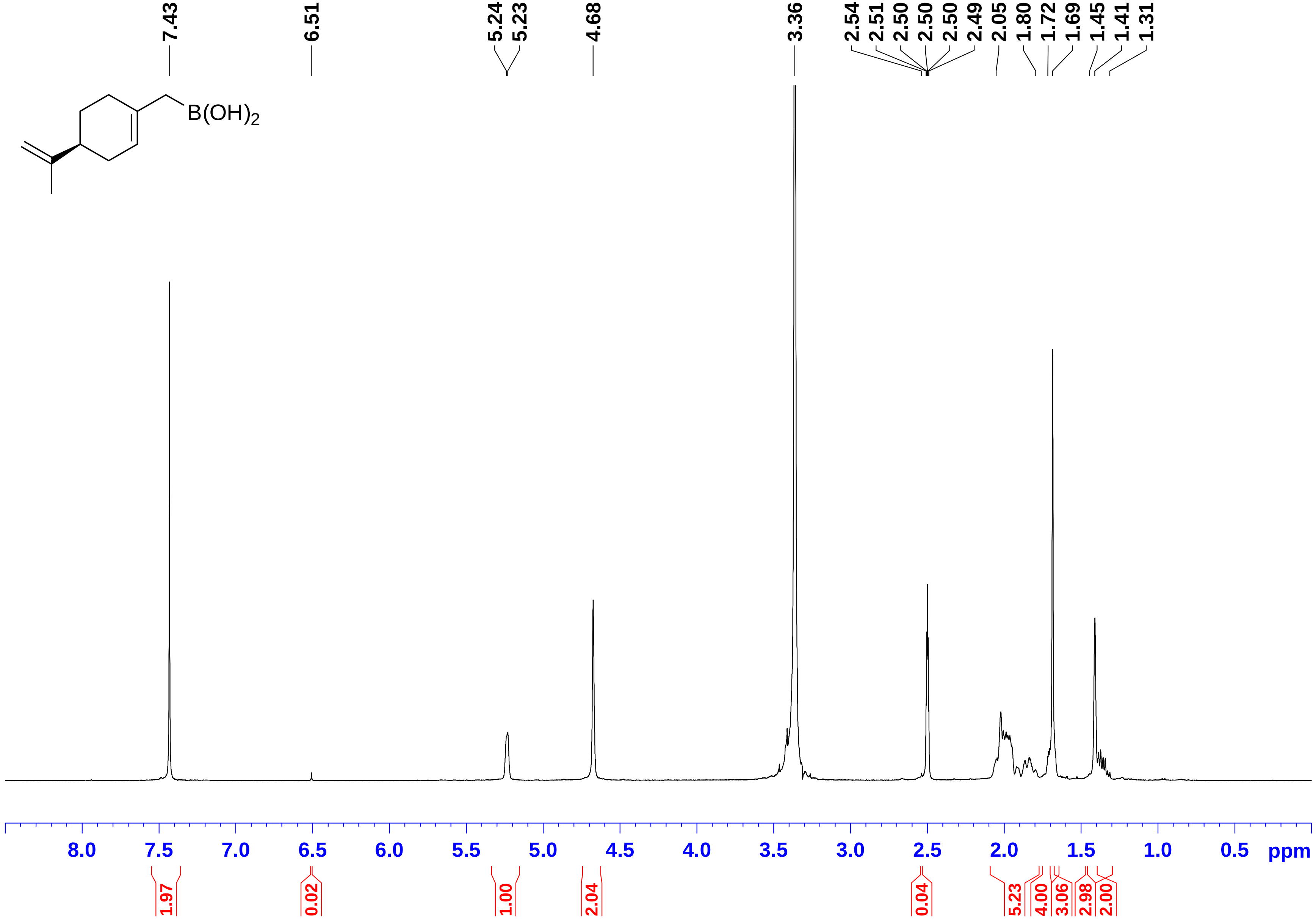

^{13}C NMR of **4i** in wet DMSO-d_6

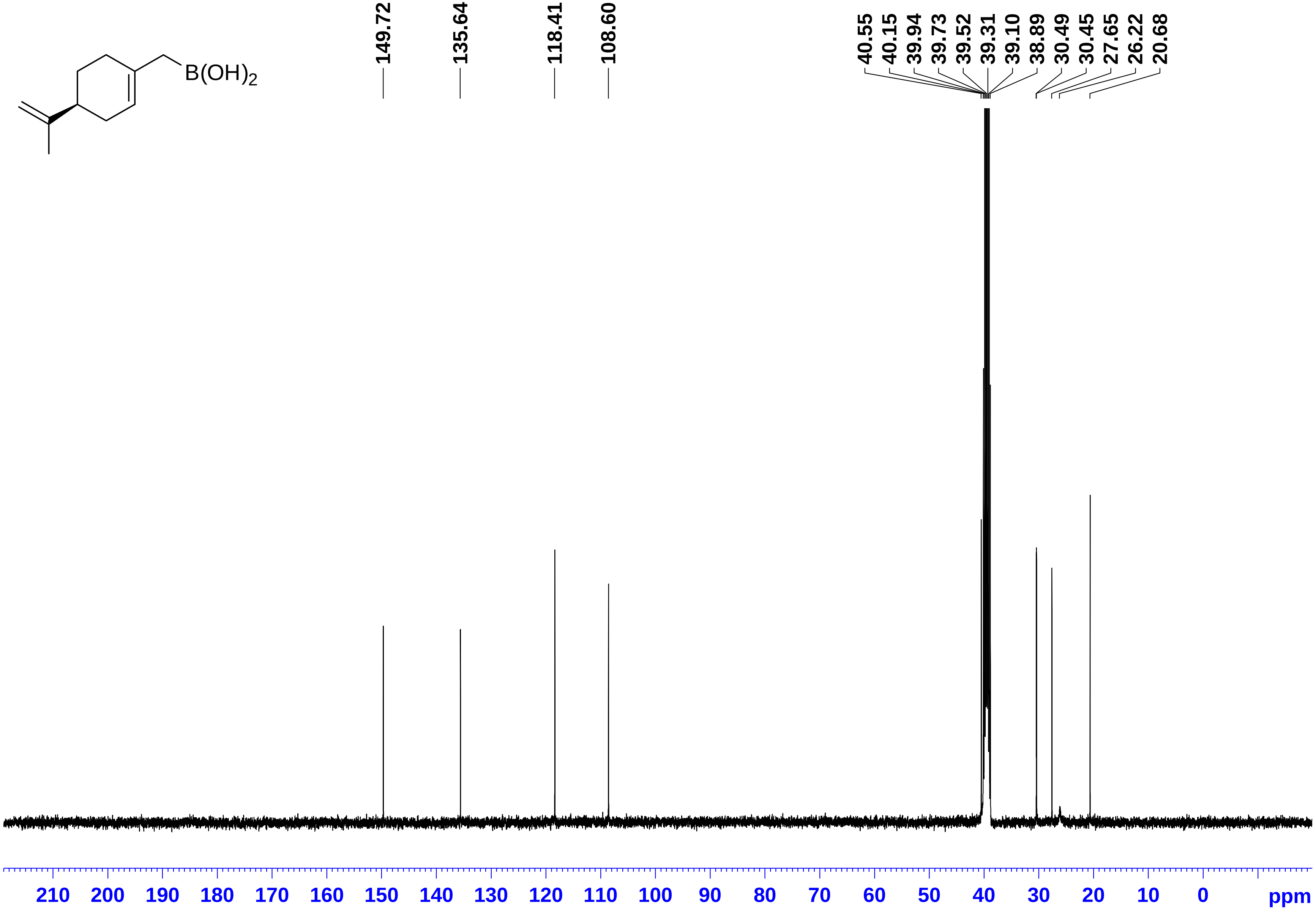

^{1}H NMR of **4j** in $CHCl_3/CDCl_3$

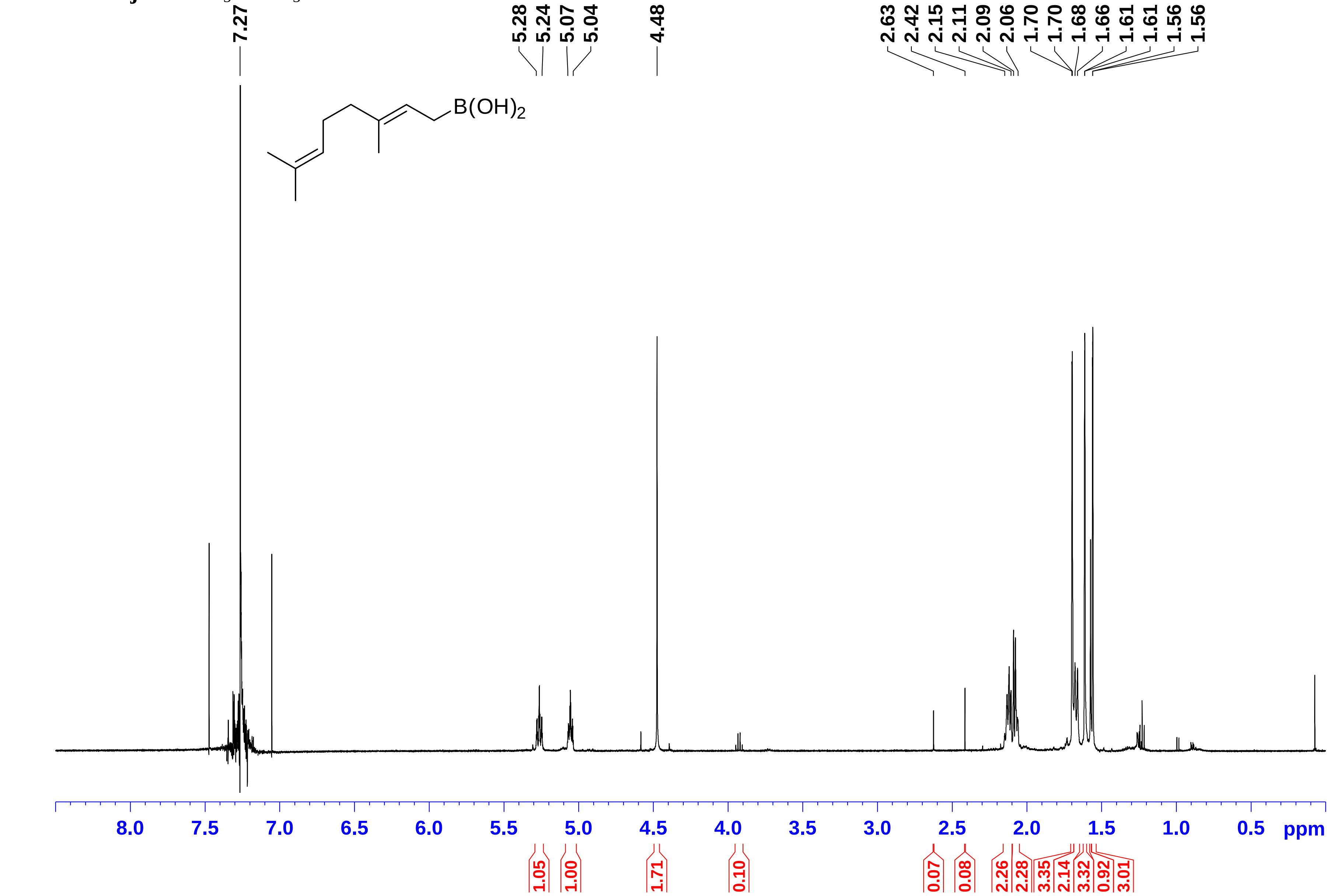

^{13}C NMR of **4j** in $CHCl_3/CDCl_3$

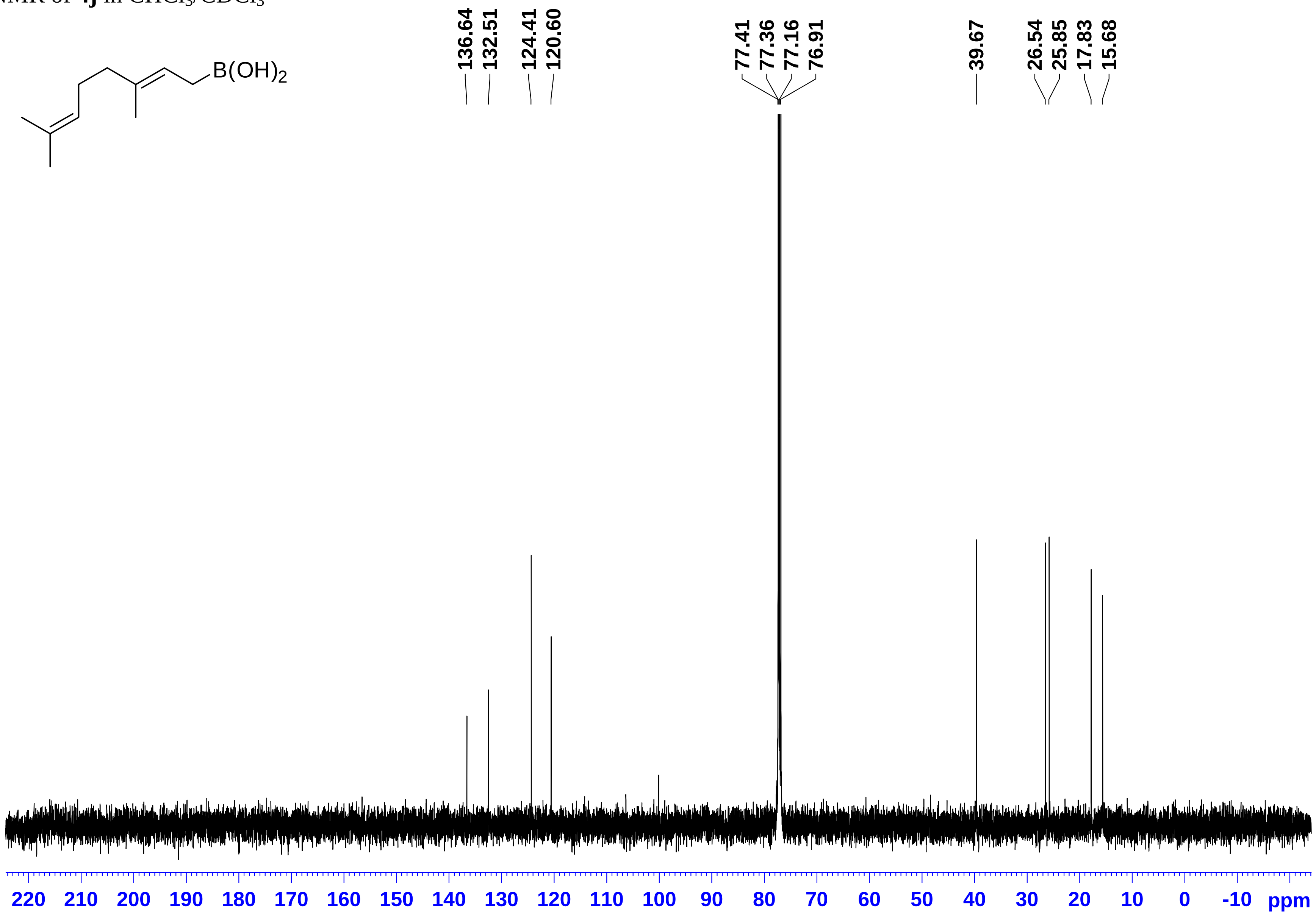

^{1}H NMR of **4k** in $CHCl_3/CDCl_3$

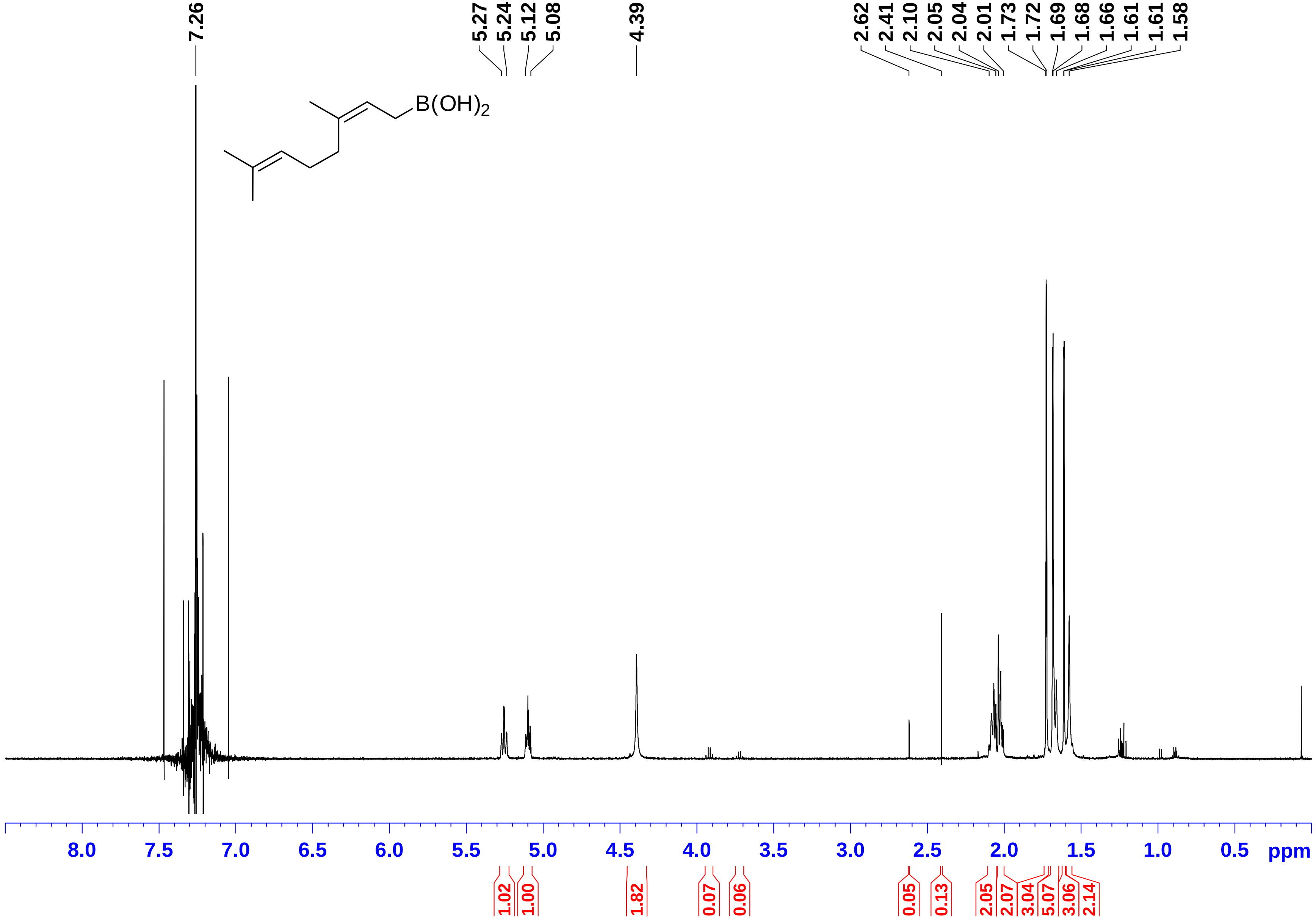

^{13}C NMR of **4k** in $CHCl_3/CDCl_3$

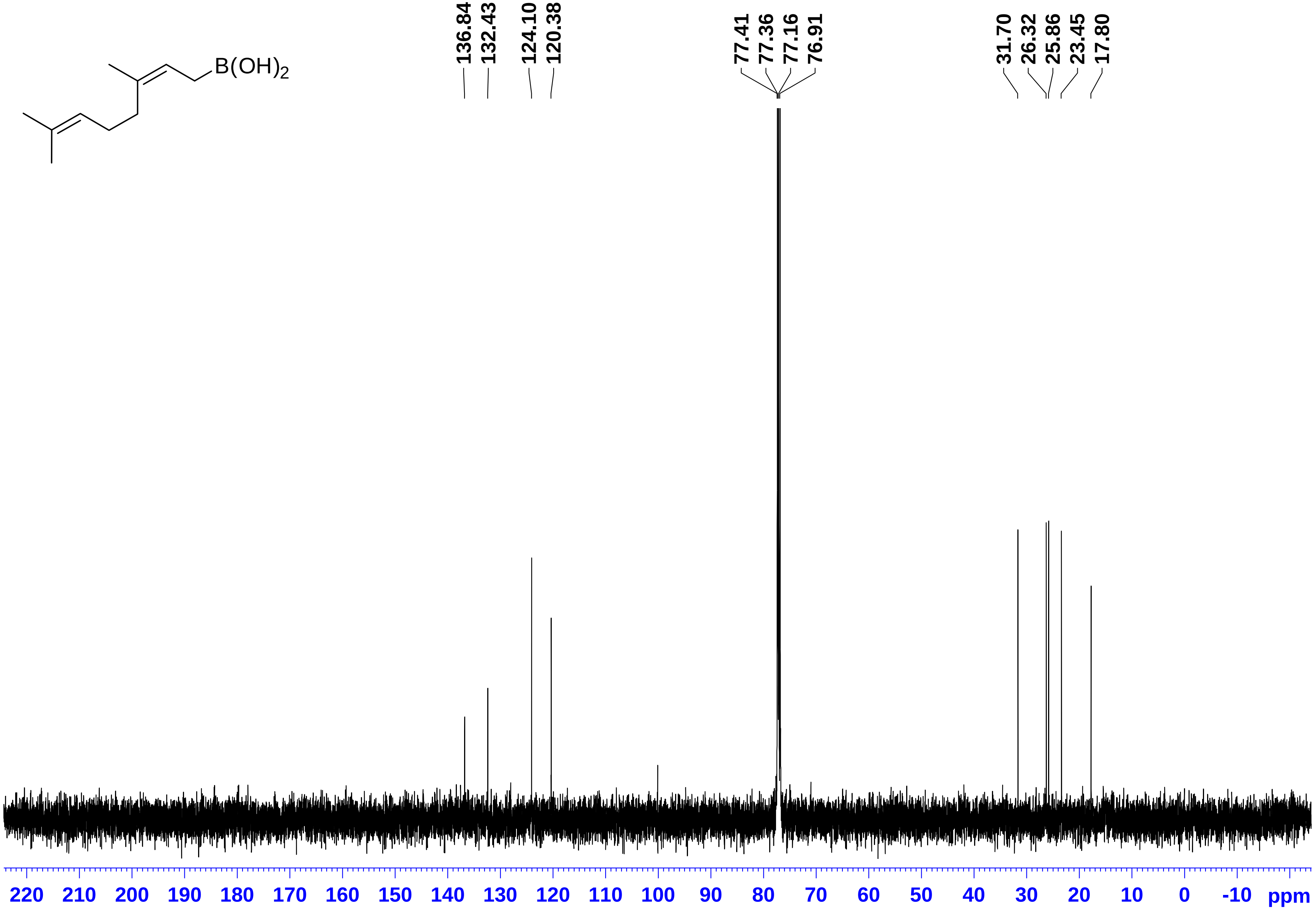

^{1}H NMR of **4j** (red) versus **4k** (blue) in wet DMSO/DMSO-d_6 (as observed at the end of the reaction)

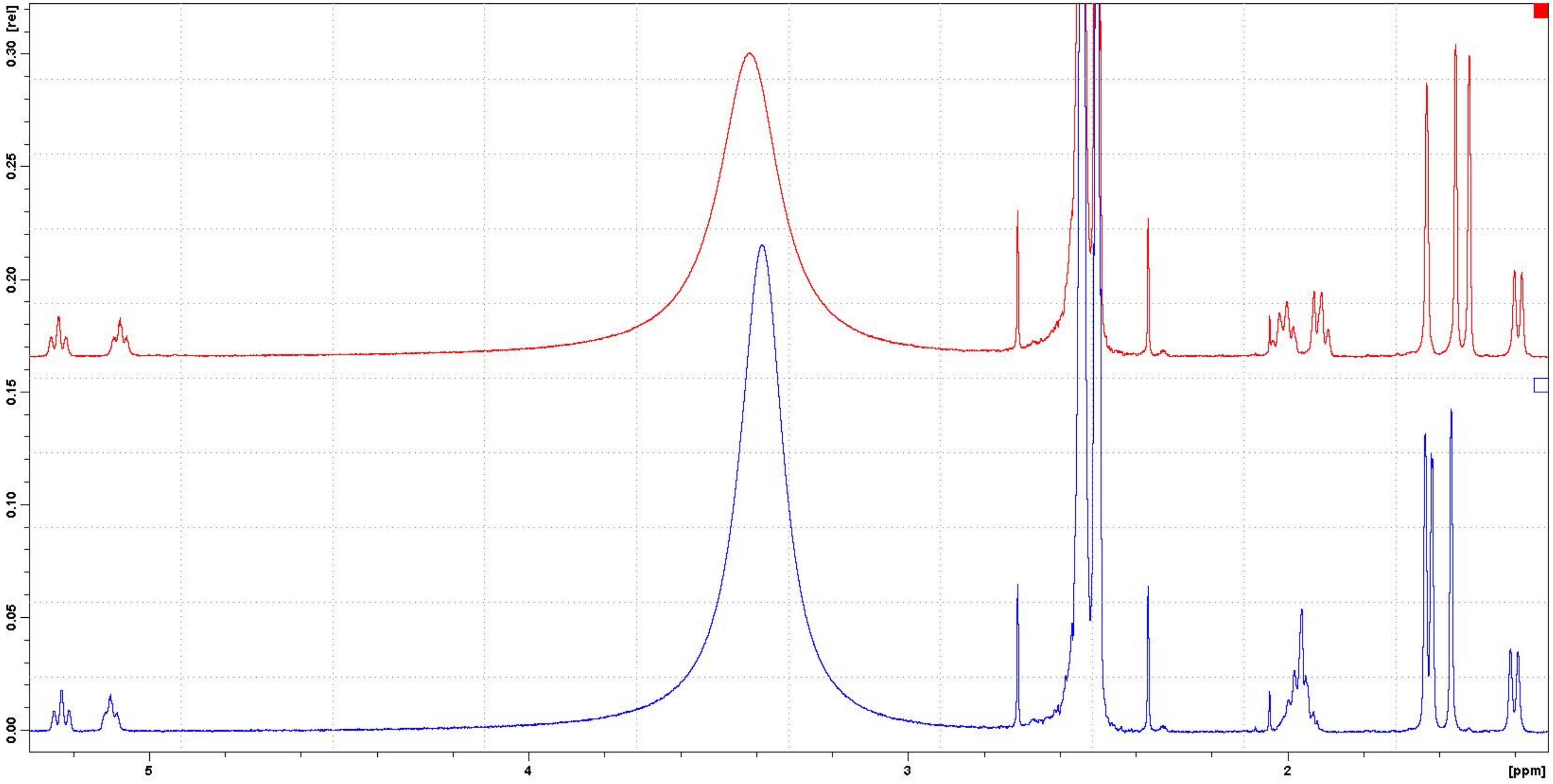

^{1}H NMR of **4j** (red) versus **4k** (blue) in $CHCl_3/CDCl_3$

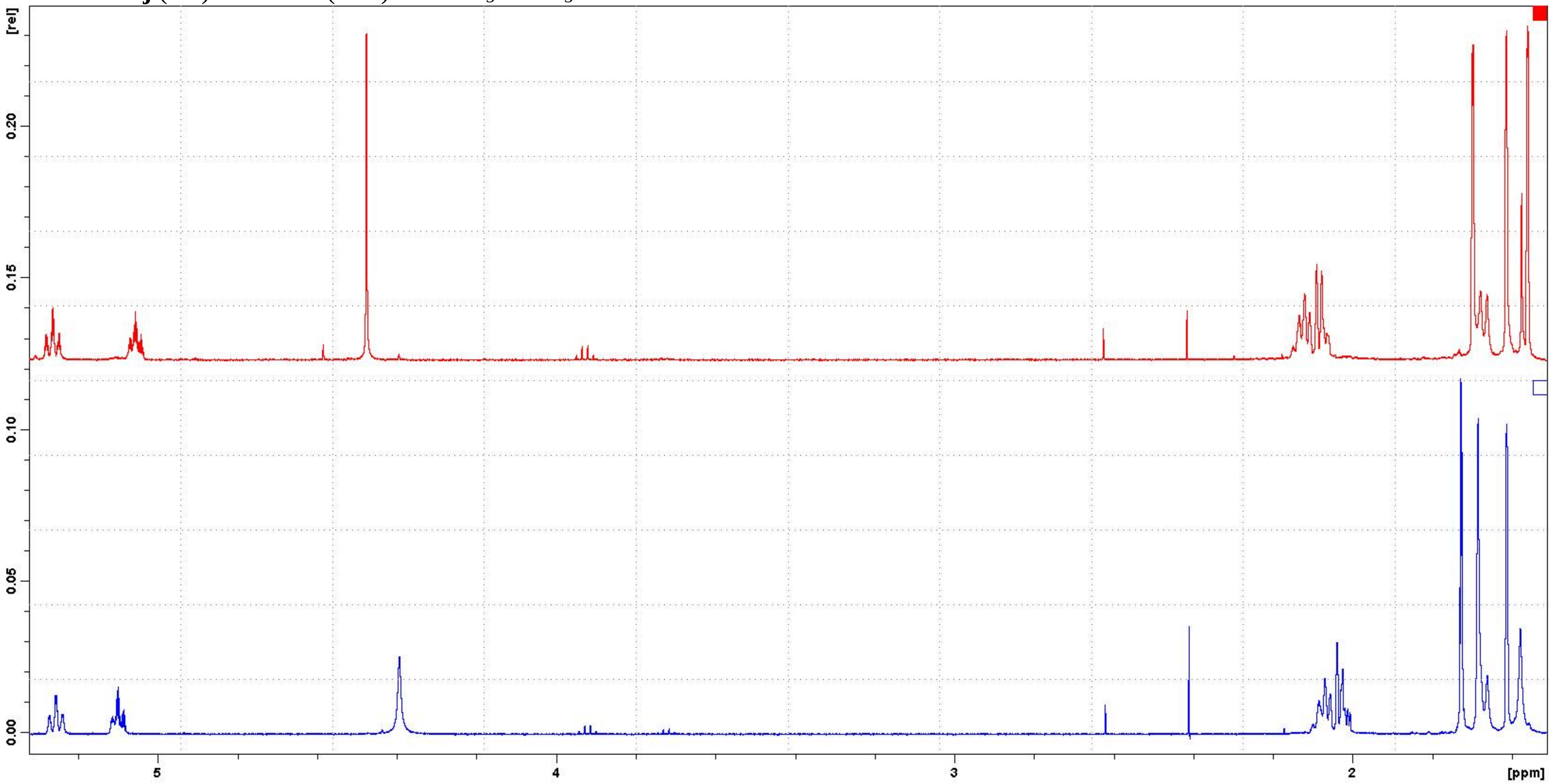

^{13}C NMR of **4j** (red) versus **4k** (blue) in $CHCl_3/CDCl_3$

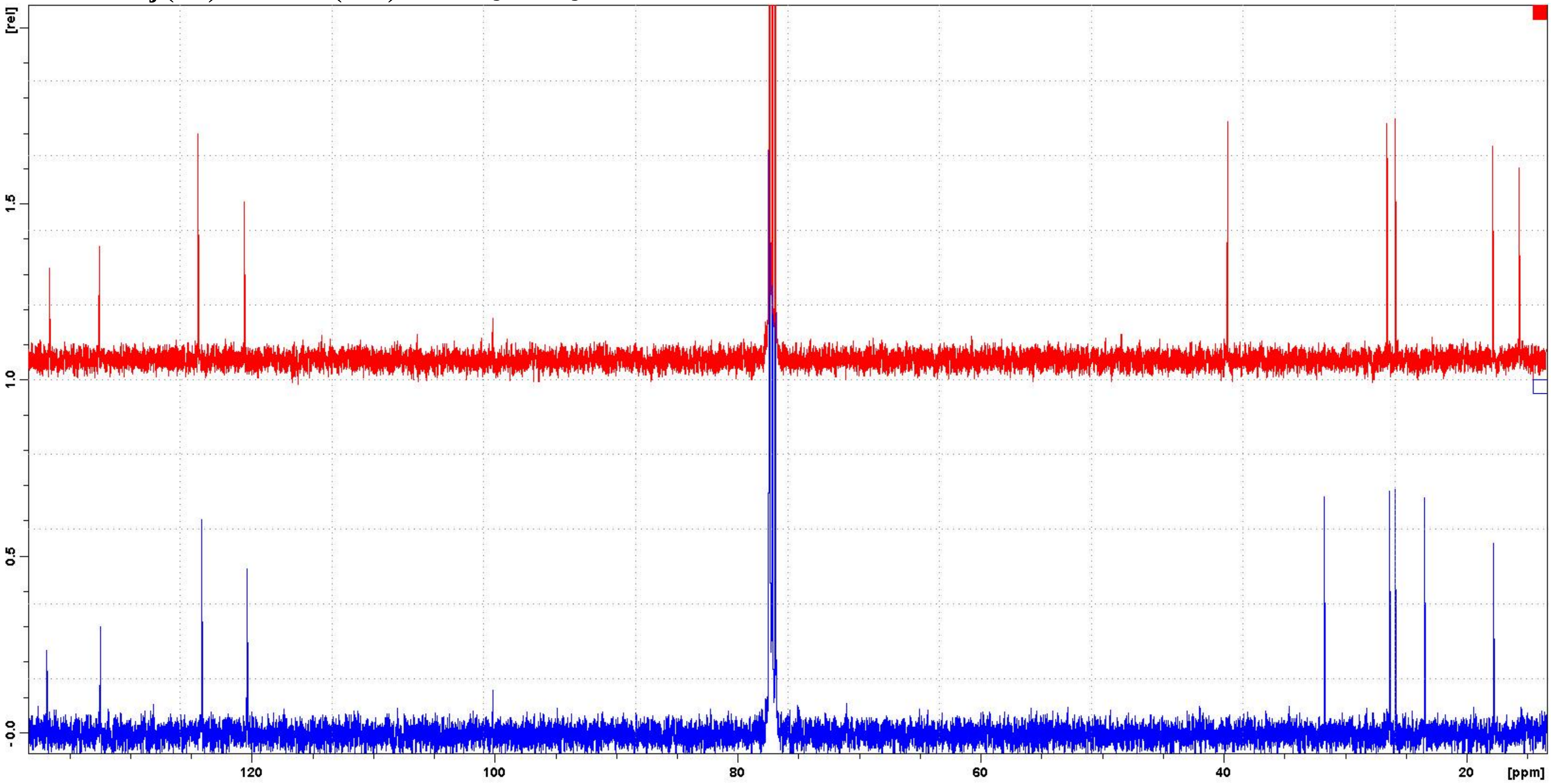

^{1}H NMR of **4l** in DMSO-d_6

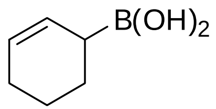

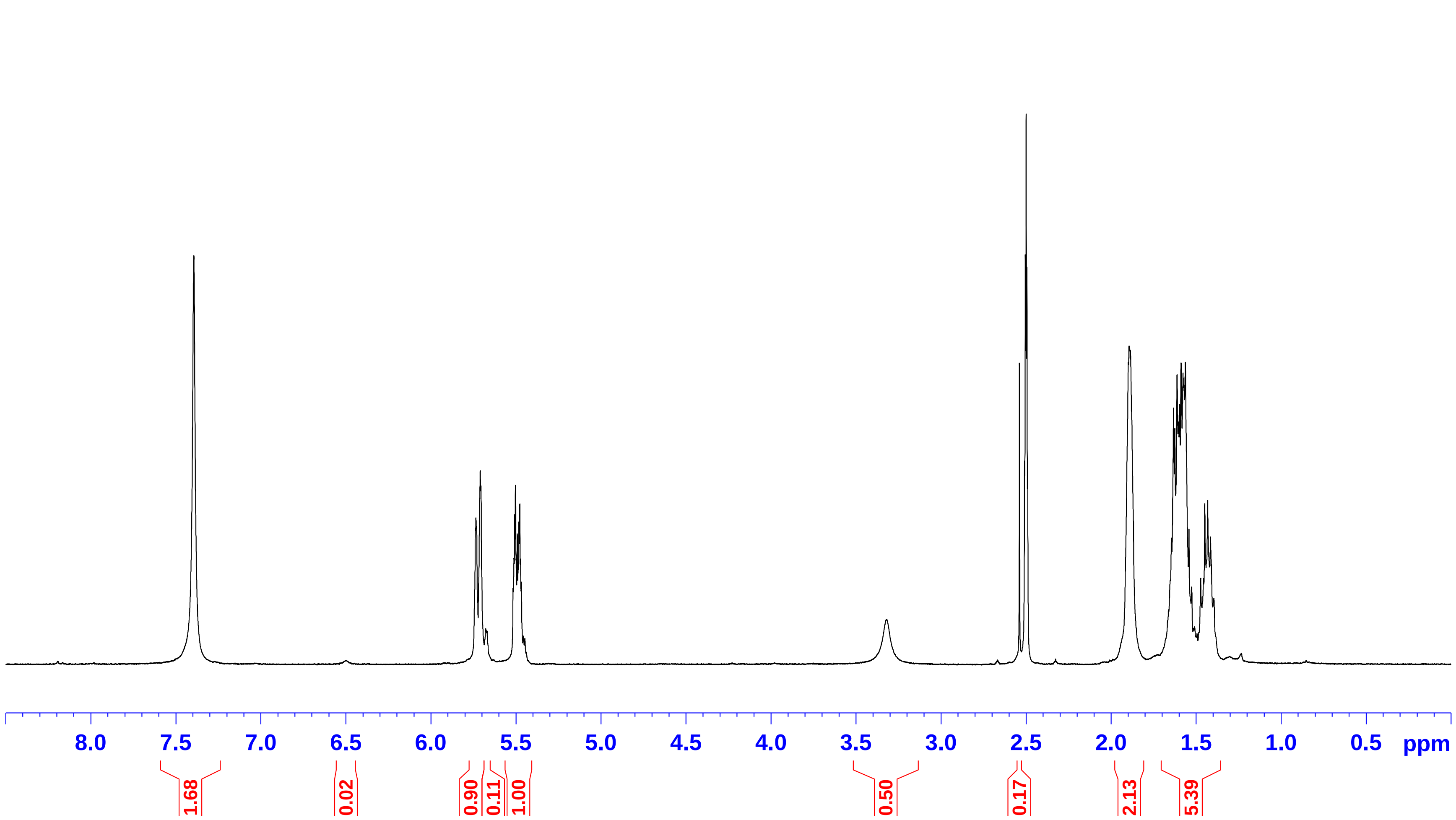

^{1}H NMR of **4l** in wet DMSO-d_6

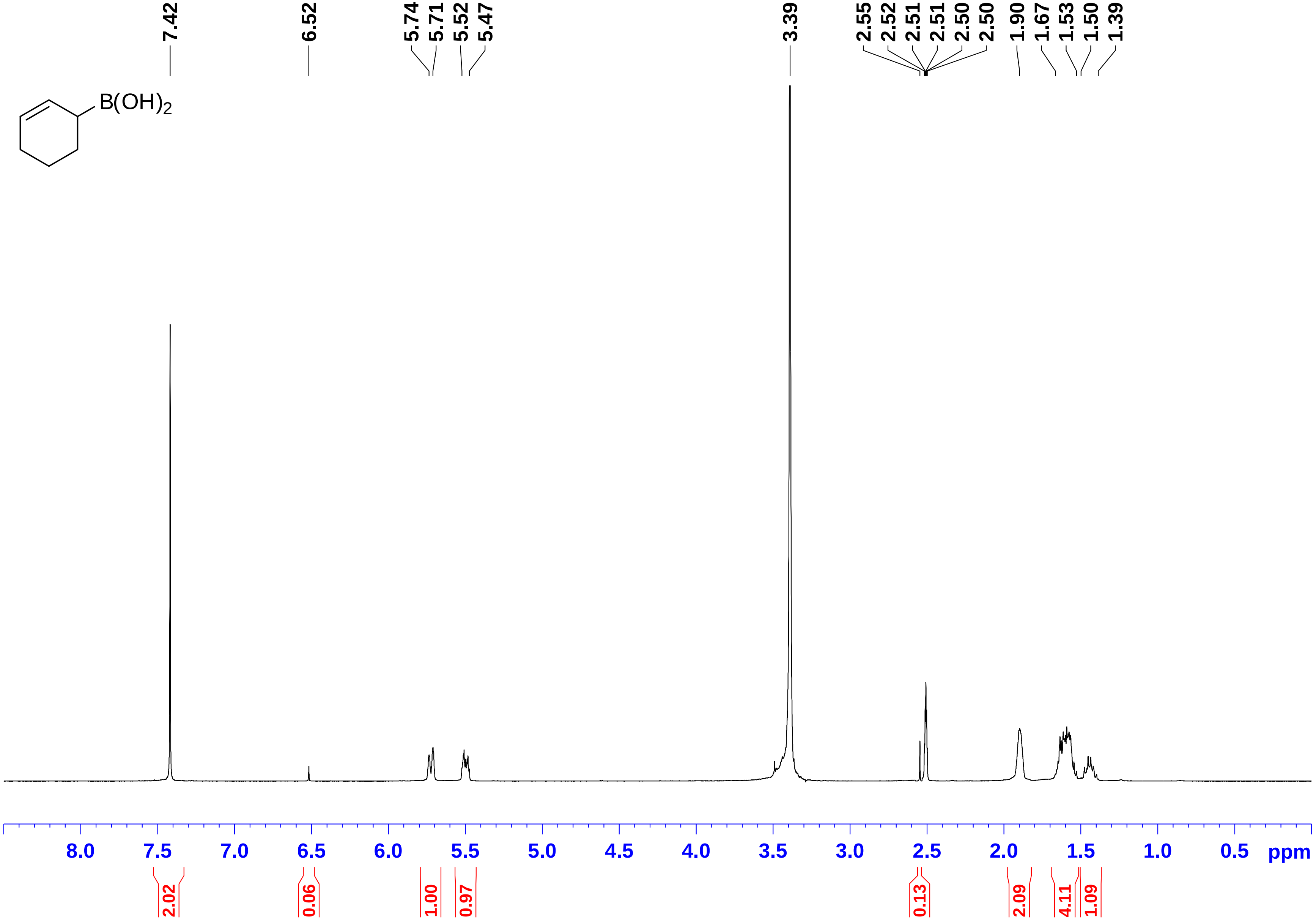

^{13}C NMR of **4l** in wet DMSO-d_6

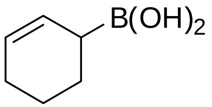

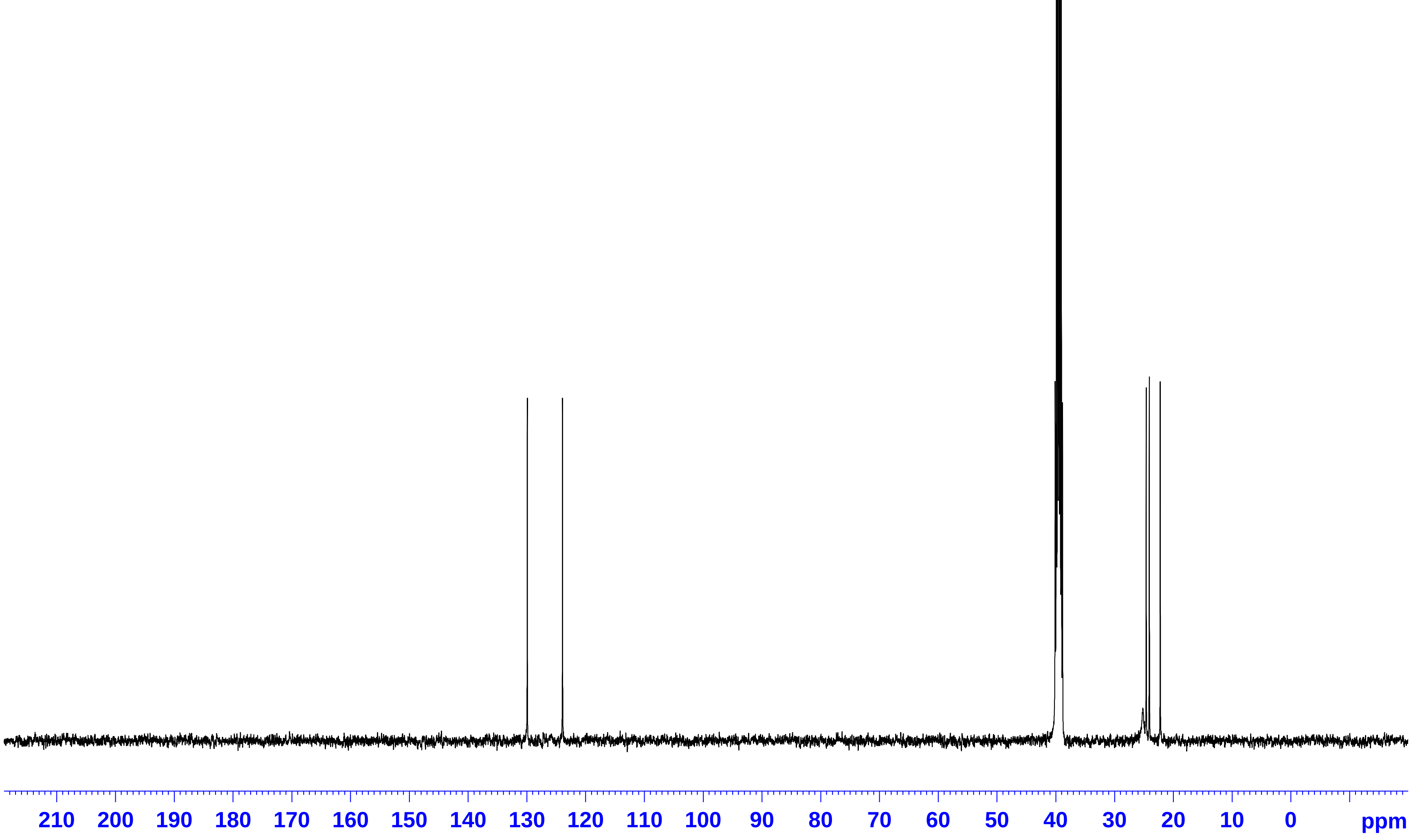

^{1}H NMR of **6a** in $CDCl_3$

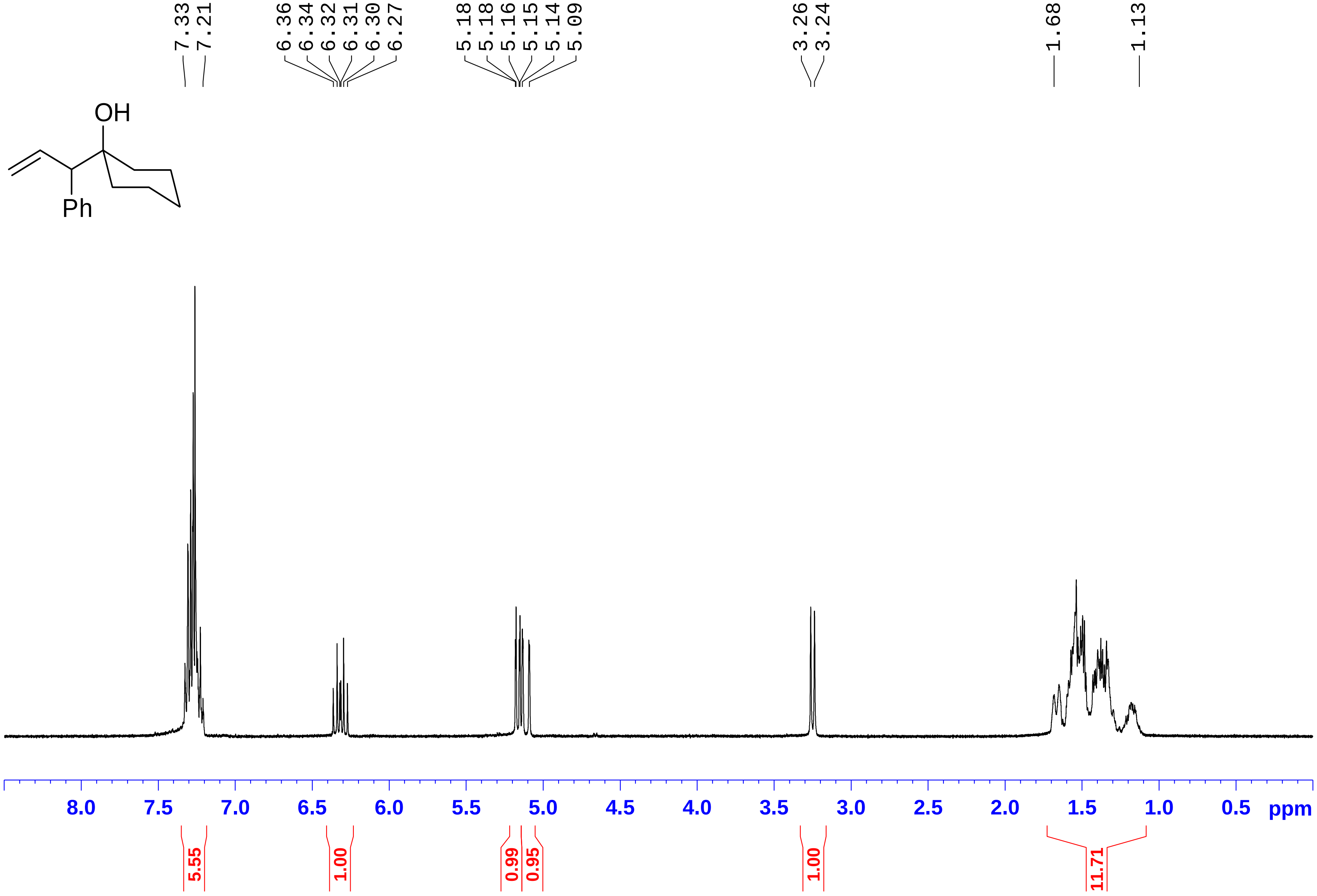

^{13}C NMR of **6a** in $CDCl_3$

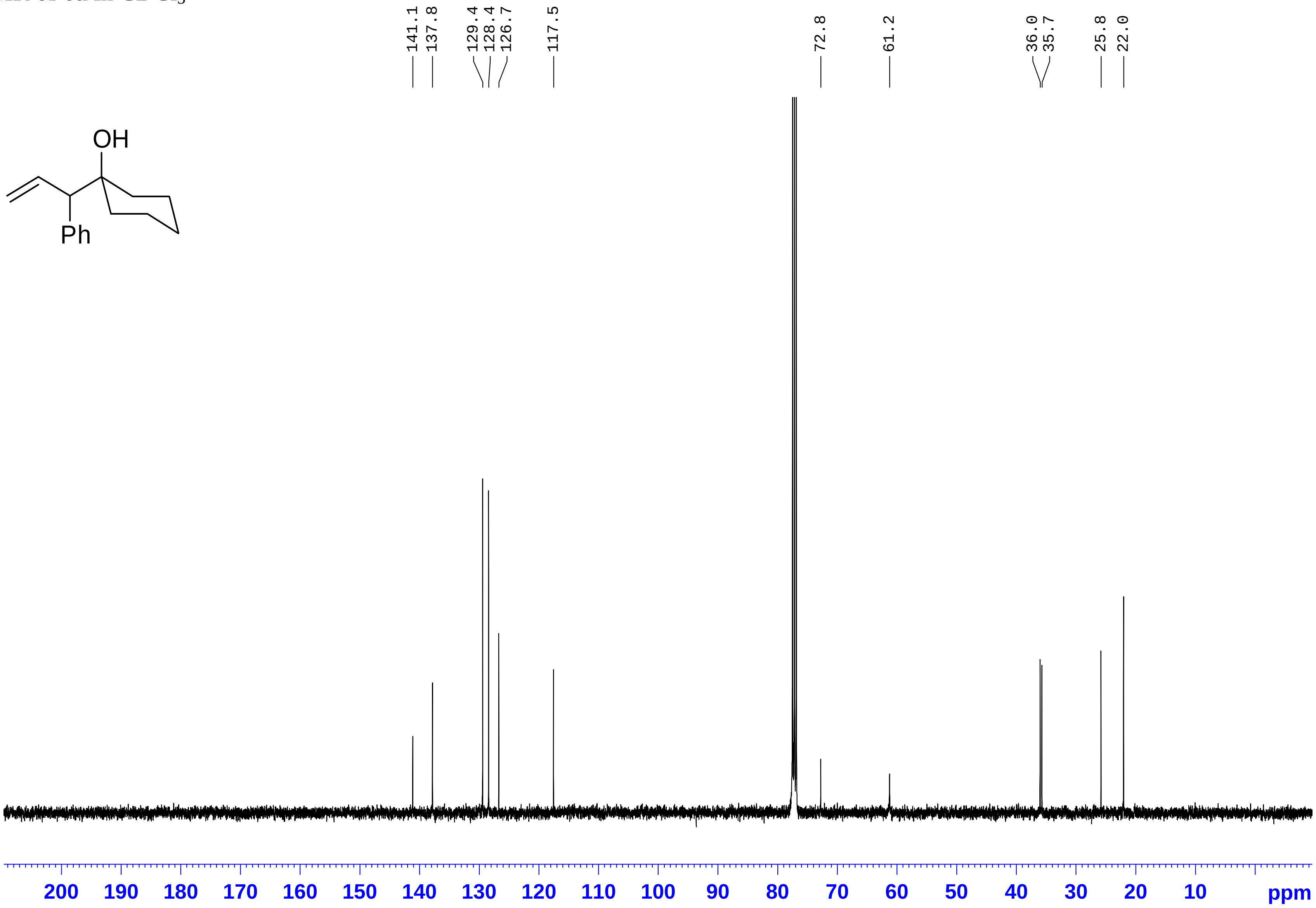

^{1}H NMR of **6b** in $CDCl_3$

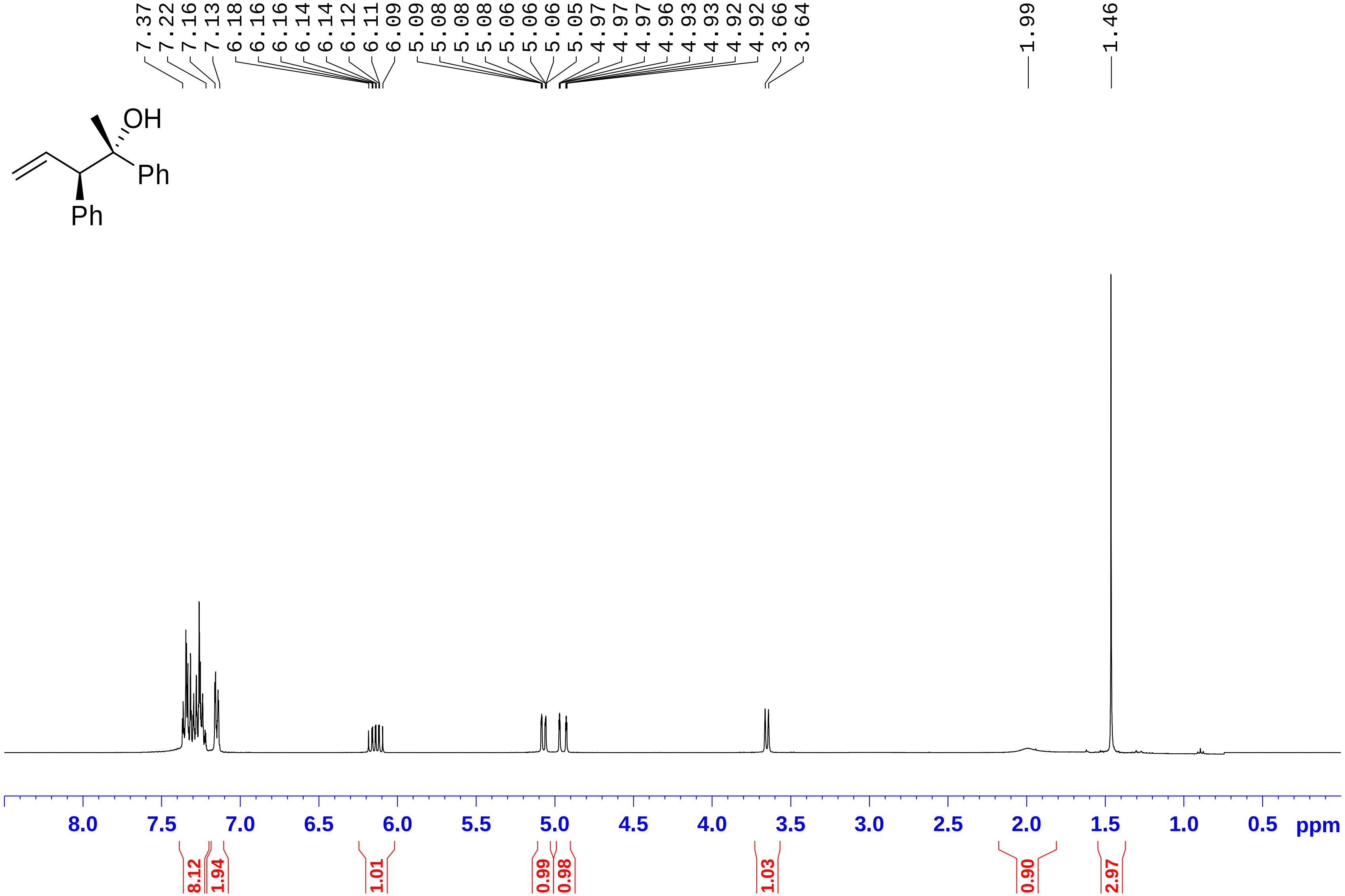

^{13}C NMR of **6b** in $CDCl_3$

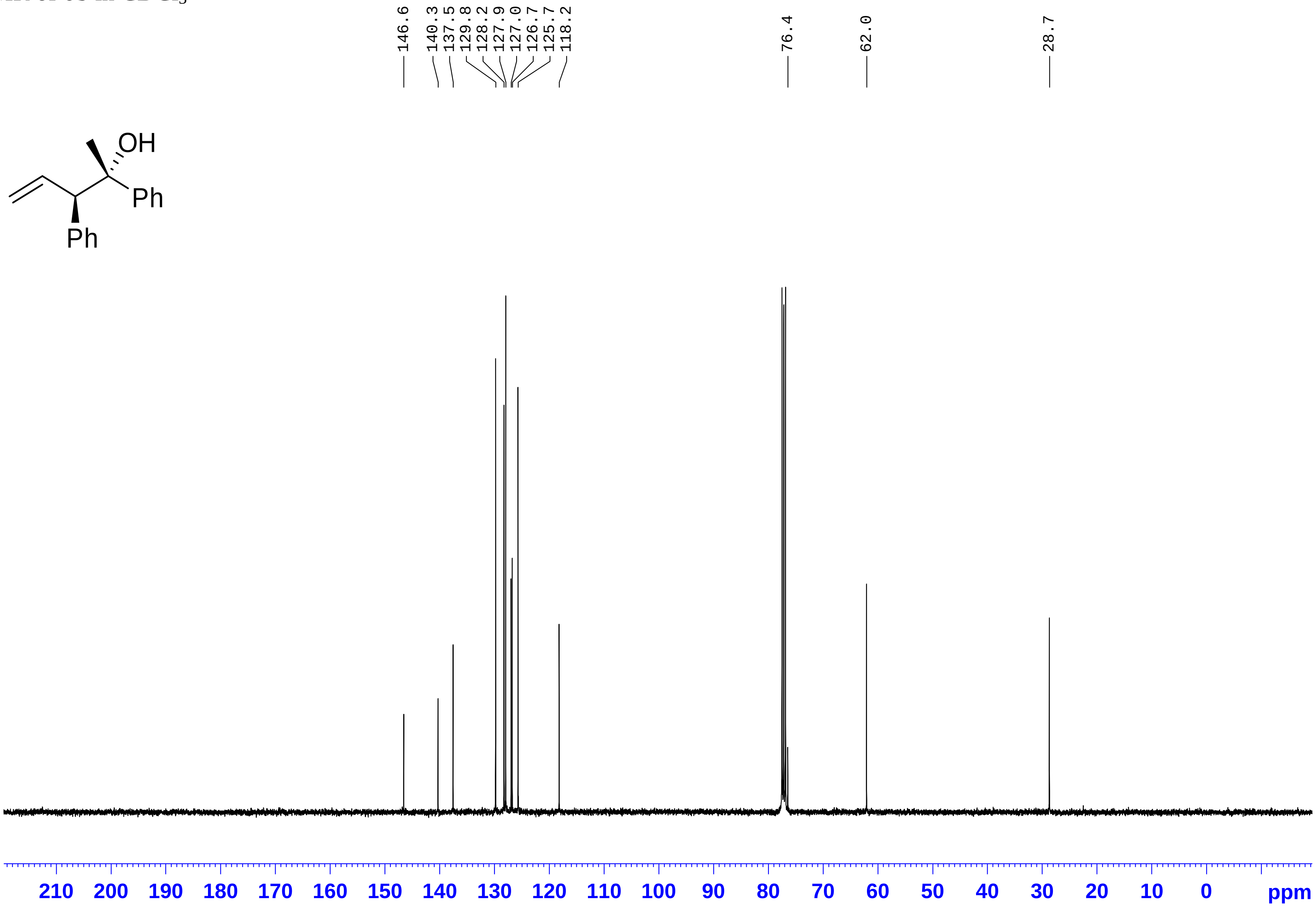

^{1}H NMR of **6c** in $CDCl_3$

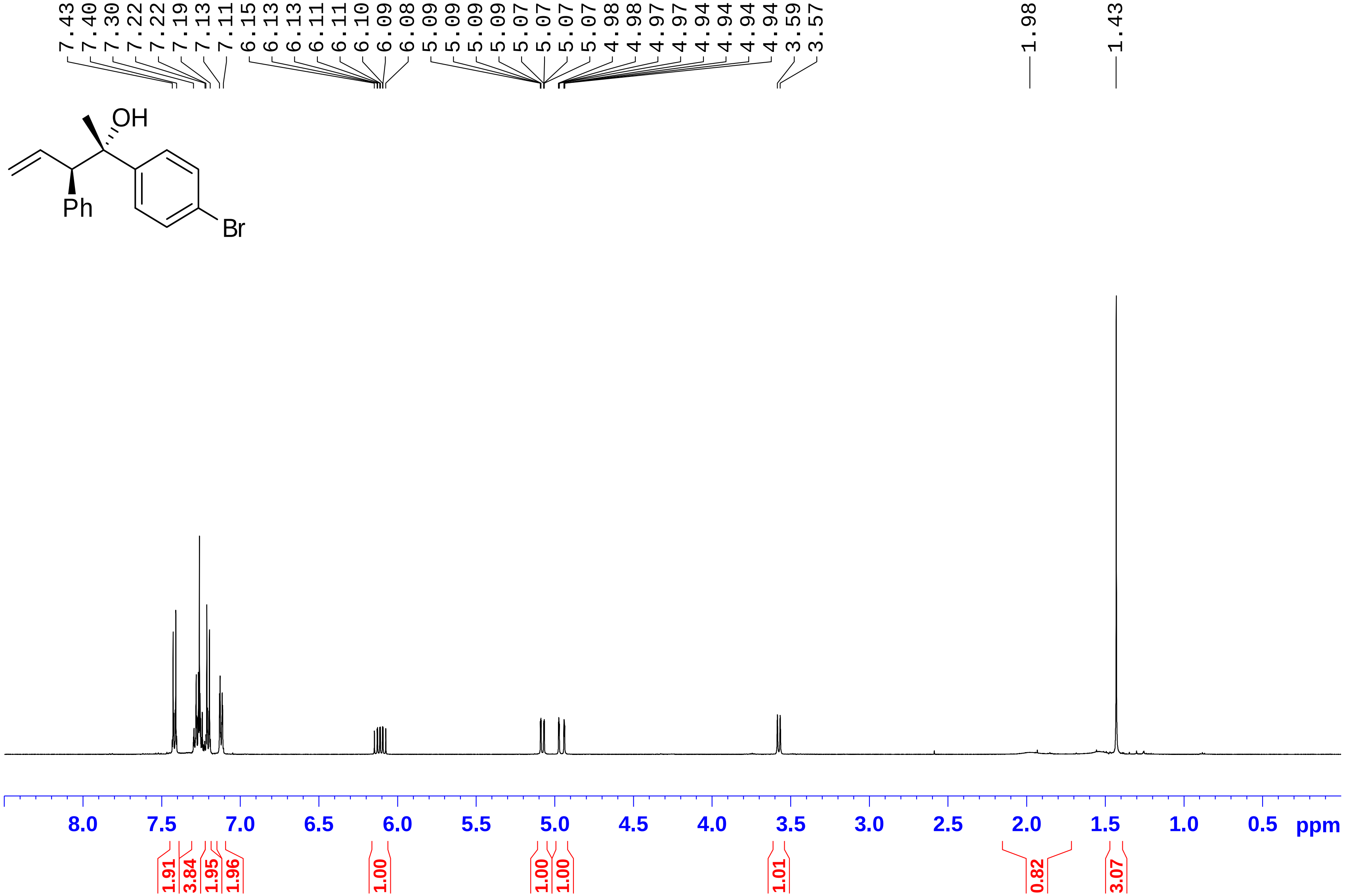

^{13}C NMR of **6c** in $CDCl_3$

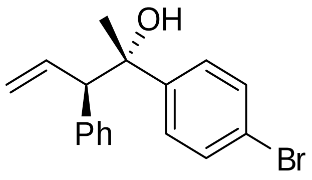

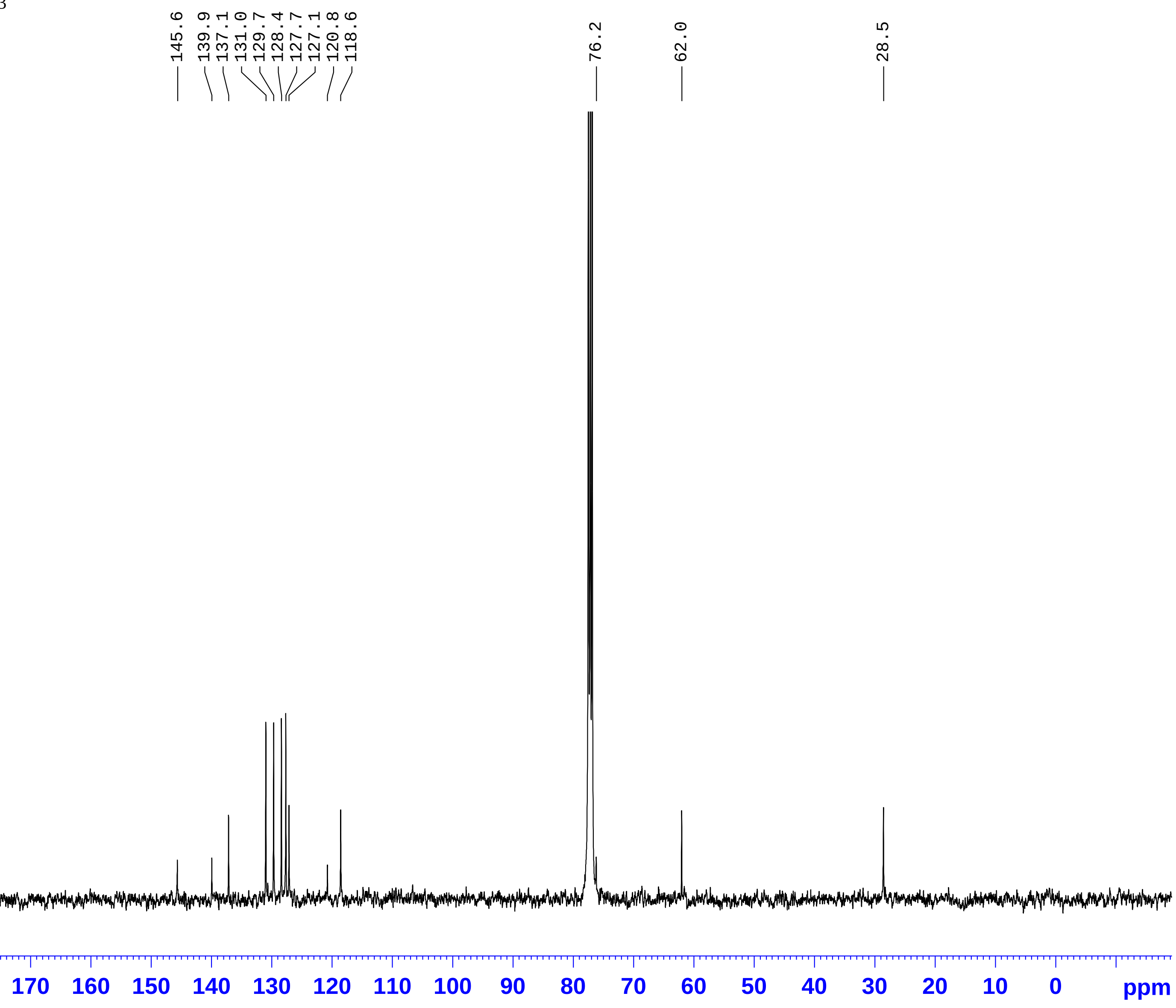

^{1}H NMR of **6d** in $CDCl_3$

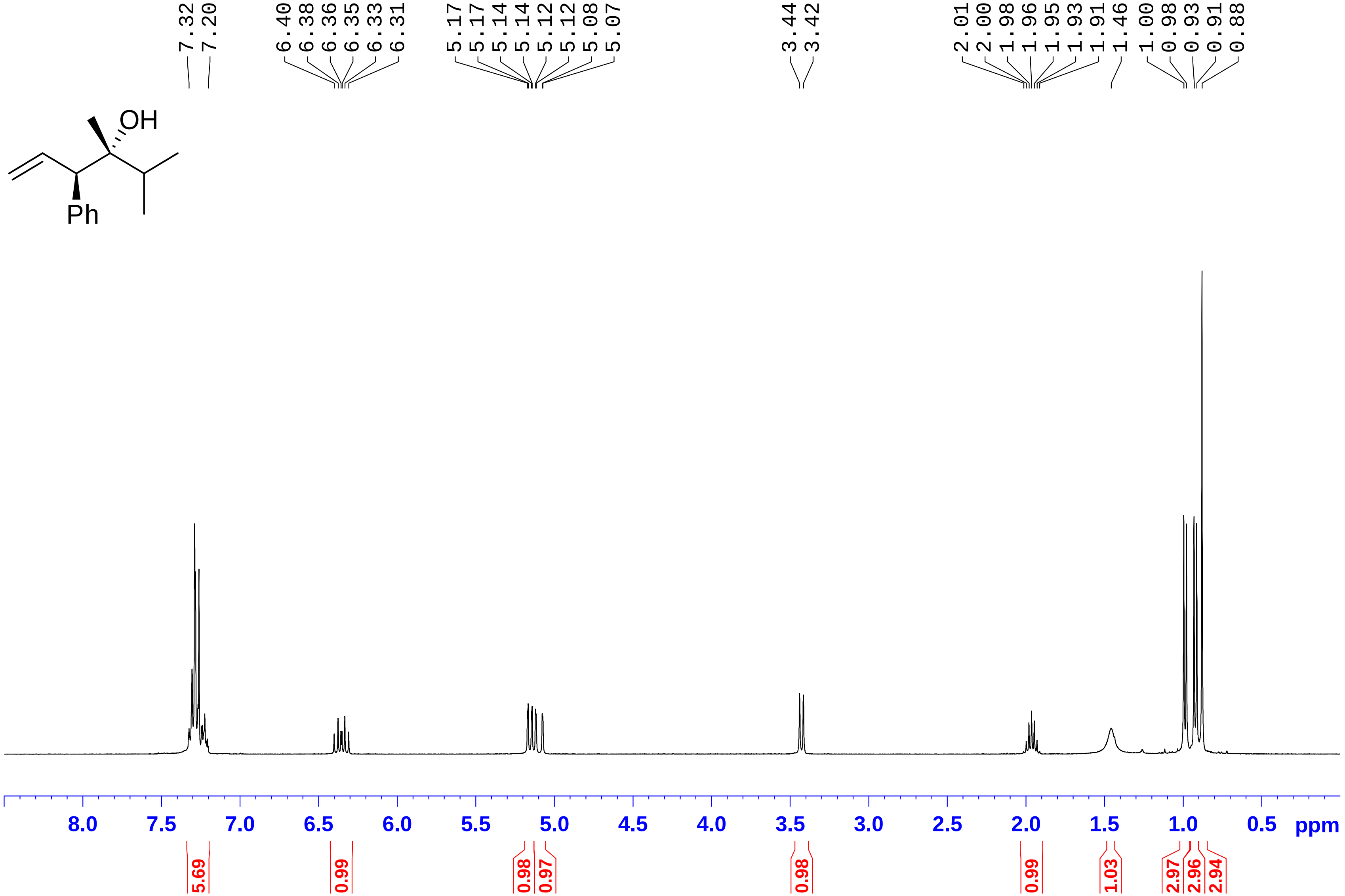

^{13}C NMR of **6d** in $CDCl_3$

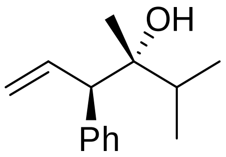

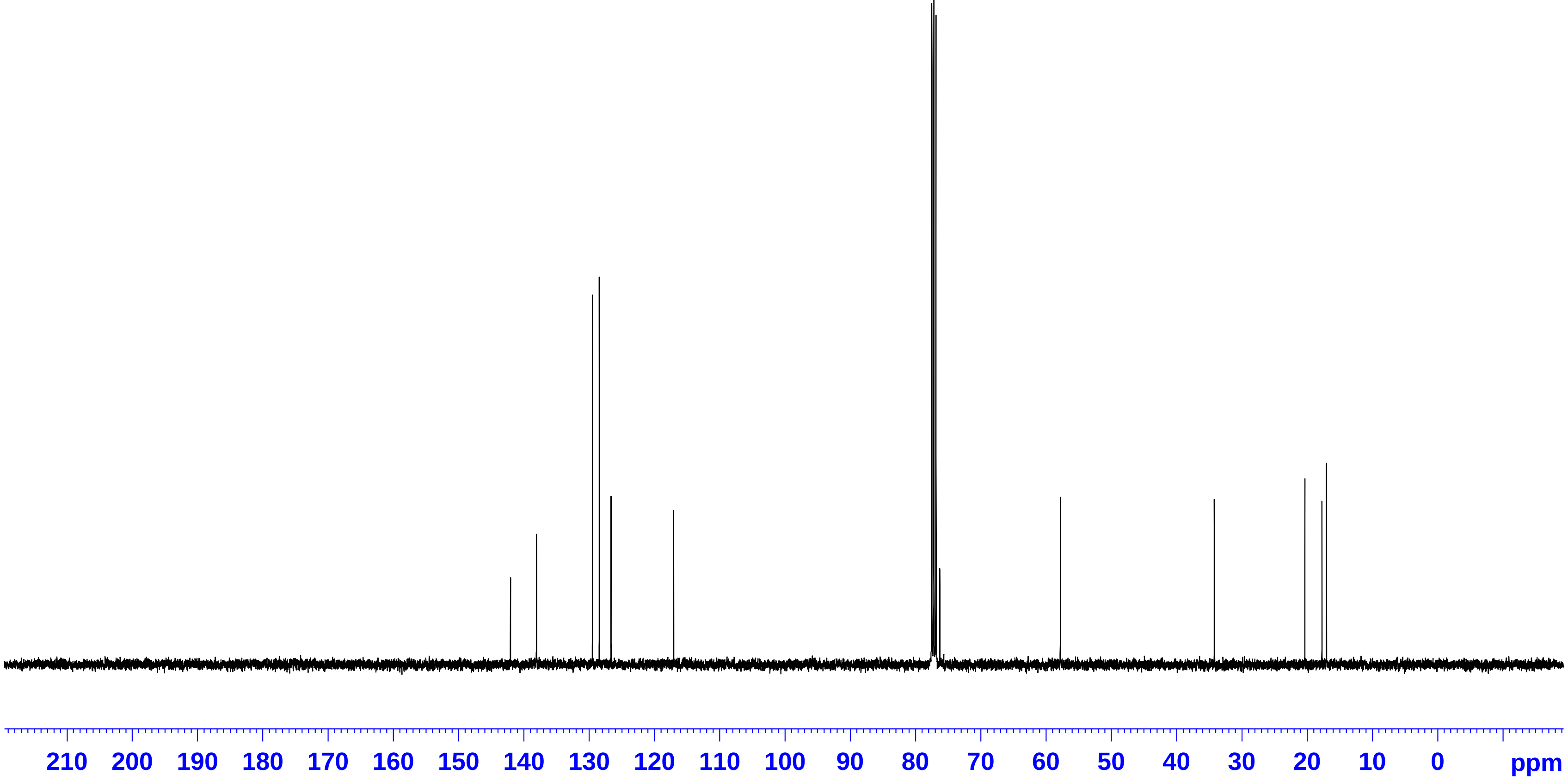

^{1}H NMR of **6e** in $CDCl_3$

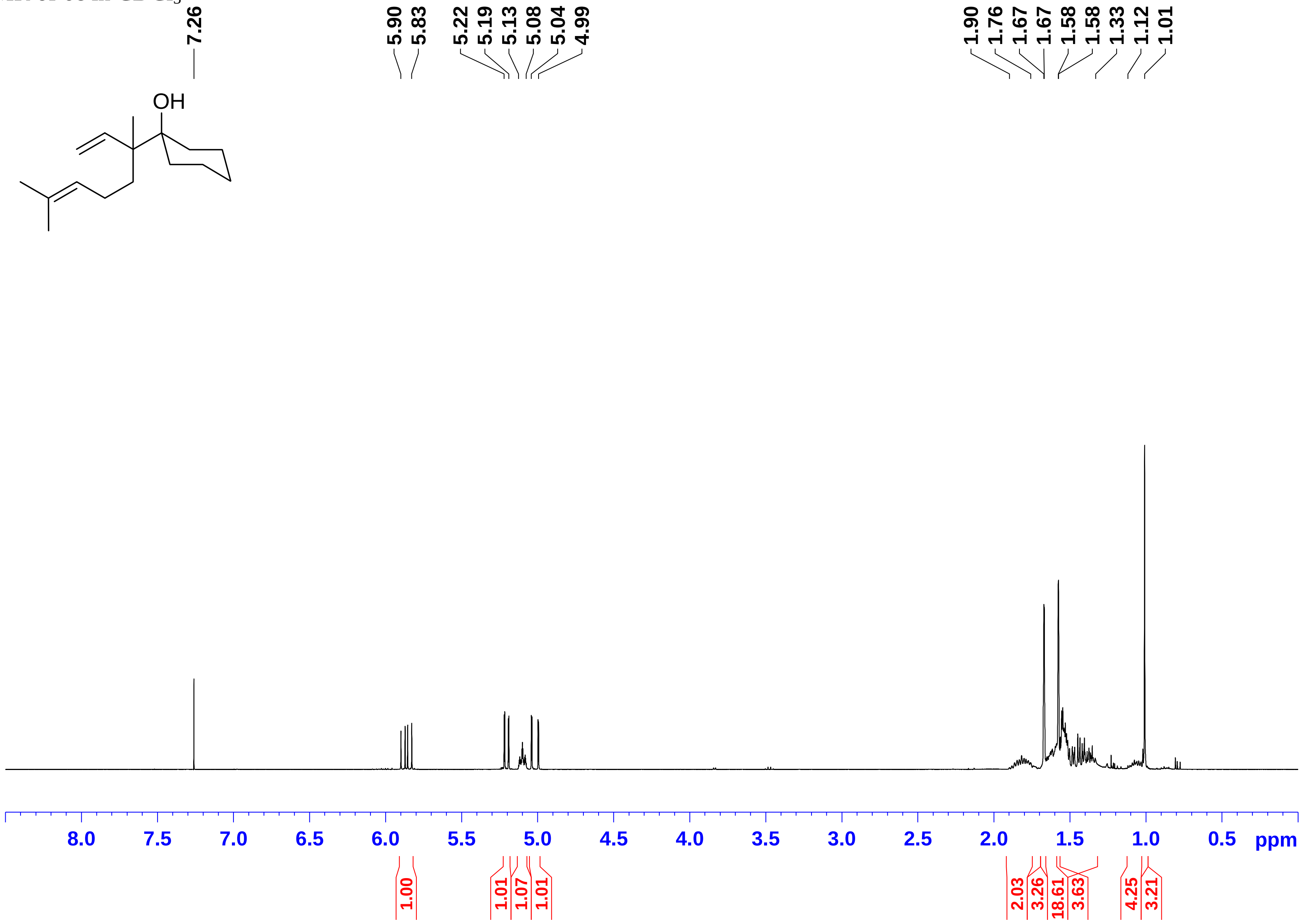

^{13}C NMR of **6e** in $CDCl_3$

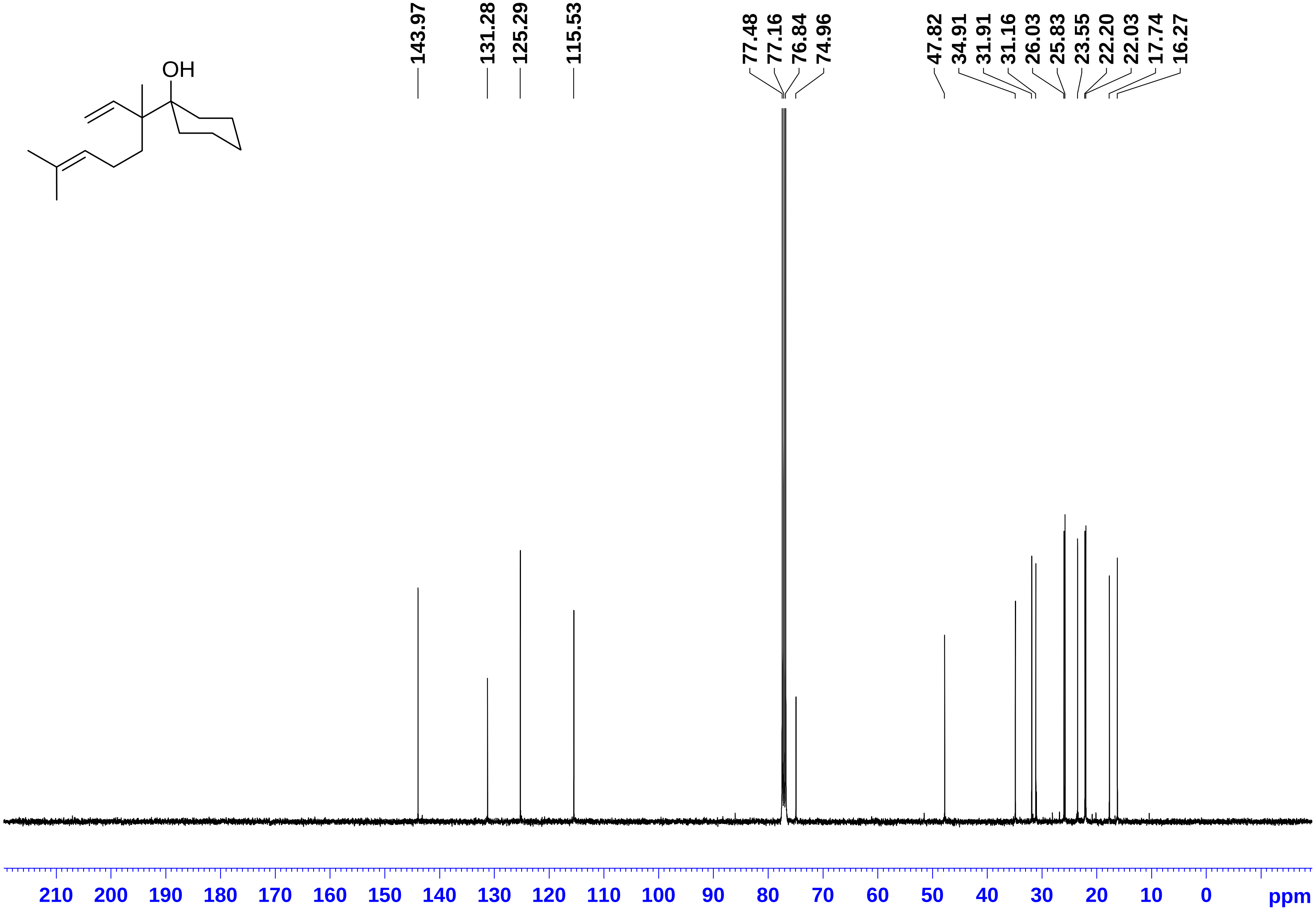

^{1}H NMR of **6f** in $CDCl_3$

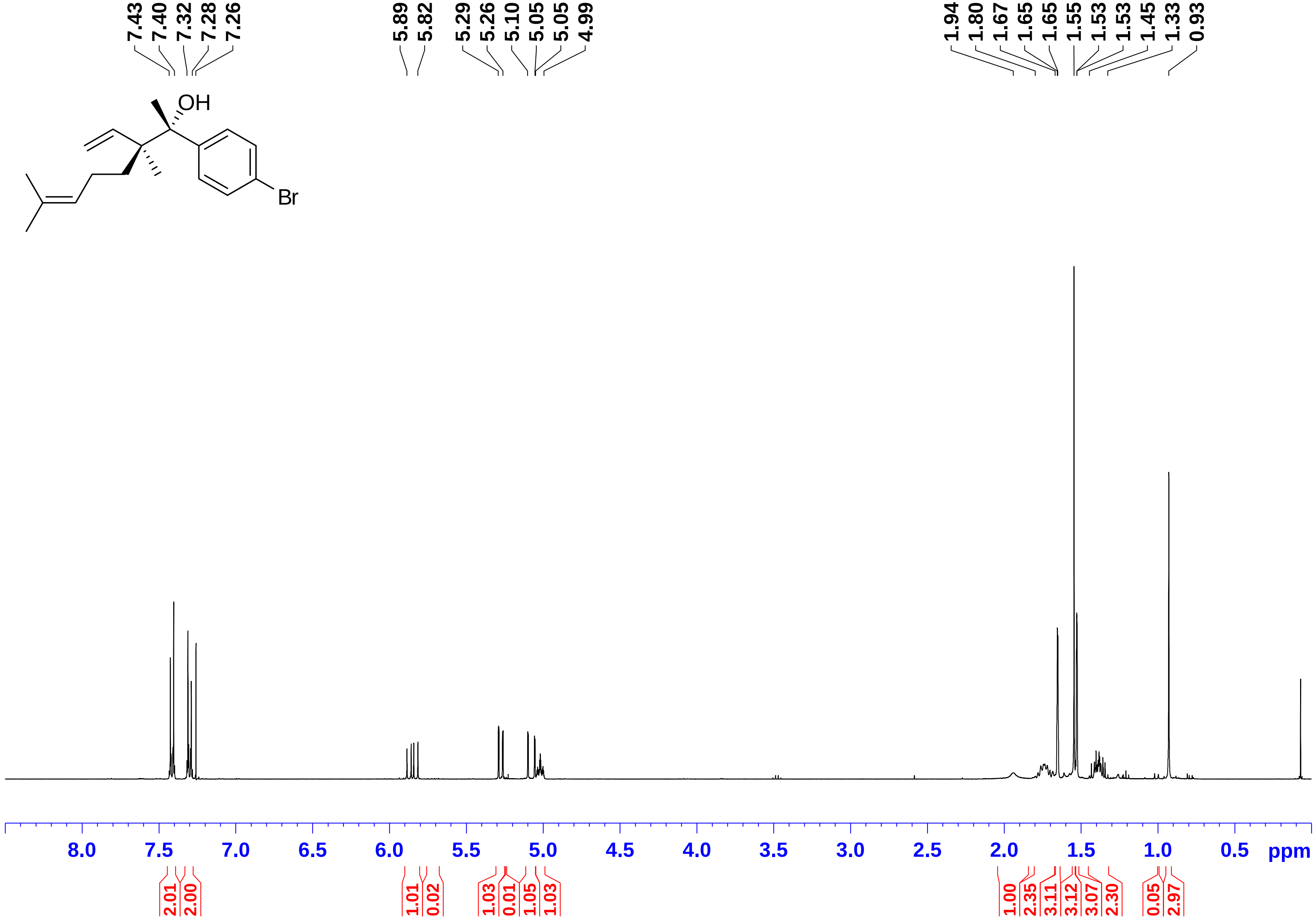

^{13}C NMR of **6f** in $CDCl_3$

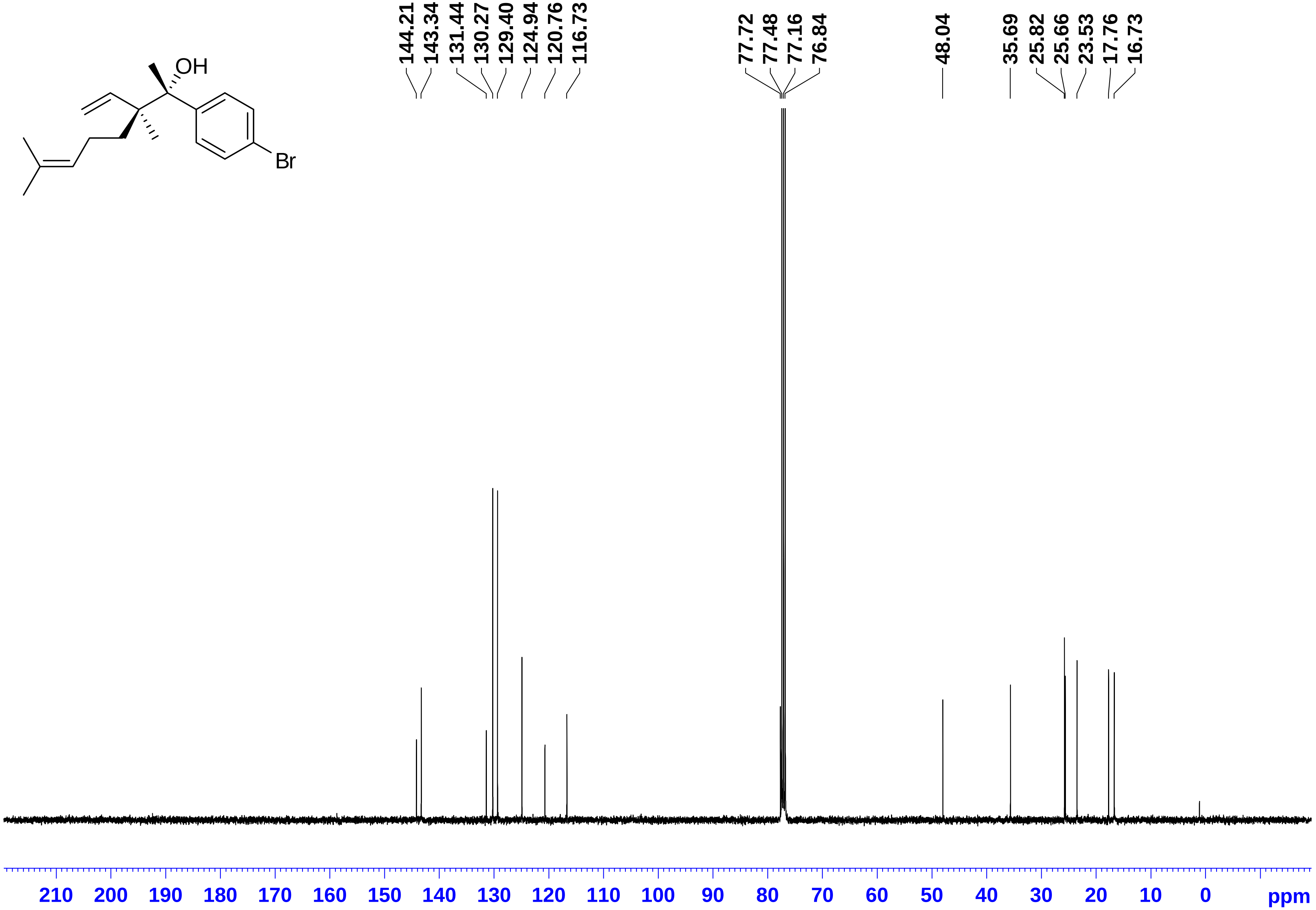

^{1}H NMR of **6g** in $CDCl_3$

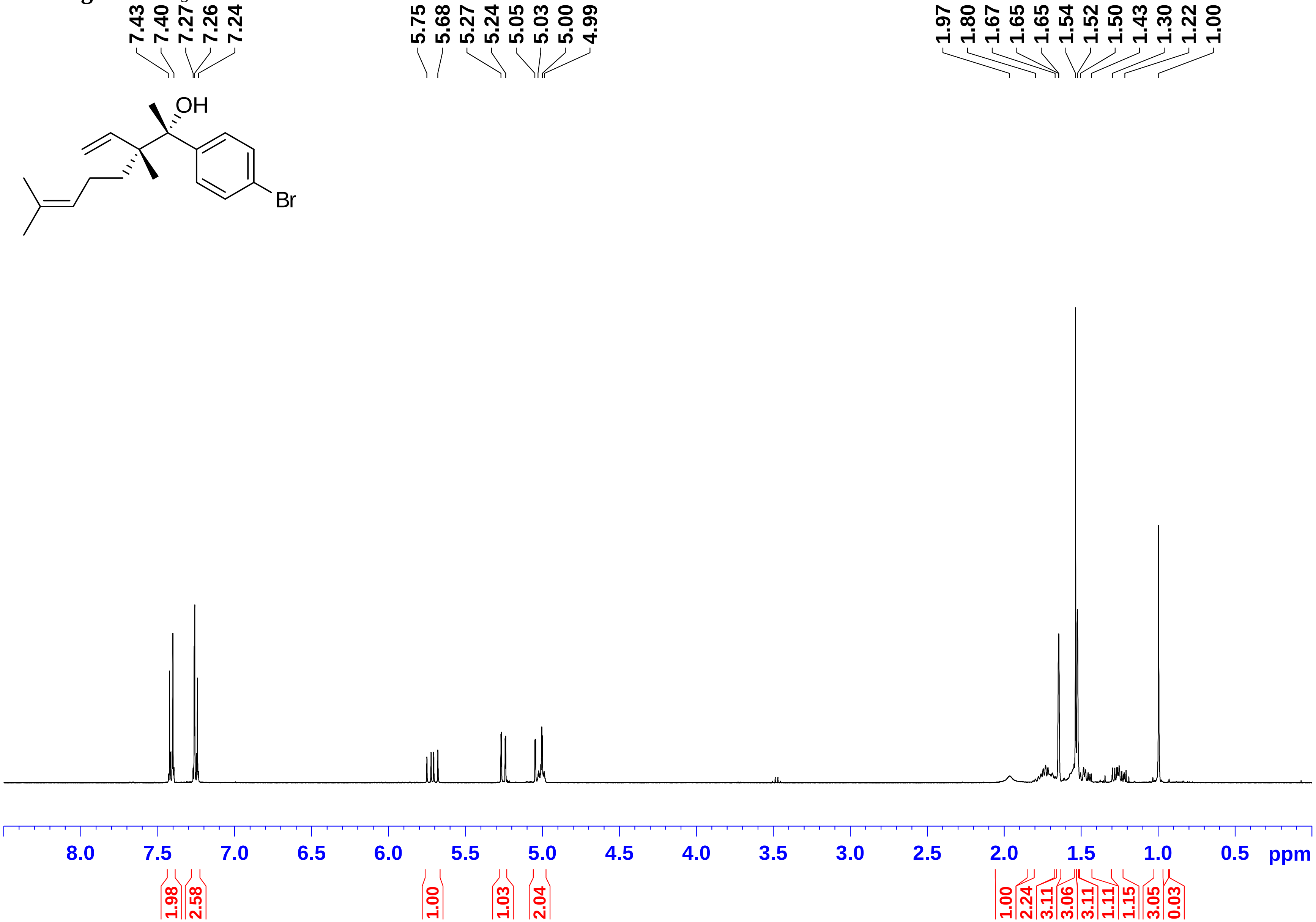

^{13}C NMR of **6g** in $CDCl_3$

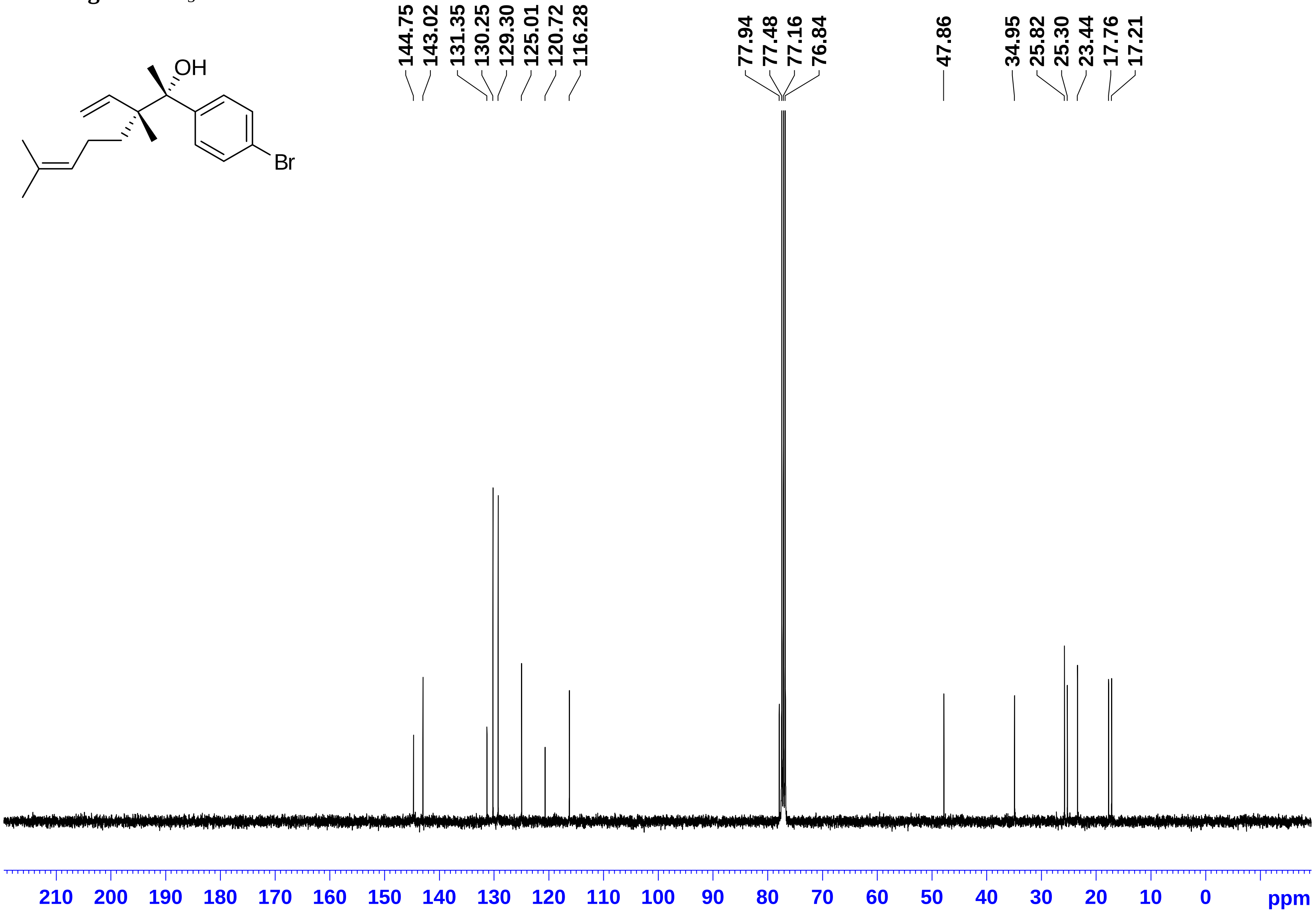

Comparision of ^{1}H NMR of **6f** (red) and **6g** (blue) in $CHCl_3/CDCl_3$

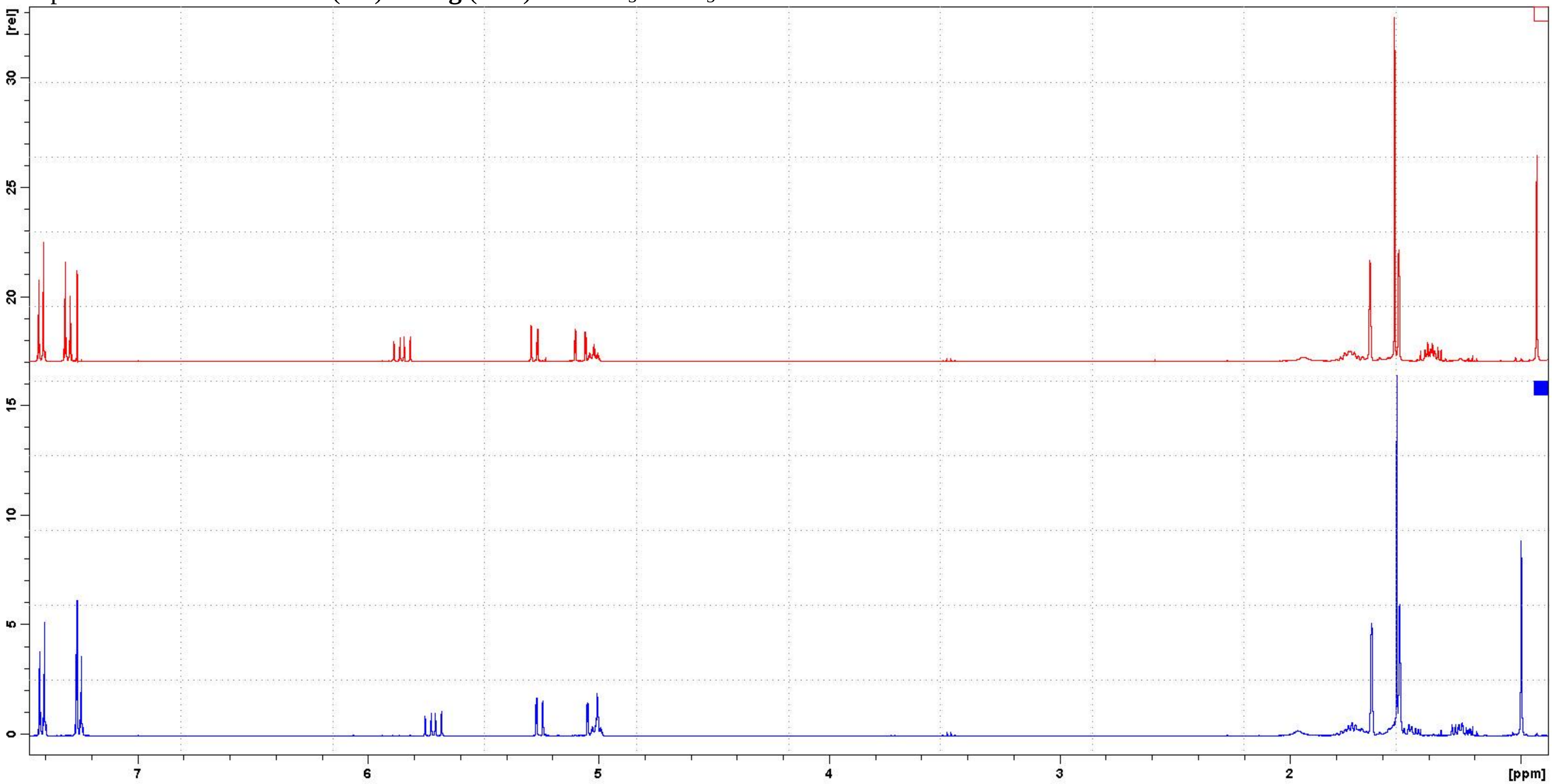

Comparision of ^{13}C NMR of **6f** (red) and **6g** (blue) in $CHCl_3/CDCl_3$

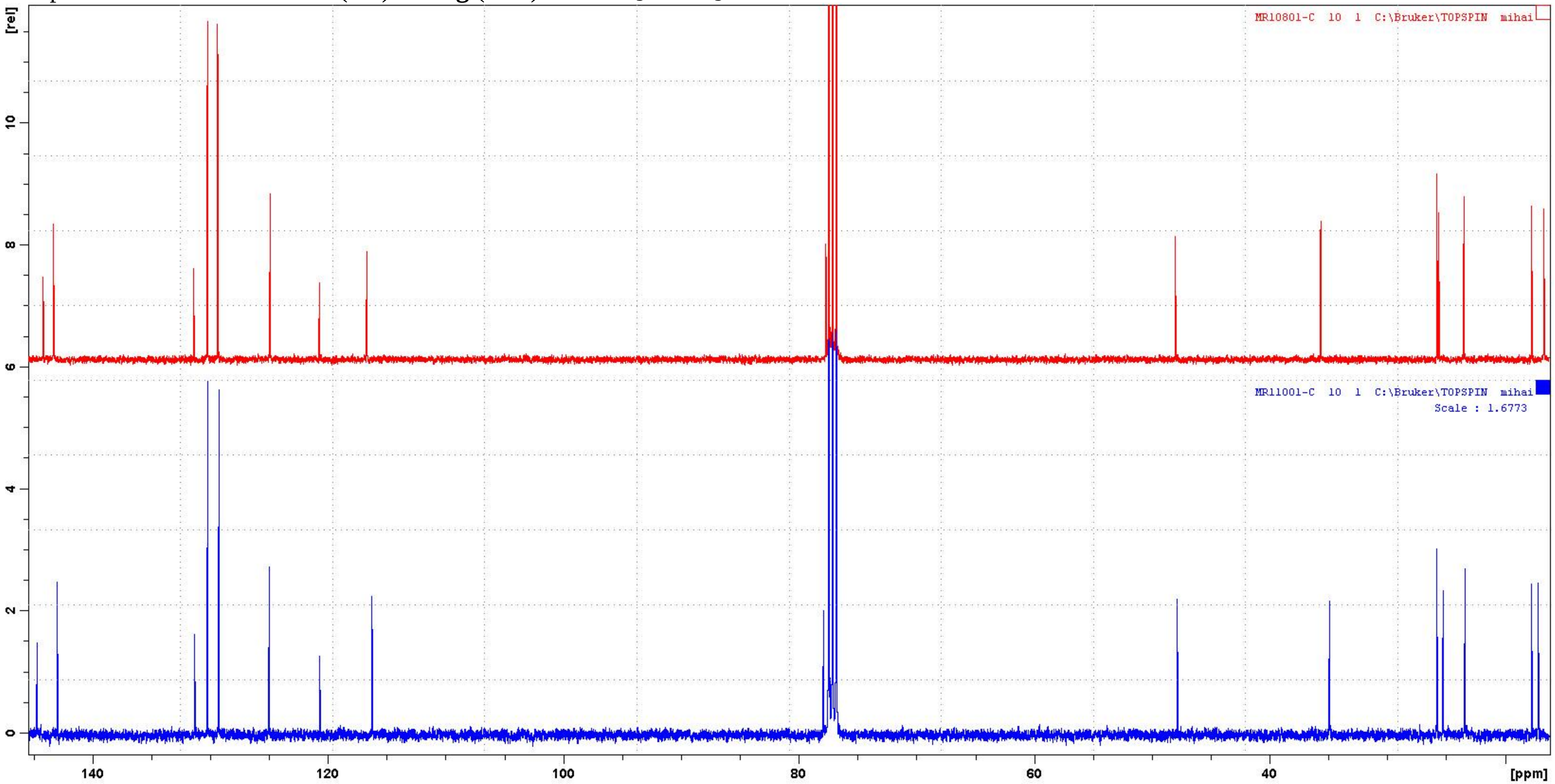

Supplement: Supplementary file 1 [file anie0051-13050-SD1.pdf]
